# Optimizing Intracellular Transport of Antimicrobial Metallohelices Delivers Selective Nanomolar Potency in *E. coli*

**DOI:** 10.1021/acs.inorgchem.5c05039

**Published:** 2026-01-03

**Authors:** Miles L. Postings, Nicola J. Rogers, Georgia Shreeve, Hualong Song, Guy Clarkson, Anish Mistry, John Moat, Grace Taylor-Joyce, Nicholas R. Waterfield, Peter Scott

**Affiliations:** † Department of Chemistry, 2707 University of Warwick, Gibbet Hill Road, Coventry CV4 7AL, U.K.; ‡ Department of Chemistry, 26679 Hong Kong Baptist University, Kowloon Tong, Kowloon 999077, Hong Kong; § Beijing Area Major Laboratory of Peptide and Small Molecular Drugs, Engineering Research Centre of Endogenous Prophylactic of Ministry of Education of China, Capital Medical University, Beijing 100069, China; ∥ School of Life Sciences, University of Warwick, Gibbet Hill Campus, Coventry CV4 7AL, U.K.; ⊥ Warwick Medical School, University of Warwick, Gibbet Hill Campus, Coventry CV4 7AL, U.K.

## Abstract

To investigate large discrepancies in antimicrobial potency between cationic amphipathic metallohelix architectures, 22 new optically pure candidates were synthesized via self-assembly. A total of 34 compounds were tested against *S. aureus*, *E. coli*, and, for the most active, against a panel of ESKAPE pathogens. While addition of substituents reduced activity in a 3-fold symmetric “flexicate” series, a potent compound (∼500 nM) with promising selectivity against a challenging *E. coli* microbe emerged in the hitherto inactive “triplex” series. This and other key compounds were studied by using techniques focused on transport and localization in Gram-positive and Gram-negative bacteria. Zeta-potential measurements at model membranes revealed affinities that mirror the antimicrobial activity. Extensive temperature- and concentration-dependent intracellular accumulation studies via isotopic labeling revealed that antimicrobial activity (within each architecture) is strongly dependent on the ability to enter the cell via passive diffusion. Mechanistic differences across metallohelix classes are confirmed by checkerboard activity assays and confocal microscopy studies via Click-labeled alkyne derivatives. The most active (and bactericidal) enantiomer achieves a growth-inhibiting concentration across the microbial population (apparently not restricted to dividing cells) at ca. 250 nM applied dose. Extraordinarily, given this very high potency, the mirror image of this compound is essentially inactive.

## Graphical abstract

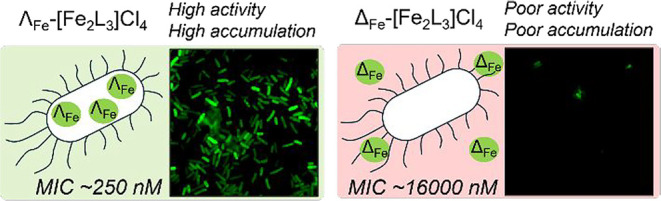

## Introduction

Antimicrobial resistance (AMR) is evolving into a global crisis, with a predicted mortality rate of 10 million p.a. and economic loss of up to $100 trillion by 2050.^1^ We are faced with an onslaught of drug-resistant bacteria, few novel drugs passing clinical trials, and difficultly in developing new antibiotics that circumvent cross-resistance to existing drugs (particularly against Gram-negative bacteria).^2–7^ There is thus an urgent need for new classes of antimicrobial agents.^8^ Compounds that are outside the traditional rule-of-five chemical space^9,10^ provide new opportunities, given their potential to address “difficult to drug” targets, e.g., large, flat, or groove-shaped binding sites such as those found, for example, in DNA or formed during protein–protein interactions.

Lehn^11^ suggested nearly four decades ago that linear multimetallic metal–ligand assemblies he termed helicates^12–15^ could play a role in drug discovery, but to date, few such molecules have emerged that could feasibly be used in this way, i.e., that are water-compatible, stable, and available in optically pure form without laborious processing, using a modular synthetic strategy that gives easy access to a wide range of compounds.^16^ To address this, we have reported several classes of helical metal–ligand assemblies,^17–23^ in which the absolute configuration is fixed via highly diastereoselective processes at the individual metal centers, thus giving optically pure compounds directly by self-assembly.^24^ The diamagnetic Fe­(II) compounds of this kind also have high stability to hydrolysis as a result of a combination of hydrophobic π-stacking, hydrogen bonding, and in some cases, cooperative mechanical coupling between the metal coordination units. This unique combination of properties, along with the ability to make many derivatives, has enabled extensive studies by ourselves and collaborators in the areas of cancer^18,20,23,25–28^ (notably binding DNA motifs such as G-quadruplexes^29,30^ ), Alzheimer’s disease,^31–33^ diabetes,^34^ gene delivery,^35^ Mpox virus,^36^ and inhibition of ice recrystallization.^37^ Overall, results in this area have led to the observation that the compounds emulate the properties of short cationic α-helical peptides: compounds with which they have a broad resemblance with regard to size, charge, and amphipathic nature.^16^

We thus wondered why so few of our metallohelices have shown antimicrobial activity.^17,22^ For example, while the prototypical Λ-**9a** (Scheme 1) showed minimum inhibitory concentration (MIC) as low as 2 μg mL^–1^,^17,22^ various related compounds were essentially inactive.^20^ In particular, the dozens of reported “triplex” compounds^23,25^ such as enantiomers of **11a**—with their antimicrobial peptide-like amphipathic architectures arising from bidirectional strand arrangement—gave no significant antimicrobial activity (MIC values >128 μg mL^–1^). Having recently established that metallohelices accumulate more readily in both *E. coli* and *S. aureus* cells when the central bridge is more hydrophobic (i.e., dibenzofuran cf. *meta*–xylene bridge between the Fe­(II) chelating sites),^38^ we began to investigate the effect of the addition of *peripheral* hydrophobic groups on the three architectures of Scheme 1. Substitution at the position Z shown was chosen since this is unlikely to perturb the structure of the core metallohelix units, which we presume to be the potential drug “payload.”

**Scheme 1:**
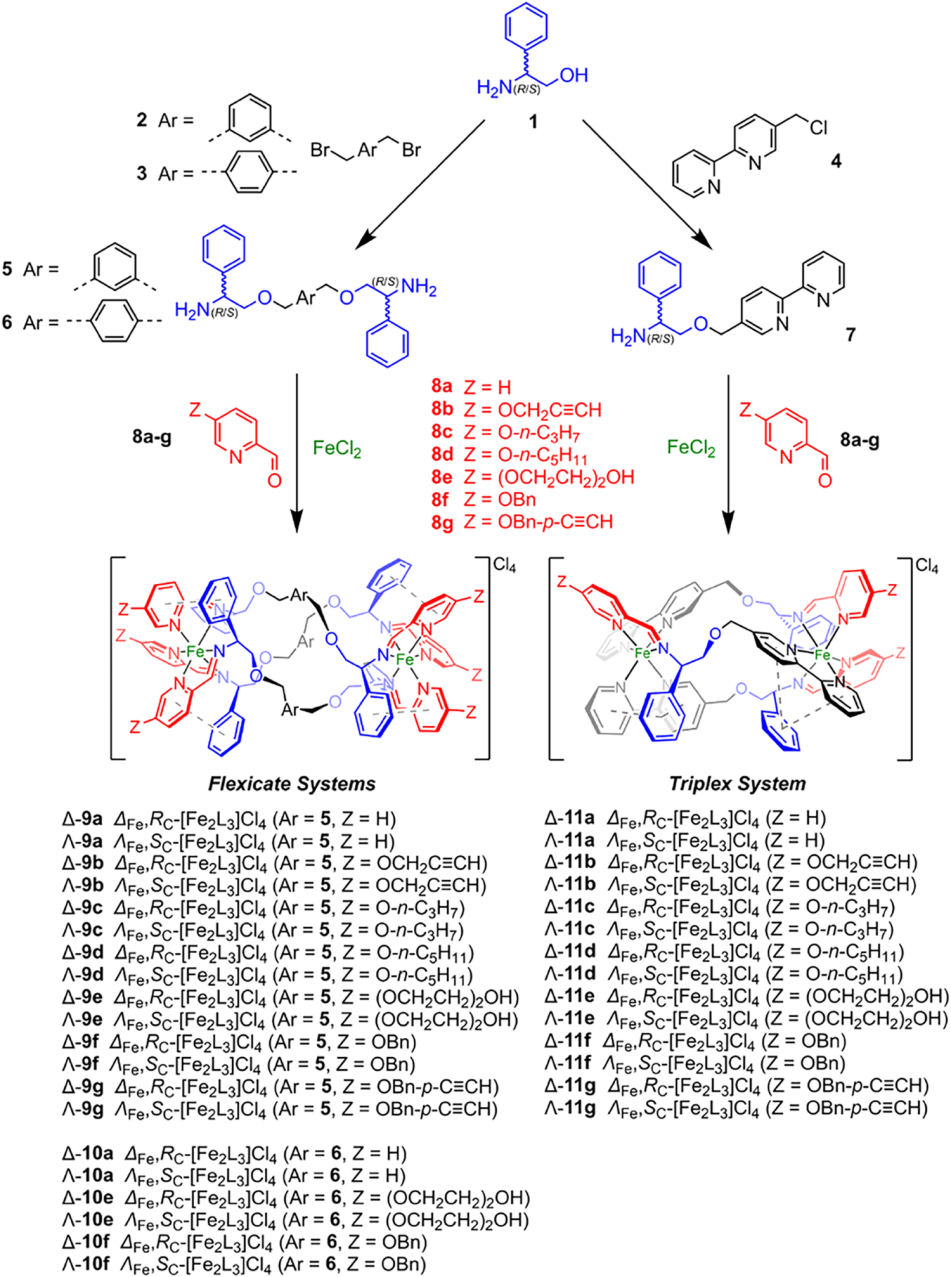
Synthesis of Metallohelices of Series 9–11 used in This Study

## Results and Discussion

### New Metallohelix Syntheses Reveal Anion Binding Motif

The six unsubstituted compounds **9a**–**11a** (Scheme 1) have been previously reported,^17,22,23^ and we have used alkyne compounds **9b** and **11b** in copper-catalyzed azide–alkyne click (CuAAC) chemistry, creating A range of derivatives.^22,25,26^ In the current work, aldehydes **8b**–**g** were synthesized from 5-hydroxypicolinaldehyde^39^ via Williamson etherification, and the 24 new metallohelices were subsequently allowed to self-assemble by heating compounds **8** with either the optically pure flexicate precursors **5** or **6**, or the triplex precursor **7**, and Fe­(II) chloride in the required stoichiometries, followed by simple workup. These reactions are very efficient and give single enantiomers of the product in each case, without the need for resolution. In the case of triplexes **11**, the ligand strands are bidirectional, i.e., head-to-head-to tail; note the positions of the three pyridine units colored red in Scheme 1. Characterization of complexes included multinuclear NMR, electrospray-ionization mass spectrometry (ESI-MS), Fourier-transform infrared spectroscopy (FTIR), UV–visible spectroscopy, microanalysis, and thermogravimetric analysis (TGA).

For each Fe­(II) “flexicate” complex (series **9** and **10**), a single set of ligands was observed in the ^1^H NMR spectrum, confirming that all products were bimetallic 3-fold symmetric structures with high stereochemical purities.^17^ While compounds of the Fe­(II) “triplex” series **11** have well-resolved ^13^C NMR spectra, many ^1^H NMR spectra were broader in comparison to the isostructural Zn­(II) analogues, which were able to be fully assigned (ESI). These spectra (e.g., Δ_Zn_-alkynylbenzyloxy complex [Figure 1b]) show, as a result of the bidirectional arrangement of the ligand strands and the helical twist, three spectroscopically unique ligand environments for each complex.

**Figure 1:**
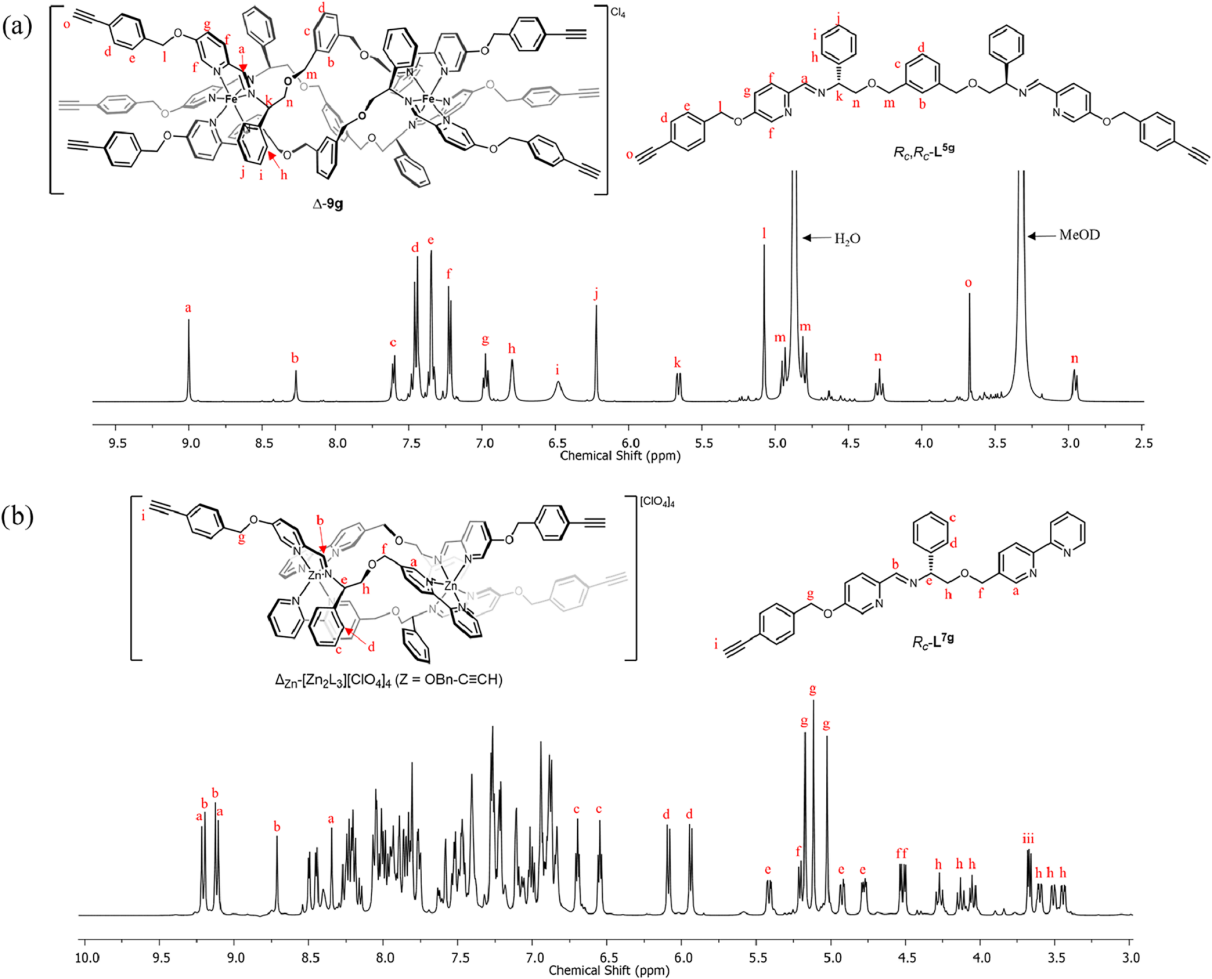
^1^H NMR spectra of (a) flexicate Δ-9g (500 MHz, 298 K, MeOD); (b) isostructural Zn­(II) analogue of Fe­(II) triplex Δ-11g (500 MHz, 298 K, CH_3_CN).

Strong peaks for the [Fe_2_L_3_]^4+^ tetracation were observed by ESI­(+)-MS in all cases, while high-resolution ESI-MS analysis revealed isotopic peaks for molecular ions separated by 0.25 Da. (see Figures S15–S25, SI). Circular dichroism spectra of aqueous solutions (0.03 mM) of the enantiomeric pairs of compounds displayed equal and opposite peaks. Water of crystallization, evident in the Fe compounds from the intense H_2_O peaks in the ^1^H NMR spectra and the broad O–H stretching bands in the 3000–3500 cm^–1^ region of the FTIR spectra, was quantified by thermogravimetric analysis in conjunction with microanalytical data.

While we were unable to grow crystals of the compounds of Scheme 1 suitable for X-ray diffraction, we found that slow vapor diffusion of ethyl acetate into a concentrated acetonitrile solution of a 1:1 mixture of the enantiomers Λ- and Δ_Zn_-[Zn_2_ **L**_3_^**5f**^]­[ClO_4_]_4_ afforded single crystals suitable for XRD of the Zn­(II) analogue of **9f**. The cationic unit with the surrounding perchlorate anions is shown in Figure 2. The structure of the core unit is the same as that of a previously reported parent compound,^22^ with the *m-*xylenyl units arranged such that three aryl C–H bonds point into the strongly deshielding central cavity of the complex; consequently these H atoms are observed at *ca* 8.3 ppm in the ^1^H NMR spectra (e.g., Figure 1a) in all of the Fe­(II) compounds of series **9**. Of the six perchlorate anions surrounding the tetracation, four (colorized yellow in Figure 2) have conventional locations, lying in the voids between the packed helices and close to unshielded areas of positive charge, as we have noted,^38^ while two (colorized pink) are associated with the terminal O–CH_2_–Ph groups via two short benzylic CH-anion contacts each. This anion binding provides a mechanism whereby the compounds of Scheme 1 that contain propargylic (labeled **b**) and benzylic ether (**f** and **g**) H-bonding units may reversibly reduce the overall charge of the assembly and thereby modulate affinity with hydrophobic or hydrophilic components of the cell envelope. Similar “chameleonic” features of macrocyclic peptides allow them to change their conformation in order to expose polar groups in aqueous solution but bury them when in a lower polarity environment.^40,41^ Subsequent results support this hypothesis.

**Figure 2:**
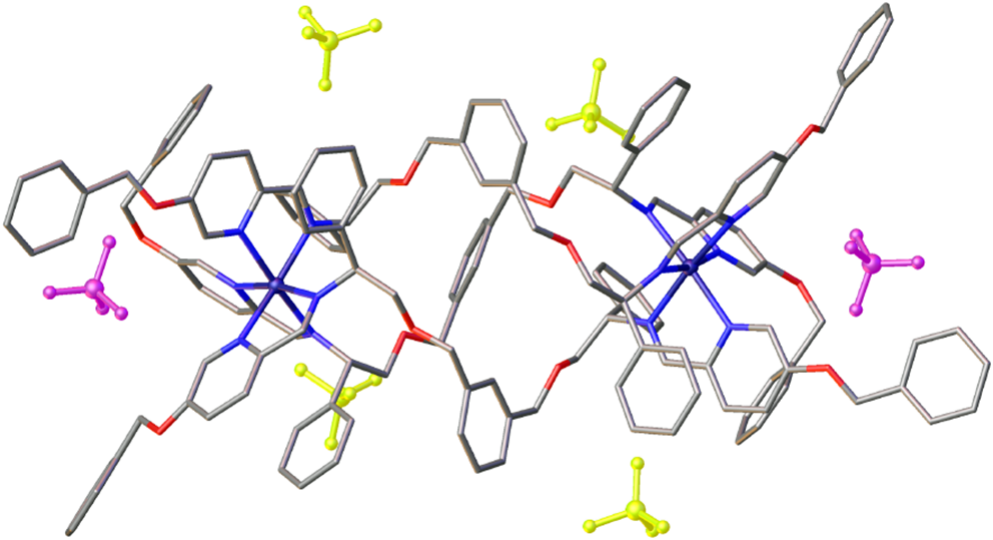
Structure of the cationic unit and perchlorate anions of the isostructural Zn­(II) analogue of Fe­(II) flexicate Δ-9f. Four perchlorate anions (colorized yellow) surround the cationic units as shown; two (colorized pink) are located in an H-bonding cavity formed by pendant benzyl units.

The flexicates of series **9** and **10** (Scheme 1) with the highest water solubility (pairs of compounds **a, b, e**, and **f**) plus the entire series of triplex compounds **11** were selected for kinetic stability studies. At 37 °C in various media, the compounds were generally stable: phosphate-buffered saline (PBS) (≤7% degradation after 24 h and ≤17% degradation after 28 d), HCl acid buffer (pH 1.5) (≤20% degradation after 12 h), and cation-adjusted Müller-Hinton broth (CAMHB) (≤15% degradation after 24 h). Correspondingly, no reduction in activity against *E. coli* was observed when compounds were stored in CAMHB for 4 d at the same temperature (Table S4, SI). Furthermore, these metallohelices were stable in the presence of excess deferoxamine (Figure S26, SI), indicating that strong chelators do not compete with our self-assembled ligand systems and reinforce that the intact compounds are the active species in biological assays.

### Triplex Enantiomer with Submicromolar Activity against *E. coli*

The 34 metallohelices in Scheme 1 were initially tested for antimicrobial activity against Gram-positive *S. aureus* ATCC 29213 and Gram-negative *E. coli* ATCC 25922. Minimum inhibitory concentrations (MICs) were determined using standard broth microdilution microbial assays (Table 1), following CLSI guidelines.^42,43^

**Table 1:** In Vitro MIC Values (μg mL^–1^) of Flexicates and Triplexes against *S. aureus* ATCC 29213 and *E. coli* ATCC 25922 Bacterial Strains

compound	Z =	MIC *S. aureus* ATCC 29213	MIC *E. coli* ATCC 25922
Λ-**9a**	H	16	4
Δ-**9a**	H	32	8
Λ-**9b**	OCH_2_C≡CH	8	8
Δ-**9b**	OCH_2_C≡CH	4	8
Λ-**9c**	O-*n*-C_3_H_7_	256	>256
Δ-**9c**	O-*n*-C_3_H_7_	>256	>256
Λ-**9d**	O-*n*-C_5_H_11_	128	>256
Δ-**9d**	O-*n*-C_5_H_11_	128	>256
Λ-**9e**	(OCH_2_CH_2_)_2_OH	256	256
Δ-**9e**	(OCH_2_CH_2_)_2_OH	>256	>256
Λ-**9f**	OBn	16	64
Δ-**9f**	OBn	32	64
Λ-**9g**	OBn-C≡CH	>256	>256
Δ-**9g**	OBn-C≡CH	>256	>256
Λ-**10a**	H	16	8
Δ-**10a**	H	16	8
Λ-**10e**	(OCH_2_CH_2_)_2_OH	>256	>256
Δ-**10e**	(OCH_2_CH_2_)_2_OH	64	128
Λ-**10f**	OBn	>256	>256
Δ-**10f**	OBn	64	>256
Λ-**11a**	H	>256	>256
Δ-**11a**	H	>256	>256
Λ-**11b**	OCH_2_C≡CH	256	16
Δ-**11b**	OCH_2_C≡CH	256	64
Λ-**11c**	O-*n*-C_3_H_7_	64	8
Δ-**11c**	O-*n*-C_3_H_7_	128	>256
Λ-**11d**	O-*n*-C_5_H_11_	32	2
Δ-**11d**	O-*n*-C_5_H_11_	32	32
Λ-**11e**	(OCH_2_CH_2_)_2_OH	256	>256
Δ-**11e**	(OCH_2_CH_2_)_2_OH	256	>256
Λ-**11f**	OBn	8	2
Δ-**11f**	OBn	8	8
Λ-**11g**	OBn-C≡CH	128	1
Δ-**11g**	OBn-C≡CH	128	64
Controls	Ampicillin	-	4
	Tetracycline	0.5	1
	Ticarcillin	4	8

We see that in most cases, addition of substituents in the flexicate series **9** and **10** significantly reduced activity in both microbes compared with the parent enantiomer pairs **9a** and **10a**. The propargyloxy compounds **9b** are notable exceptions, and Δ-**9b** at 4 μg mL^–1^ is *ca* eight times more active than Δ-**9a** against *S. aureus*.

Turning to the triplex series **11**, we confirmed^23^ that the parent compounds **11a** exhibited negligible activity (MIC > 256 μg mL^–1^). Here, however, in contrast with series **9**, the addition of hydrophobic substituents to the pyridine unit generally led to improvement. Against *S. aureus*, MIC values as low as 8 μg mL^–1^ were observed for both enantiomers of benzyloxy compound **11f**, while alkynylbenyloxy **11g** was 16 times less potent. Against *E. coli*, most substituents improved activity cf. **11a**, and at 1 μg mL^–1^ (ca. 500 nM), the activity of alkynylbenzyloxy Λ-**11g** is the highest yet reported for any related compound, and is comparable to the activities of clinically used *E. coli* antimicrobials.^42^ In contrast with the observations for *S. aureus*, there were substantial enantiomer effects with the Λ compounds consistently outperforming Δ. Strikingly, Λ-**11g** was *ca* 64 times more potent than its mirror image. These trends are considered below in the context of intracellular accumulation studies.

Metallohelices that demonstrated good antimicrobial activity by this measure were also tested in bacterial lethality assays. Minimum bactericidal concentrations (MBC)—the lowest concentration required to eradicate all viable cells—were determined, and since MBC/MIC ≤4 in all cases, the compounds are deemed^44^ to be bactericidal rather than bacteriostatic.

### Performance against ESKAPE Pathogens

On the basis of the results above, selected compounds from the series 9 and 11 were tested against B. subtilis plus four ESKAPE pathogens^5^ ^,^ ^45–47^ (Gram-positive *E. faecium* and Gram-negative *K. pneumoniae*, *P. aeruginosa*, and *A. baumannii*) (Table 2). The parent structures of **9a** showed activity across both Gram-positive and Gram-negative bacteria, with the exception of the notoriously drug-resistant Gram-negative ESKAPE pathogens *P. aeruginosa* and *A. baumannii*. Propargyloxy functionalization (9b) led to some improvement in activity against Gram-positive strains and *K. pneumoniae*, while the similar benzyloxy systems (9f) were slightly less potent. No significant activity was displayed by the more hydrophilic compound **9e**. As expected, triplex parent compound **11a** was inactive across all bacteria. Unsurprisingly, the Gram-negative ESKAPE pathogens were found to be generally difficult targets, although interestingly, *K. pneumoniae* appeared to be susceptible to Λ-**11g** (MIC = 16 μg mL^–1^) but not the enantiomer Δ-**11g** (>256 μg mL^–1^). Generally, again, the Λ enantiomers of series **11** outperformed their Δ counterparts by at least 4-fold in activity against Gram-negative bacteria.

**Table 2:** In Vitro MIC Values (μg mL^–1^) of Flexicates, Triplexes, and Appropriate Controls against the Gram-Positive Species *S. aureus* ATCC 29213, *B. subtilis* 168, and *E. faecium* SKB and against Gram-Negative Species *K. pneumoniae* K6, *P* *. aeruginosa* ATCC 27853, and *A. baumannii* BAA-1605

		gram-positive MICs			gram-negative MICs			
compound	Z=	*S. aureus* ATCC 29213	*B. subtilis* 168	*E. faecium* SKB	*E. coli* ATCC 25922	*K. pneumoniae* K6	*P. aeruginosa* ATCC 27853	*A. baumannii* BAA-1605
Λ-**9a**	H	16	4	4	4	32	256	128
Δ-**9a**	H	32	2	8	8	16	256	128
Λ-**9b**	OCH_2_C≡CH	8	2	8	8	64	128	256
Δ-**9b**	OCH_2_C≡CH	4	2	8	8	32	128	>256
Λ-**9e**	(OCH_2_CH_2_)_2_OH	256	64	128	256	>256	>256	>256
Δ-**9e**	(OCH_2_CH_2_)_2_OH	>256	>256	256	>256	>256	>256	>256
Λ-**9f**	OBn	16	16	16	64	>256	256	>256
Δ-**9f**	OBn	32	16	16	64	>256	128	>256
Λ-**11a**	H	>256	>256	256	>256	>256	>256	>256
Δ-**11a**	H	>256	256	256	>256	>256	>256	>256
Λ-**11b**	OCH_2_C≡CH	256	16	256	16	128	>256	>256
Δ-**11b**	OCH_2_C≡CH	256	16	256	64	>256	>256	>256
Λ-**11e**	(OCH_2_CH_2_)_2_OH	256	64	256	>256	>256	>256	256
Δ-**11e**	(OCH_2_CH_2_)_2_OH	256	128	256	>256	>256	>256	256
Λ-**11f**	OBn	8	4	16	2	64	256	128
Δ-**11f**	OBn	8	8	32	8	256	>256	>256
Λ-**11g**	OBn-C≡CH	128	32	8	1	16	64	128
Δ-**11g**	OBn-C≡CH	128	64	8	64	>256	>256	256

### Low Red Blood Cell Toxicity and High Selectivity for *E. coli*

Promisingly, low toxicities against equine red blood cells were indicated in a standard assay, with the new compounds tested having hemolytic concentrations ≥256 μg mL^–1^ (Table S3, SI).

A colorimetric MTT assay using ARPE-19, an immortalized human cell line, was also employed to investigate any cytotoxicity. A dimensionless selectivity index SI = IC_50_/MIC is presented in Table 3 for selected combinations of compound and bacteria as an indicator of relative performance against prokaryotes. While there were no outstanding performers in terms of the Gram-positive *E. faecium* selectivity over the ARPE cells, the compounds Λ-**11f** and Λ-**11g** have, by some margin, the best selectivity for *E. coli*. Notably, their mirror images had little or no selectivity as a result of this measure.

**Table 3:** In Vitro Human Cell Line Cytotoxicity (IC_50_ Values, μM) and Selectivity Index of New Flexicates and Triplexes using Non-Cancerous ARPE-19 Cell Line

		selectivity index = ARPE-19 IC_50_ (μM)/MIC (μM)	
compound	ARPE-19 IC_50_ (μM)	*E. faecium*	*E. coli*
Λ-**9a**	6.39 ± 2.36	3.3	3.3
Δ-**9a**	22.44 ± 2.56	5.7	5.7
Λ-**9b**	23.10 ± 0.72	6.9	6.9
Δ-**9b**	17.59 ± 1.55	5.2	5.2
Λ-**11b**	73.81 ± 13.05	0.6	8.9
Δ-**11b**	100.44 ± 4.67	0.8	3.0
Λ-**11f**	17.24 ± 2.98	2.0	16
Δ-**11f**	7.75 ± 1.45	0.5	1.8
Λ-**11g**	15.81 ± 3.28	4.0	32
Δ-**11g**	15.95 ± 2.45	4.0	0.5

### Affinity for Model Membranes Correlates with Activity

Given the above proposal that the compounds may interact to different degrees with bacterial membranes, we studied the interactions of **9a**, **9f**, **11a**, and **11f** with synthetic vesicles based on the lipid composition of the outer leaflet of *S. aureus* and *E. coli* inner membranes. Using recently established protocols,^38^ changes in vesicle surface charge were monitored in zeta-potential titrations. The results for the Λ enantiomers are shown in Figure 3; those for the Δ compounds were very similar (Figure S27, SI). In all cases, the addition of the cationic metallohelices to these net anionic vesicles led, as expected, to an increase in the surface charge, with the degree of change mirrored in the observed antimicrobial activity. Accordingly, we see that the benzyloxy triplex compound Λ-**11f** (MIC = 2 μg mL^–1^) interacts more strongly with the model *E. coli* membrane than does the inactive parent compound Λ-**11a** (Figure 3a). A similar behavior is observed with the *S. aureus* membrane model, albeit at higher concentrations (Figure 3b). For the flexicates, we see that Λ-**9f** (MIC = 64 μg mL^–1^) had substantially less effect on the zeta-potential of the *E. coli* model vesicles than Λ-**9a** (MIC = 4 μg mL^–1^) (Figure 3a), at least at the lower concentrations, and Λ-**9a** and Λ-**9f**, both with MIC of 16 μg mL^–1^ in *S. aureus*, induce similar zeta-potential profiles in the *S. aureus* model (Figure 3b). It is important to note that for the active compounds discovered here, membrane disruption was not observed, so we see these zeta-potential profiles as indicators of membrane affinity rather than propensity to disrupt membranes.

**Figure 3:**
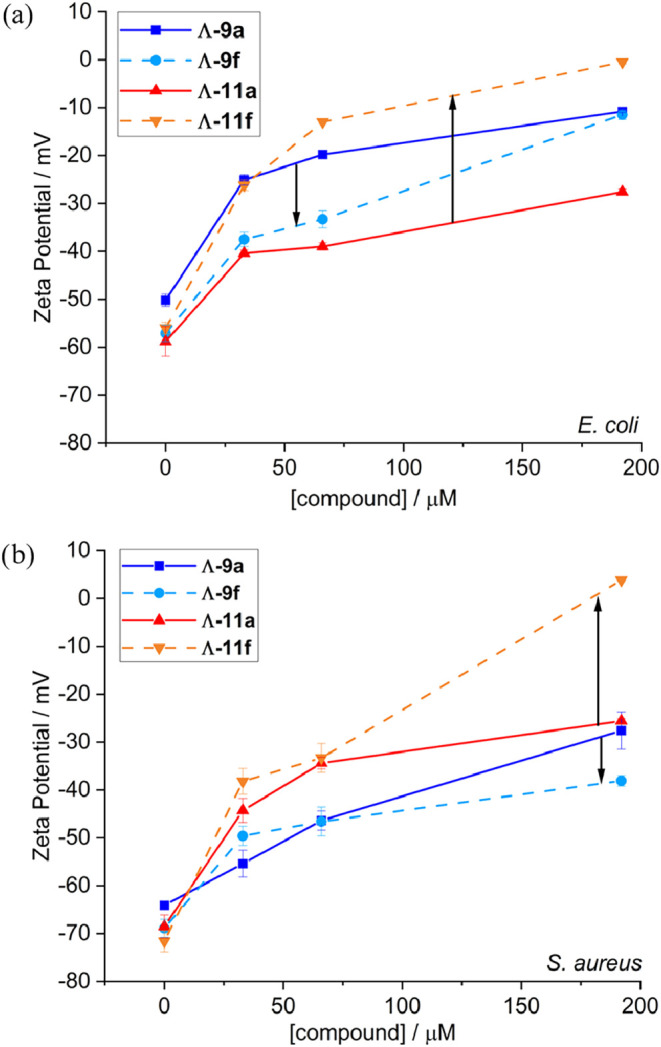
Zeta-potential measurements for addition of metallohelices to membrane-mimetic unilamellar vesicle models based on (a) *E. coli* and (b) *S. aureus* outer leaflet of inner membranes. Lipids at 0.5 mg mL^–1^ (∼0.6 mM) in sodium phosphate buffer (25 mM, pH 7.4) at 25 °C. Mean of five independent measurements ±1 standard deviation.

### Antimicrobial Activity Correlates with Degree of Passive Intracellular Accumulation

We conducted a quantitative study on the intracellular accumulation of 16 selected compounds, isotopically labeled by using 57Fe iron­(II) chloride as the metal source. For clarity, ^57^Fe isotopologues of the metallohelices are denoted by adding a prime (′) to the compound label (e.g., 9b′ for the ^57^Fe isotopologue of 9b). Bacterial inhibition assays of these ^57^Fe complexes were performed, and the MIC values were found to be the same as the corresponding ^56^Fe compounds. Cultures of bacteria were treated with ^57^Fe complexes at specified concentrations and incubation temperatures for 30 min before pelleting by centrifugation and the removal of supernatant. The cell pellets were washed twice to remove any extracellular compound, before digestion in nitric acid and analysis by ICP-MS. A preliminary study was conducted at 373 K and at an 8 mg mL^–1^ compound dosage, regardless of the antimicrobial potency.

In *S. aureus* (Table 4), it was found that while the parent enantiomers of **9a′** accumulated at *ca* 10 ng ^57^Fe per 10^8^ cells (henceforth ng), the more potent propargyloxy derivatives **9b′** achieved *ca* 4–6 times higher concentration, while the inactive alkoxy compound **9d′** was present at negligible levels. These results are consistent with the chameleonic anion binding model described above in that the alkoxy compound will be a poorer H-bond donor. Further, we note that the significantly higher level of accumulation of Δ-**9b′** in comparison to its mirror image follows the same trend as that of the observed MICs. Similarly, the inactive triplex compounds **11′** showed no significant accumulation at this concentration, while the modestly active compound Λ-**11d′** gave low but reproducible measurements.

**Table 4:** Iron (^57^Fe) Cellular Accumulation (ng ^57^Fe per 10^8^ cells) in *S. aureus* USA300 and *E. coli* TOP10 Bacteria when Dosed with 8 μg mL^‑1^ of Select Flexicates and Triplexes^a^

		*S. aureus* USA300		*E. coli* TOP10	
compound	Z=	MIC	^57^Fe conc. at 8 μg ml^–1^	MIC	^57^Fe conc. at 8 μg ml^–1^
Δ-**9a′**	H	16	10.0 ± 0.2	4	77.3 ± 4.3
Λ-**9a′**	H	16	10.9 ± 0.2	2	138 ± 4
Δ-**9b′**	OCH_2_C≡CH	4	59.3 ± 3.1	8	41.2 ± 1.1
Λ-**9b′**	OCH_2_C≡CH	8	36.7 ± 2.7	8	51.6 ± 22.1
Λ-**9d′**	O-*n*-C_5_H_9_	128	1.24 ± 0.23	>256	0.79 ± 0.06
Δ-**11a′**	H	>256	0.03 ± 0.04	>256	0.22 ± 0.03
Λ-**11a′**	H	>256	0.07 ± 0.03	>256	0.29 ± 0.05
Δ-**11b′**	OCH_2_C≡CH	128	0.21 ± 0.04	64	0.13 ± 0.06
Λ-**11b′**	OCH_2_C≡CH	256	0.09 ± 0.06	8	21.6 ± 4.1
Λ-**11d′**	O-*n*-C_5_H_9_	64	1.37 ± 0.05	4	22.5 ± 2.7

a In control experiments, untreated *S. aureus* and *E. coli* cells had 0.20 ± 0.07 and 0.34 ± 0.07 ng of ^57^Fe (10^8^ cells)^−1^, respectively. MIC values reported in μg mL^–1^.

In *E. coli*, we again saw a very strong concurrence between compound accumulation at 8 μg mL^–1^ and observed MIC; the enantiomer Λ-**9a′** achieves higher accumulation than Δ-**9a′** at this concentration and has the higher activity, while the slightly less active propargyls **9b′** achieved lower accumulations, and inactive **9d′**, almost undetectable. In the triplex compounds also, only active compounds Λ-**11b′** and Λ-**11d′** showed significant accumulation at this applied concentration.

These results led us to undertake studies using MIC concentrations of the compounds, so that the accumulation of ^57^Fe would indicate the concentration of antimicrobial compound required to achieve inhibition, presuming, given the required washing protocol, that the target is intracellular. These results are shown in Figure 4 (red bars). We also conducted the full range of studies at an incubation temperature of 277 K, and as can be seen (blue bars), this led generally to a reduction in accumulation of <10% across the series of compounds and both microbial strains. Further, and again with great consistency across all compound/strain combinations, dosing at 50% MIC led to *ca* 50% reduction in ^57^Fe accumulation (pink bars). The results strongly imply that the compounds enter the microbial cells via mechanisms that are principally equilibrative in nature, i.e., not ATP-dependent active transport mechanisms. This has been observed for some nonlytic antimicrobial peptides.^48^ Further, the consistency of these results across the panels of compounds and microbes and also across the conditions applied gives us confidence in the absolute values and the trends.

**Figure 4:**
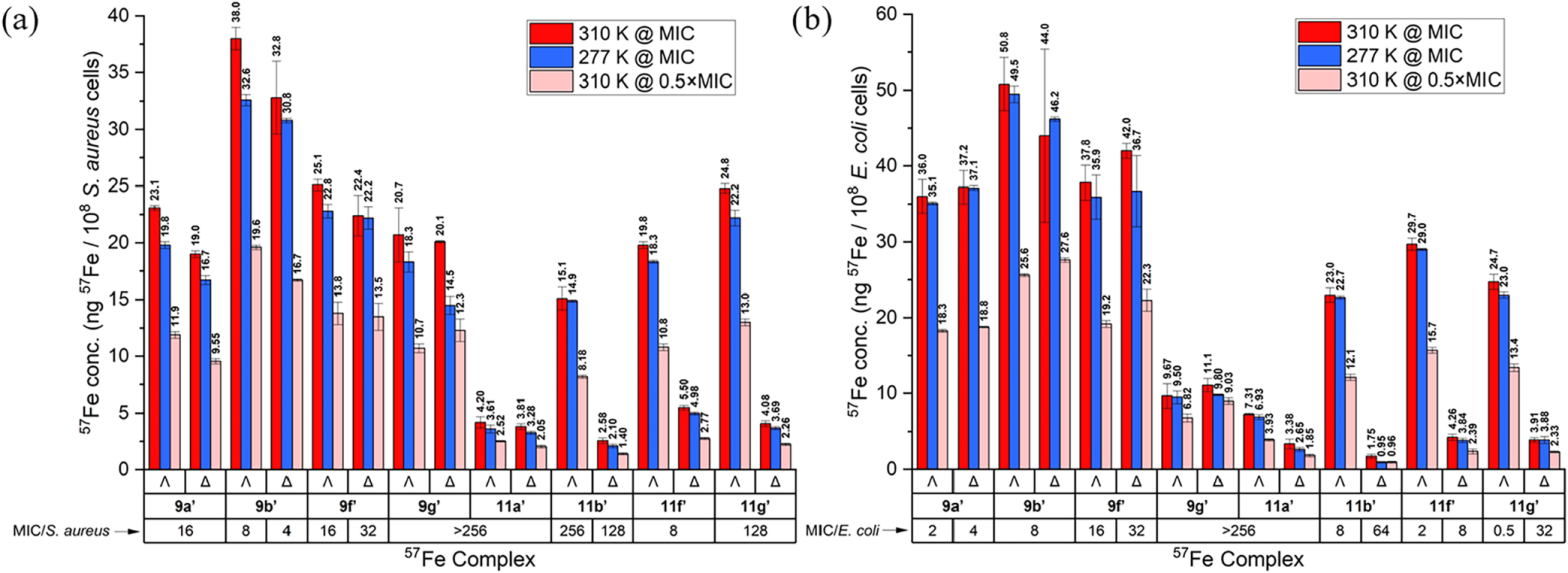
Iron (^57^Fe) cellular accumulation in (a) *S. aureus* USA300 and (b) *E. coli* TOP10 bacteria (ng ^57^Fe/10^8^ cells) when incubated for 30 min with select ^57^Fe-labeled compounds at MIC* and 310 K (red bars), MIC and 277 K (blue), 0.5 × MIC and 310 K (pink). MIC values are reported in μg mL^–1^. *For compounds with MIC values >256 μg ml^–1^, a dosage of 256 μg mL^–1^ was used.

For the ^57^Fe isotopologues of **9** in *S. aureus* at their MIC dosages [Figure 4a, red bars] we saw a narrow range of accumulation (19.3–38.0 ng ^57^Fe per 10^8^ cells), consistent with similar concentrations of each compound being required in the cell to achieve inhibition, i.e., the MIC being principally dependent on the ability of the compound to achieve this accumulation. Enantiomer pairs have very similar levels of accumulation to one another at the MIC.

For the class **9′** in *E. coli* [Figure 4b], we saw similar trends in the data. Note that for **9g′** the MIC was not reached in the experiments of Table 1, and so these compounds were dosed here at 256 μg mL^–1^. We thus propose, as suggested by the zeta-potential measurements above, that as we increase the hydrophobicity of these compounds, the MIC increases because of their inability to enter the microbial cells.

For the triplex series at their respective MICs [Figure 4a,b, red bars], we saw similarly that the inactive compounds **11a′** have low accumulation in both strains at 256 μg mL^–1^, remembering that inhibition is not achieved at this concentration. Also, in both strains, the cellular concentrations of the enantiomers Δ-**11b′**-**g′** at the applied MIC are low, which implies either that the Δenantiomers act very efficiently on an intracellular target, albeit at a high applied dose, or more likely that they have an extracellular mechanism requiring this high dose (vide infra). The Λ configured triplex compounds have very different behavior, and while (in either microbe) the concentrations required in the cell to achieve inhibition are similar for Λ-**11b′**-**g′**, the ability of Λ-**11g′** to achieve this critical accumulation (*ca* 25 ng ^57^Fe per 10^8^ cells) in *E. coli* at an applied dose of just 0.5 μg mL^–1^ is striking.

We thus investigated this apparent mechanistic difference via drug synergy assays.

### Flexicate and Triplex Antimicrobials Have Different Mechanistic Envelopes

In combination therapies, carefully selected drugs are coadministered in an appropriate formulation to create a synergistic effect, with the potential to kill highly resistant bacteria and reduce the evolution of resistance. The determination of such synergy is also useful in the context of a mechanistic study since, broadly speaking, it only arises if the mechanisms of the drug actions are different. We therefore set out to elucidate any synergies among the active molecules reported here.

The standard method for expressing synergy is the use of the fractional inhibitory concentration indices (FICI), whereby the response to a combination of two drug compounds or candidates is described by eq 1.^49,50^ A is the MIC of compound A when in combination, and MIC_*A*_ is the individual MIC of compound A, etc. Responses are defined as synergistic when the FICI is ≤ 0.5, additive when 0.5 < FICI ≤ 1, indifferent when 1 < FICI ≤ 4, and antagonistic when the FICI is >4.^51^ We note, however, that indifference is often difficult to differentiate from an additive effect.

1 $$\mathrm{FICI}={\mathrm{FIC}}_{A}+{\mathrm{FIC}}_{B}=\frac{A}{{\mathrm{MIC}}_{A}}+\frac{B}{{\mathrm{MIC}}_{B}}$$

Checkerboard assays^52^ between the new compound classes were performed against *E. coli* ATCC 25922, as shown in Table 5.

**Table 5:** Calculated FIC*I*_min_ and FIC*I*_max_ Values from In Vitro Metallohelix/Metallohelix Synergy Assays against Bacterial Strain *E. coli* ATCC 25922

						% wells on growth/no growth interface displaying			
#	combination	A	B	FIC*I*_min_	FIC*I*_max_	synergy	additivity	indifference	antagonism
1	flexicate/flexicate	Δ-**9a**	Λ-**9a**	0.52	0.75	0	100	0	0
2		Λ-**9a**	Λ-**9b**	1.06	1.50	0	0	100	0
3		Δ-**9b**	Λ-**9b**	0.38	0.63	25	75	0	0
4	triplex/triplex	Δ-**11b**	Λ-**11b**	0.31	1.02	33	44	22	0
5		Δ-**11f**	Λ-**11f**	0.38	1.07	29	57	14	0
6		Λ-**11b**	Λ-**11f**	0.56	1.13	0	50	50	0
7	flexicate/triplex	Λ-**9a**	Λ-**11b**	0.52	2.25	0	60	40	0
8		Λ-**9a**	Λ-**11f**	0.50	1.13	17	33	50	0
9		Λ-**9a**	Δ-**11f**	0.53	4.25	0	50	20	30
10		Λ-**9a**	Λ-**11g**	0.27	1.02	28	33	39	0

We consider the combinations of compounds in series **9**. With FICI values of 0.5–1, combinations of the parent compound enantiomers **9a** (entry 1) had an additive effect, as confirmed by the proportion of wells in the assay plate displaying this behavior (100%). This is consistent with the group acting via the same mechanisms. Combinations of the parent and propargyl derivative of the same enantiomer emerged as “indifferent” (entry 2), but as we note above, this may be difficult to differentiate from additivity. More importantly, the propargyloxy enantiomers **9b** display significant synergy (entry 3), and the same pair of compounds are also unique in that they display different staining patterns in *E. coli*­(vide infra).

Turning to the triplex compounds, we see (entries 4 and 5) significant synergy between enantiomers in the case of propargyloxy compounds **11b** and also the benzyloxy compounds **11f**, while clearly not (entry 6) between the most active enantiomers, Λ-**11b** and Λ-**11f**. This confirms that the triplex Λ and Δ enantiomers operate by different mechanisms.

For the combinations of the two series, we used the most active compound of the flexicate series, i.e., Λ-**9a**. In entries 7–9, we observe no synergy with the modestly active Λ-**11b** but perhaps a little with Λ-**11f**, while some antagonism is detected with its mirror image Δ-**11f** (see the SI). Most strikingly, however, there is clear synergy between Λ-**9a** and Λ-**11g**, consistent with important mechanistic differences between the two series.

It is reassuring that the increased potency against *E. coli* from the synergistic combinations did not lead to increased erythrocyte toxicity as measured by the same assay as used above (Table S5, SI).

### Metallohelix Location from Click Fluorescence Microscopy

For biosafety reasons, nonpathogenic microbial strains *S. aureus* USA300 and *E. coli* TOP10 were employed in the microscopy studies. They were incubated at their respective MIC concentrations (or at 256 μg mL^–1^ for inactive compounds) with the alkyne derivatives **9b**, Λ-**9g**, Λ-**11b**, and **11g** for 30 min only, and were subsequently labeled with AlexaFluor 488 azide (AF- 488) using CuAAC. Bacterial cells were costained with the fluorescent membrane stain FM4–64 and DNA stain DAPI. Representative images from confocal microscopy for selected compounds plus control images are given in Figures 5 and 6 and S29–S31.

**Figure 5:**
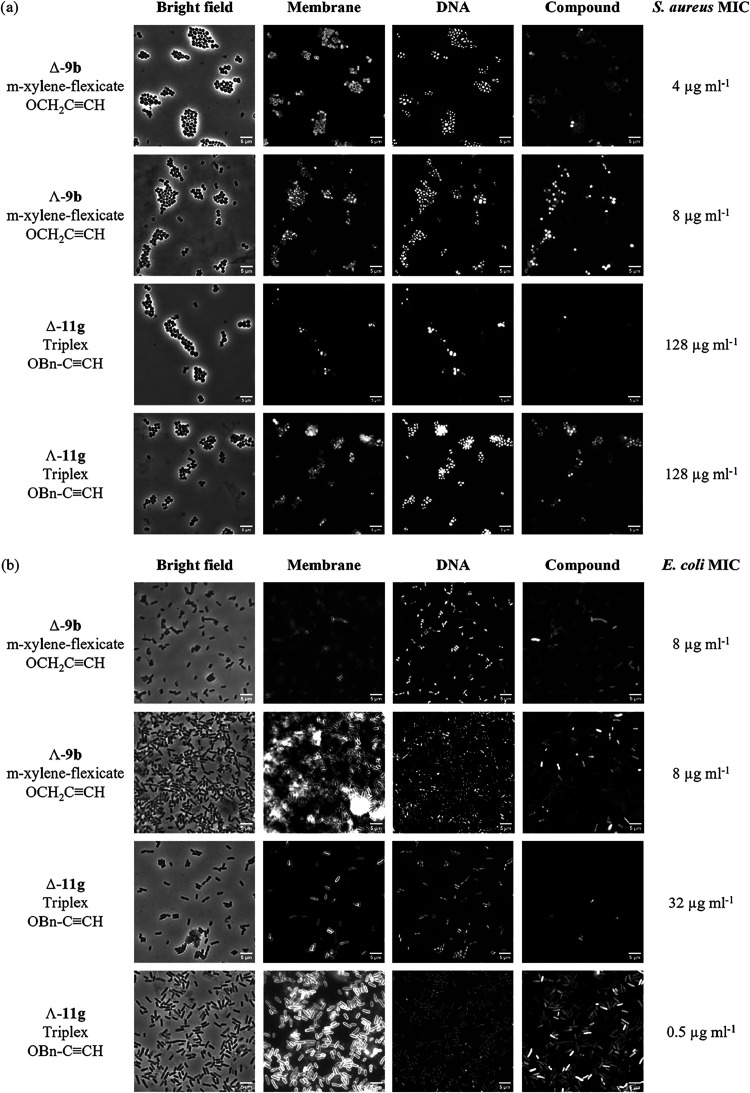
Confocal microscopy images of (a) *S. aureus* USA300 and (b) *E. coli* TOP10 bacteria treated with enantiomers of 9b and 11g at MIC. Shown are the bright field, membrane stain (FM4–64), DNA stain (DAPI), and compound stain (AF-488) images acquired using an LSM510 confocal microscope and Leica X software. Scale bars = 5 μm.

**Figure 6:**
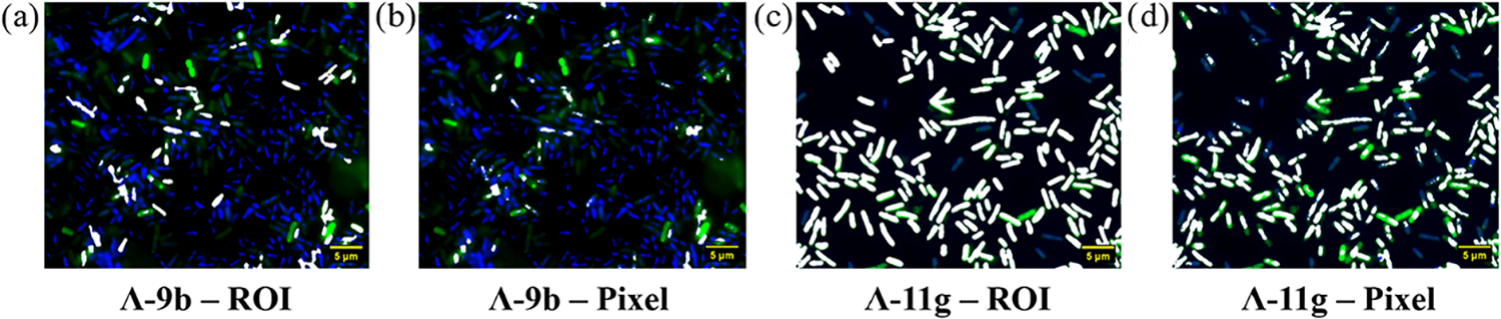
Generated colocalization-per-ROI and colocalization-per-pixel images of *E. coli* TOP10 bacteria treated with compounds at MIC, using images acquired during microscopy; (a+b) Λ-9b; (c+d) Λ-11g. Colocalization (white) was determined between DNA stain (DAPI, blue) and compound stain (AF-488, green) with images attained using ImageJ software. Scale bars = 5 μm.

The membranes of both *E. coli* and *S. aureus* cells treated with the Λ enantiomers of either class of metallohelix are stained across the bacterial population by the dye FM4–64. On treatment with the Δ-metallohelices tested, however, membrane staining is structure and microbe-dependent; when *S. aureus* cells are incubated with flexicate Δ-**9b** [MIC 4 μg/mL] or triplex Δ-**11g** [128 μg/mL], FM4–64 fails to stain some of the cells (and the same individual cells also have no DNA stain), while for *E. coli*, the membrane stain noticeably varies in intensity across the cell population following treatment with the same compounds Δ-**9b** [MIC 8 μg/mL] or Δ-**11g** [32 μg/mL]. We therefore propose that these Δ-metallohelices of both classes are acting on the bacteria at the membrane at these generally higher concentrations, affecting the integration of FM4–64 into the lipid bilayers.

Differences in compound Click staining are also observed. Considering first the flexicates, when Λ-**9b** and Λ-**9g** are incubated at the MIC in *S. aureus*, compound staining is observed heterogeneously across the cell population (Figures 5 and S29, SI), although a greater concentration of Λ-**9g** [MIC > 256 μg/mL] is required to observe this effect. In *E. coli*, while Λ-**9g** is not detected in the cells by these means, Λ-**9b**, which achieves a much higher average concentration (Figure 4b) is observed heterogeneously across the cell population—we have previously noted that compound staining is not observed when quiescent *E. coli* EHEC Sakai cells are treated with Λ-**9b**, and instead only enters exponentially growing cells.^22^ We also once more observed that Λ-**9b** accumulates at the poles of some of the *E. coli* bacteria (Figure S30a, SI), whereas this is not the case with its mirror image Δ-**9b** (Figure S30b, SI), despite sufficient compound entering the bacteria (Figure 4b). We also observe evidence of competitive DNA binding in *E. coli* for the flexicates; in cells treated with Δ- or Λ-**9b**, the cells with compound stain have reduced DAPI staining (Figure S31, SI).

The triplexes behave very differently, particularly in *E. coli* bacteria. No compound staining was observed for Λ-**11b** (Figure S29, SI) in either *S. aureus* or *E. coli* (despite several attempts and the substantial ^57^Fe accumulation measured by ICP-MS) and given that it is unlikely that Λ-**11b** somehow uniquely prevents cellular entry of the azido dye to permeabilized cells, and that it is readily stained in cell-free solution and has efficient and extensive synthetic CuAAC chemistry,^25,33,53^ we suggest that it may be bound strongly at intracellular sites such that it is sterically protected from CuAAC chemistry. No compound staining is observed for triplex Δ-**11g** in either *S. aureus* or *E. coli* (Figure 5), and in parallel with minimal ^57^Fe accumulation shown in Figure 4, this suggests that it struggles to cross the bacterial membranes. In contrast, Λ-**11g** is observed heterogeneously across the *S. aureus* population (as for Λ-flexicates, Figure 5a), albeit at a high concentration. Astonishingly, however, this most active compound, Λ-**11g**, is observed in almost all of the *E. coli* bacteria (Figures 5b and S31, SI) regardless of apparent growth phase, and despite the very low applied concentration (0.5 μg mL^–1^ or *ca* 250 nM). These dramatic differences between triplex enantiomers correlate with ICP-MS results, whereby ^57^Fe accumulation is ca six times higher for Λ-**11g** than for Δ-**11g**, despite a 64-fold lower incubation concentration.

For these key compounds, Λ-**9b** and Λ-**11g**, Figure 6 shows colocalization analyses, in which we created regions of interest (ROI) per *E. coli* bacterium and coded white any cells containing both compound and DAPI signal. This clearly shows the heterogeneous cell uptake of Λ-**9b** (Figure 6a), where many cells have DNA stain only (blue), several have both DNA and compound stain (white), and a few only have just compound stain (green). A further pixel-by-pixel colocalization analysis was performed and leads to a similar output (Figure 6b), and also shows again that this compound tends to accumulate in the poles of cells. In stark contrast, almost all cells treated with Λ-**11g** show DNA and compound stain (Figure 6c), with the pixel-by-pixel colocalization for this compound showing that although both DNA staining and compound staining are observed for the same *E. coli* cell, Λ-**11g** does not only accumulate in the DNA-containing nucleoids but instead can reside in the surrounding cytoplasm (Figure 6d).

## Conclusions

The new compounds of Scheme 1 were synthesized as single enantiomers directly by self-assembly. While the various substituents had no measurable effect on the structure of the cationic metallohelix units of any series, we observed binding between H-bond donors and counteranions by X-ray crystallography. This provides a mechanism for those compounds containing –OCH_2_R (*R* = aryl, alkynyl) units to modify their net charge in response to the environment, i.e., chameleonic behavior.^40,41^

ICP-MS studies, including temperature and concentration dependence, using 16 isotopically labeled compounds in both *S. aureus* and *E. coli*, showed that intracellular transport occurs principally via passive (equilibrative) diffusion. For a given core metallohelix structure, the measured antimicrobial activity closely correlates with the ability of the compound to accumulate in the cell, with active compounds achieving a consistent range of intracellular concentration at their MICs. This relationship is most notable among the triplexes, where enantiomer-specific differences in activity and uptake are very apparent. Six alkynyl derivatives—which conveniently include some of the most active and interesting compounds—were also studied by confocal microscopy using click-based fluorescent labeling and, combined with ICP-MS and checkerboard synergy studies, gave the important insights into mechanism and localization included below.

In the flexicate series **9**, addition of most substituents reduced antimicrobial activity, the exceptions being propargyloxy compounds **9b**—capable of the anion binding mechanism—which had higher accumulation and slightly improved activity in *S. aureus*. The observation that similar accumulations of series **9** compounds are required to effect inhibition, almost regardless of the substituent, alongside additivity behavior in checkerboard assays, suggests that the flexicate compounds with the same core structure largely operate via common intracellular mechanisms.

While enantiomers of the core structure **9a** can pass through *E. coli* membranes efficiently, increasing the hydrophobicity (such as the addition of six benzyl groups in **9f**) caused a reduction in activity and intracellular accumulation, and correlated with weaker interactions of **9f** with model membranes, as measured by zeta-potential titrations: something which would be further accentuated by the anion binding mechanism of Figure 2. Only water-soluble flexicate compounds (**9a, 9b, 9f, and 10a**) had significant antimicrobial activity. We do note however that the click fluorescence microscopy study on this type of compound indicates heterogeneous accumulation across the population; compound Λ-**9a** enters dividing cells more efficiently, probably because the membrane undergoes changes in composition and fluidity at the external negative curvature of the septum.^38,54^ Reduced DAPI staining of *E. coli* cells containing flexicate compounds suggests DNA binding of flexicates, while enantiomers of **9b** seemingly act with different mechanisms of action, evidenced by intracellular staining pattern differences.

In contrast with the parent flexicate compounds of **9** and **10**, the parent triplex enantiomers **11a** are inactive, and ICP-MS studies confirm that they have poor intracellular accumulation. We propose that this is because they are too hydrophilic; they are very soluble in water and have a much smaller hydrophobic region than those of **9a** and **10a**. Correspondingly, the addition of hydrophobic substituents greatly improves transport and activity, particularly against *E. coli*. The anion binding mechanism will serve to further improve or modulate this. Again, in contrast to the flexicate series, the triplex enantiomers behave very differently to one another, and remarkably, while Λ-**11g** has MIC of *ca* 250 nM versus the *E. coli* strain used in the microscopy, its mirror image Δ-**11g** is 64 times less active. Confocal microscopy and ICP-MS studies both revealed that Λ-**11g** enters almost all *E. coli* cells even at very low concentrations, while Δ-**11g** shows minimal intracellular accumulation. Correspondingly, checkerboard assays show synergistic effects between triplex enantiomers and indicate distinct modes of action.

It seems clear that triplex enantiomers operate by largely different mechanisms to one another. For the Λ compounds, it seems very likely that there is a chiral intracellular binding site where the molecule accumulates and/or affects inhibition. Recently, we noted that Λ-**11a** (inactive against all microbes tested) causes cell cycle checkpoint failure in HCT116 colon cancer cells as a result of a quite specific and enantiomer-dependent mode of binding in the cytoskeleton and/or genomic DNA. We might thus postulate, particularly given the dramatic difference in accumulation between triplex enantiomers in *E. coli*, that Λ compounds (such as highly active Λ**-11g**) target bacterial cytoskeleton or nucleic acids; this warrants detailed mechanistic study.

A subtle intracellular mechanism involving intact Λ-metallohelices is also supported by their kinetic stability even in the presence of the Fe chelator deferoxamine, as well as the observation that different compounds and enantiomers exhibit distinct intracellular localization (e.g., Figures 5 and S29–S31, SI). Furthermore, there is an absence of the phenotypic hallmarks associated with, e.g., ROS-mediated Fenton chemistry or surfactant-like activity, such as diffuse macromolecular damage, membrane rupture, or cell swelling and fragmentation.

Further, the structures of Scheme 1 behave differently on addition of hydrophobic (and anion binding) units: the 3-fold symmetric and end-to-end amphipathic series **9** is tuned out of achieving effective intracellular transport, while series **11**, with its antiparallel head-to-head-to-tail (HHT) configuration and resultant (antimicrobial peptide-like) facially amphipathic architecture, provides a lead compound in the enantiomer Λ-**11g**.

Finally, given the high activity of Λ-**11g**, the promising selectivity versus equine red blood cells and ARPE-19 cells, and the apparent ability to freely enter cells across the microbial population (not just cells in the exponential growth phase), we believe that these systems are a promising and underexplored chemical space for antimicrobial discovery.

## Experimental Section

### General Considerations—Synthesis

All solvents and chemicals purchased from commercial sources were used without further purification. Deuterated solvents were purchased from Cambridge Isotope Laboratories or Sigma-Aldrich. ^57^FeCl_2_ (96% purity) was purchased from CK Isotopes Ltd. Air-sensitive chemicals were stored in an MBraun glovebox.

^1^H and ^13^C NMR spectra were recorded on Bruker Avance 300 MHz, Bruker Avance III HD 300 MHz, Bruker Avance III HD 400 MHz, and Bruker Avance III HD 500 MHz spectrometers. NMR assignments were confirmed by ^1^H–^1^H (COSY), ^13^C–^1^H (HSQC), and ^13^C–^1^H (HMBC) correlation experiments where necessary. ^1^H NMR chemical shifts were internally referenced relative to either tetramethylsilane (δH = 0 ppm) or residual proton resonance in the deuterated solvent (e.g., MeOD-*d*_4_ δH = 3.31 ppm, CDCl_3_ δH = 7.26 ppm). Low-resolution ESI-MS spectra were acquired using an ESI-MS Agilent 6130B. Fragments were detected using positive ion mode. High-resolution MS measurements were acquired using a Bruker UHR-Q-TOF MaXis, using positive ion mode. All MS samples were prepared in an acetonitrile or 4:1 methanol/water mix. FTIR spectra were acquired on a JASCO FT/IR-4200 ATR. Data were collected by using OPUS 7.0 software. UV–vis spectra were obtained by using a Jasco V-660 spectrophotometer. Data were collected using 1 cm path-length quartz cuvettes at 298 K, with the following parameters used as standard: bandwidth 1 nm, response time 1 s, wavelength scan range 800–200 nm, scanning speed 200 nm min^‑1^, and data interval 0.2 nm. Jasco Spectra Manager Suite and Origin 2022b were used to analyze the acquired data. Elemental analysis of ligands and complexes was performed by MEDAC Ltd., Chobham, Surrey, U.K., using a Thermo Scientific FlashSmart CHN Elemental Analyzer to detect carbon (C), hydrogen (H), and nitrogen (N) content.

### General Synthesis of Metallohelices

For series **9** (Scheme 1), the diamine (3.0 equiv) and corresponding pyridinecarboxaldehyde (6.0 equiv) were dissolved in methanol (25 mL) and stirred for 24 h at ambient temperature to form a yellow solution. Anhydrous ^56/57^Fe­(II) chloride (2.0 equiv) was added, and an instantaneous color change to deep purple was observed. For series **11**, the bipyridine-amine (3.0 equiv), the corresponding pyridinecarboxaldehyde (3.0 equiv), and anhydrous ^56/57^Fe­(II) chloride (2.0 equiv) were used. The solution was then heated at reflux (80 °C) for 48 h and then concentrated under reduced pressure. The crude material was dissolved in minimum methanol (∼2 mL) and then pipetted into ethyl acetate (75 mL). The precipitate was filtered on fine filter paper, washed with ethyl acetate (3 × 25 mL), and dissolved in methanol. The solvent was removed under reduced pressure to give the desired product as a dark purple solid, which was dried overnight at 50 °C in vacuo. Isolated yields were typically 75–90%. Compounds were characterized by NMR, HRMS, FTIR, and elemental analysis.

### Stability in Aqueous and Biological Media

Metallohelices were prepared to a concentration of 0.03 mM in PBS, water/HCl acid buffer (pH 1.5), or CAMHB. Solutions were sealed in 1 cm path-length plastic macrocuvettes and wrapped with parafilm to reduce evaporation. Initial UV–vis photoabsorption spectra were recorded immediately after solution preparation, following which the cuvettes were incubated at 310 K, and absorbances at appropriate wavelengths and time intervals were recorded. Intermittent spectra were taken after 1, 4, 7, 14, and 28 days of incubation for PBS and CAMHB compounds, and absorbances at defined wavelengths were recorded every 5 min for 12 h for compounds in acid buffer. UV–Vis spectra of PBS, water/HCl buffer, and CAMHB were also recorded in conjunction with the compound solutions and used as baseline measurements when appropriate.

### Stability in the Presence of Deferoxamine (DFO)

Aqueous DFO was added to aqueous solutions of 10 μM metallohelix to a DFO concentration of 20 and 100 μM, and the UV–vis spectra were recorded for 5 d. Equimolar solutions (1 mM) of DFO:FeCl_2_ and DFO:FeCl_3_ were prepared as controls.

### Preparation of Vesicles

Lipid films were formulated by dissolving the chosen lipids (20 mg total, see below) in chloroform:methanol (2:1, v:v, 1.5 mL) and removing the solvent using a rotary evaporator (bath at 20 °C, 1 h) to deposit a thin film on the wall of a round-bottomed flask. The films were hydrated with sodium phosphate buffer (6.7 mL, 25 mM, pH 7.4), to give stock solutions (3 mg mL^‑1^ lipid). To form unilamellar vesicles, the aqueous lipid suspensions were subjected to four freeze/thaw/sonication cycles: sonication of the sample (4 × 30 s); freezing the sample at 20 °C; thawing the sample at room temperature. The following phospholipids were used for each membrane-mimetic system: *E. coli*, POPE: 13.40 mg (67.0 wt %), POPG: 4.64 mg (23.2 wt %), CL: 1.96 mg (9.8 wt %); *S. aureus*, POPG: 11.60 mg (58.0 wt %), CL: 8.40 mg (42.0 wt %).

### General Considerations for Microbiology

All procedures were performed by using sterile techniques. Preceding the experimental work, surfaces were washed thoroughly with 80% ethanol. Where necessary, equipment was purchased sterile or thoroughly sterilized, and reagents were autoclaved prior to use. Cation-adjusted Müller–Hinton broth (CAMHB) and Roswell Park Memorial Institute (RPMI) 1640 media were purchased from Sigma-Aldrich, with CAMHB catalogued as Mueller Hinton Broth 2. Sterile growth media, agar plates, buffers, PBS, and double-distilled water were prepared by the media preparation service of the University of Warwick’s School of Life Sciences, unless stated otherwise. Unless stated, incubation periods were 24 h and without agitation using a Memmert INB200 incubator oven or an Eppendorf New Brunswick S41i CO_2_ incubator shaker. Optical densities at 600 nm (OD600) of bacterial cultures in broth were measured by using Jenway 6300 benchtop spectrophotometers. Centrifugations were performed using a Heraeus Sepatech Biofuge 13 3637 benchtop centrifuge. For all assays, appropriate positive and negative controls were incorporated, with at least two replicates performed for each measurement.

### Bacterial MIC Determination

Following CLSI guidelines, a 3.2 mg mL^–1^ stock solution of each compound was prepared in water or DMSO/water mix (1:10), corresponding to a 12.5-fold concentration of the highest concentration tested, 256 μg mL^–1^. In a sterile 96-well plate, 32 μL of each 3.2 mg mL^–1^ stock was added to 168 μL of antibiotic-free CAMHB to return 512 μg mL^–1^ solutions. These solutions were subject to a 2-fold serial dilution in CAMHB. Overnight cultures of each bacterial strain in CAMHB were diluted in the same medium to a cell concentration of 1 × 10^6^ CFU mL^–1^, before 100 μL addition of this culture to each compound well, allowing a 0.008–256 μg mL^–1^ compound concentration range to be tested. Plates were incubated at 37 °C for 20 h without shaking, and the lowest concentration deemed to inhibit >99% of bacterial growth for each compound was judged to be the MIC. Positive (culture only, no antimicrobial) and negative controls (CAMHB only) were used to ensure suitable bacterial growth and no contamination of media, respectively. Appropriate clinical antimicrobials (ampicillin, tetracycline, ticarcillin, etc.) were used as quality controls and compared to literature values to validate MIC values. Results were repeated in triplicate.

### Hemolysis Assays

Fresh equine blood was centrifuged (1000*g*, 10 min), and the supernatant was removed. Harvested erythrocytes were washed three times with PBS and then resuspended to a 5% erythrocyte concentration in PBS. Metallohelices were dissolved in PBS to form 3.2 mg mL^–1^ stock solutions. These stock solutions were used to prepare 1–1024 μg mL^–1^ serial dilution ranges in 96-well round-bottom plates using PBS. The suspended erythrocytes (100 μL) were added to the metalohelix solution wells (100 μL) and incubated without agitation (310 K, 1 h). The hemolytic concentration for each compound was determined for each compound by visual inspection of wells after the incubation period, in which the lowest concentration deemed to cause >10% cell lysis for each compound was judged to be the hemolytic concentration. Controls included PBS and 1% Triton X-100 as 0 and 100% hemolysis, respectively. Each measurement was performed in triplicate.

### Chemosensitivity (MTT Assay)

ARPE-19 cells were incubated in 96-well plates at a cell concentration of 0.5 × 10^4^ cells mL^–1^. The cells were used when between 50 and 80% confluent in the stock flasks. Complete cell media containing DMEM, supplemented with 10% fetal calf serum and l-glutamine (2 mM), was used to prepare the desired cell concentration and reference wells. Plates containing cells were incubated for 24 h at 37 °C in 5% CO_2_ atm, prior to drug exposure. Cell media (200 μL) was added to the reference cells, and differing concentrations of drug solution (200 μL) were added to the remaining wells. The plates were incubated for a further 96 h at 37 °C in 5% CO_2_ atmosphere. 3-(4,5-Dimethylthiazol-1-yl)-2,5-diphenyltetrazolium bromide (MTT) solution (0.5 mg mL^–1^, 20 μL per well) was added to each well and incubated for 4 h at 37 °C in 5% CO_2_ atm. Upon completion, all solutions were removed from the wells and DMSO (150 μL) was added to each well to dissolve the purple formazan crystals. A Thermo Scientific Multiskan EX microplate photometer was used to measure the absorbance at 540 nm. Lanes containing 100% cell media and untreated cells were used as a blank and 100% cell survival, respectively. Cell survival was determined as the absorbance of treated cells minus the blank cell media, divided by the absorbance of the untreated control; this value was expressed as a percentage. The IC_50_ values were determined from a plot of percentage cell survival against drug concentration (μM). Assays were conducted in triplicate, and the mean IC_50_ ± standard deviation was determined.

### Synergy Assays

Compounds were prepared in water at 4× the highest desired concentration to test. 50 μL of CAMHB was added into each well of a sterile 96-well plate. 100 μL of the first compound of interest was added and serially diluted along the plate ordinate, followed by addition of 50 μL of the second compound and serial dilution along the abscissa. Overnight CAMHB cultures of *E. coli* (strain ATCC 25922) for metallohelix/metallohelix assays or *S. aureus* (strain USA300) for metallohelix/cefoxitin assays were diluted in the same medium to a cell concentration of 1 × 10^6^ CFU ml^–1^, before 100 μL addition of this culture to each compound well. The final checkerboard thus contains combinations of the two antibiotics, with the highest concentration of each antibiotic at opposite well corners and an MIC row and column for each of the two compounds individually. Plates were incubated at 37 °C for 20 h without shaking, and the plates were inspected for bacterial growth inhibition.

### ICP-MS Determination of Cellular Accumulation

Overnight cultures of *S. aureus* USA300 and *E. coli* TOP10 were grown in CAMHB (310 K) to the exponential phase (OD600 ≈ 0.5). Once at the exponential phase, each compound was added to the culture (1 mL) to afford the desired compound concentration (8 μg mL^–1^, MIC or 0.5 × MIC), and samples were incubated with shaking (277 or 310 K, 30 min). For samples incubated at 277 K, cultures were prechilled before dosage (277 K, 15 min). Samples were then pelleted by centrifugation (8000*g*, 5 min), the supernatant was removed, and a 2-fold repeat of resuspension and pelleting was performed. The *S. aureus* pellets were treated with 4% paraformaldehyde and incubated with shaking (277 K, 15 min) before pelleting and the removal of the supernatant. Following resuspension of all pellets in PBS, samples were subject to a final centrifugation and removal of supernatant, and the resultant pellets were frozen (253 K) until digestion.

68% v/v nitric acid (300 μL) was used to digest bacteria pellets before the suspensions were heated overnight at 348 K to ensure complete digestion. Each sample was diluted 20-fold using 18.2 MΩ.cm Milli-Q water to attain concentrations of 3.4% v/v nitric acid (total dissolved solids <0.2% w/v). ^57^Fe calibration solutions were prepared in the range 0.1–1000 ppb using 10 ppm (10 μg mL^‑1^) ^57^Fe plasma standard solution (Thermo Fisher Scientific) using 3.4% v/v nitric acid. ^57^Fe accumulation was recorded using an Agilent 7900 ICP-MS spectrometer running in the He gas mode.

### Click Fluorescence Confocal Microscopy

An overnight culture of *E. coli* TOP10 or *S. aureus* USA300 was diluted in CAMHB and grown to midexponential (OD600 ≈ 0.50) before dosage with metallohelix to the MIC (or 256 μg mL^‑1^ if the MIC value for the compound was >256 μg mL^‑1^) or methanol (negative control) and incubated with agitation (310 K, 30 min). During incubation, the Click-iT cell reaction mix was prepared from the Click-iT Cell Reaction Buffer Kit according to the manufacturer’s instructions (880 μL Click-iT cell reaction buffer, 20 μL CuSO4 solution, 100 μL Click-iT cell buffer additive, 5 μL AF-488 azide). 10 min before the end of the incubation period, 5 μg mL^‑1^ of FM4–64 was added to stain the cell membrane, and the cells (1 mL for each treatment) were collected by centrifugation and fixed with 4% paraformaldehyde (277 K, 15 min). The cells were subsequently washed with PBS, the supernatant was removed, and the pellet was permeabilized with 0.5% Triton X-100 in PBS by incubation with agitation (298 K, 30 min) to enhance AF-488 translocation across cellular membranes. Cells were washed in PBS and in 2% bovine serum albumin (BSA) and then resuspended in the prepared click reaction mix (180 μL, contains 5 μg mL^–1^ AF-488 azide). Solutions were incubated in the dark (298 K, 30 min), washed with 2% BSA in PBS, and stained with 1 μg mL^–1^ DAPI for 1 min. Finally, the cells were washed with PBS, resuspended in PBS, and mounted on slides for microscopy. The slides were prepared using agarose pads prepared with Thermo Scientific Gene Frame Seals, to which ∼3 μL of sample was added and allowed to dry, followed by addition of 4 μL of SlowFade Gold Antifade reagent (Thermo Scientific) to protect fading of the fluorescent dyes during microscopy use. Two independent experiments were performed using each metallohelix. Images were obtained using an LSM510 confocal microscope with Leica X software and analyzed with ImageJ and Fiji software. Images were converted to 16-bit grayscale to facilitate visual interpretation. Image spatial resolution: 60 nm/pixel.

## Supplementary Material



## Notes

Synthetic methods of all compounds, both NMR and high-resolution mass spectra of metallohelices, kinetic stability studies, model vesicle preparation and ζ-potential measurement graphs, microbiology methods, and results (assays include MIC and MBC determination, hemolysis, chemosensitivity, antimicrobial stability, synergy, FICI hemolysis, and ICP-MS and fluorescence confocal microscopy) (PDF)

## #

J.M. Deceased on March 08, 2023.
