# Supplementary material for: Optimizing Intracellular Transport of Antimicrobial Metallohelices Delivers Selective Nanomolar Potency in E. coli
Source: Inorg Chem. 2026 Jan 3;65(2):1513–27. doi: 10.1021/acs.inorgchem.5c05039 (PMC12820927; doi:10.1021/acs.inorgchem.5c05039)
Supplement: Supplementary file 1 [file ic5c05039_si_001.pdf]

Supporting Information

Optimizing Intracellular Transport of Antimicrobial Metallohelices Delivers Selective Nanomolar Potency in *E. coli*

Miles L. Postings,[a] Nicola J. Rogers,[b] Georgia Shreeve,[a] Hualong Song,[c] Guy Clarkson,[a] Anish Mistry,[a] John Moat,[d] Grace Taylor-Joyce,[e] Nicholas R. Waterfield,[e] Peter Scott.[a]

[a] Department of Chemistry, University of Warwick, Gibbet Hill Road, Coventry, CV4 7AL, UK

email: peter.scott@warwick.ac.uk

[b] Department of Chemistry, Hong Kong Baptist University, Kowloon Tong, Hong Kong

email: nicolajrogers@googlemail.com

[c] Beijing Area Major Laboratory of Peptide and Small Molecular Drugs, Engineering Research Centre of Endogenous Prophylactic of Ministry of Education of China, School of Pharmaceutical Sciences, Capital Medical University, Beijing, 100069, China

[d] School of Life Sciences, Gibbet Hill Road, University of Warwick, Coventry, CV4 7AL, UK

[e] Warwick Medical School, University of Warwick, Coventry, CV4 7AL, UK

Table of Contents

1. Synthesis 3

1.1 General Considerations 3

1.2 Synthesis and characterisation of small organic molecules 3

1.3 Synthesis and characterisation of chiral diamines 9

1.4 Synthesis and characterisation of chiral amines 11

1.5 Synthesis and characterisation of $[^{56/57}Fe_2L_3]Cl_4$ flexicates 12

1.6 Synthesis and characterisation of $[^{56/57}Fe_2L_3]Cl_4$ triplexes 26

1.7 Synthesis and characterisation of $[Zn_2L_3][ClO_4]_4$ flexicates 34

1.8 Synthesis and characterisation of $[Zn_2L_3][ClO_4]_4$ triplexes 36

2. NMR Spectra 39

2.1 Fe metallohelices 39

2.2 Zn metallohelices 46

3. High resolution ESI mass spectra 48

4. Stability Studies 52

5.1 Aqueous and biological media stability 52

5.2 Deferoxamine (DFO) stability 53

5. Model vesicle studies 54

5.1 Preparation of model vesicles 54

5.2 Zeta Potential Measurement Graphs 54

6. Microbiology methods 55

6.1 General considerations 55

6.2 Bacterial minimum inhibitory concentration (MIC) determination 55

6.3 Bacterial minimum bactericidal concentration (MBC) determination 55

6.4 Haemolysis assays 55

6.5 Chemosensitivity (MTT assay) 56

6.6 Antimicrobial stability of compounds at different storage durations and temperatures 56

6.7 Synergy assays 56

6.8 FICI haemolysis assays 57

6.9 ICP-MS 57

6.10 CuAAC Fluorescence Confocal Microscopy 57

7. Microbiology Results 59

7.1 Bacterial minimum inhibitory concentration (MIC) determination 59

7.2 Bacterial minimum bactericidal concentration (MBC) determination 60

7.3 Haemolysis assays 61

7.4 Antimicrobial stability of compounds at different storage durations and temperatures 61

7.5 Synergy assays 62

7.6 FICI haemolysis assays 63

7.7 ICP-MS 64

7.8 CuAAC Fluorescence Confocal Microscopy 66

8. References 68

1. Synthesis

1.1 General Considerations

All solvents and chemicals purchased from commercial sources (Acros Organics, Alfa Aesar, Fisher Scientific, Fluorochem, Merck/Sigma-Aldrich or VWR) were used without further purification unless otherwise stated. Deuterated solvents were purchased from Cambridge Isotope Laboratories or Sigma-Aldrich. $^{57}FeCl_2$ (96% purity) was purchased from CK Isotopes Ltd. If appropriate, chemicals were stored in an MBraun glove box. If necessary, solvents were freeze-thaw degassed before use, with dried solvents stored in glass ampoules under argon. Sodium hydride dispersions in mineral oil were placed in a Schlenk vessel under an argon atmosphere, washed five times with anhydrous hexane to remove the mineral oil, then dried and stored in an MBraun glove box. Where appropriate, reactions were carried out under argon using a dual manifold argon/vacuum line and standard Schlenk techniques. All glassware and cannulae for these techniques were stored in an oven at 393 K for a minimum of 24 h prior to use.

^{1}H and ^{13}C NMR spectra were recorded on Bruker Avance 300 MHz, Bruker Avance III HD 300 MHz, Bruker Avance III HD 400 MHz and Bruker Avance III HD 500 MHz spectrometers. NMR assignments were confirmed by ^{1}H-^{1}H (COSY), ^{13}C-^{1}H (HSQC) and ^{13}C-^{1}H (HMBC) correlation experiments where necessary. ^{1}H NMR chemical shifts were internally referenced relative to either tetramethylsilane (δ_H = 0 ppm) or residual proton resonance in the deuterated solvent (e.g. MeOD-d$_4$ δ_H = 3.31 ppm, $CDCl_3$ δ_H = 7.26 ppm). Low resolution ESI-MS spectra were acquired using an ESI-MS Agilent 6130B. Fragments were detected using positive ion mode within m/z 50-500, m/z 400-1000 or m/z 800-2000 scan ranges. High resolution MS measurements were acquired by Dr Lijiang Song/Dr Joanna Drozd (University of Warwick) using a Bruker UHR-Q-TOF MaXis, using positive ion mode in the scan range of 50-3000 m/z. All MS samples were prepared in acetonitrile or 4:1 methanol/water mix. FTIR spectra were acquired on a JASCO FT/IR-4200 ATR in the range 4000-400 cm^{-1}. Data were collected using OPUS 7.0 software. UV-Vis spectra were obtained using a Jasco V-660 spectrophotometer. Data were collected using 1 cm path-length quartz cuvettes at 298 K, with the following parameters used as standard: bandwidth 1 nm, response time 1 s, wavelength scan range 800-200 nm, scanning speed 200 nm min^{-1}, data interval 0.2 nm. Jasco Spectra Manager Suite and Origin 2022b were used to analyse acquired data. Elemental analysis of ligands and complexes were performed by MEDAC Ltd, Chobham, Surrey, UK, using a Thermo Scientific Flash*Smart*™ CHN Elemental Analyser to detect carbon (C), hydrogen (H) and nitrogen (N) content. TGA measurements were performed using a DSC1-1600 scanning calorimeter in which an accurately weighed 40 µl aluminium crucible containing compound was heated from 298 to 673 K at 5 K min^{-1} under dinitrogen. Data were collected using STARe software and analysed in Origin 2022b.

(*R*)- and (*S*)-2-phenylglycinol (*R*-**1** and *S*-**1**),[1] 5-hydroxypicolinaldehyde,[2] 5-(prop-2-yn1-yloxy)picolinaldehyde (**8b**)[3] and 5-(chloromethyl)-2,2'-bipyridine (**4**)[4,5] were synthesised following known literature methods and used in the following procedures. Dibromomethylxylenes **2** and **3** were purchased from Sigma-Aldrich. Syntheses of complexes **9a-b**,[6] **10a**,[7] **11a,b,f**[3,8] have been described in previous group publications. Details for the synthesis of some other literature compounds are given below where the method is modified.

1.2 Synthesis and characterisation of small organic molecules

General procedure for the synthesis of compounds 8c-g

5-(Hydroxy)picolinaldehyde (0.40 g, 3.2 mmol, 1 eq.) was dissolved in acetonitrile (30 ml), followed by the addition of potassium carbonate (0.47 g, 3.4 mmol, 1.05 eq.) and the solution was stirred at ambient temperature for 1 h. The appropriate bromide (3.4 mmol, 1.05 eq.) was added, and the solution stirred at reflux (85 °C) for 18 h. After cooling to ambient temperature, the solution was filtered through celite. The solvent was removed under reduced pressure and the residue was purified by silica gel column chromatography (DCM/MeOH, 50:1 v/v) to yield pure products.

5-(Propoxy)picolinaldehyde (8c)

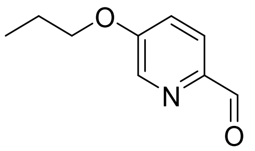

Compound **8c** was synthesised from the general procedure described above, using 1-bromopropane. Column chromatography purification: (DCM/MeOH, 50:1 v/v, R_f = 0.61).

Yield 0.31 g, 1.9 mmol, 59%.

^{1}H NMR (400 MHz, [D$_6$]-DMSO, ppm): δ_H 9.89 (s, 1H, CHO), 8.49 (d, $^4J_{HH}$ = 2.0 Hz, 1H, NCH), 7.93 (d, $^3J_{HH}$ = 8.5 Hz, 1H, NCCH), 7.57 (dd, $^3J_{HH}$ = 8.5 Hz, $^4J_{HH}$ = 2.5 Hz, 1H, NCCHC*H*), 4.14 (t, $^3J_{HH}$ = 6.5 Hz, 2H, OCH_2), 1.85-1.72 (m, 2H, C*H*$_2$CH$_3$), 1.00 (t, $^3J_{HH}$ = 7.5 Hz, 3H, CH_3).

^{13}C{^{1}H} NMR (101 MHz, 298 K, DMSO): δ_C 192.5 (CHO), 158.8 (CO), 146.1 (*C*CHO), 139.3 (NCH), 124.0 (NCCH*C*H), 121.4 (NC*C*H), 70.5 (OCH_2), 22.2 (*C*H$_2$CH$_3$), 10.7 (CH_3).

MS (ESI): m/z 166.1 $[M+H]^+$, 188.0 $[M+Na]^+$.

FTIR: υ cm^{-1} 3450 w (br), 2969 w, 2939 w, 2881 w, 2820 w, 1702 s, 1569 s, 1473 m, 1312 m, 1261 m, 1209 s, 1126 m, 1053 m, 1026 s, 1005 s, 967 m, 844 m, 821 m, 759 m, 656 m.

Elemental analysis found (calculated for $C_9H_{11}NO_2$) % C 65.81 (65.44), H 6.99 (6.71), N 8.68 (8.48).

5-(Pentyloxy)picolinaldehyde (8d)

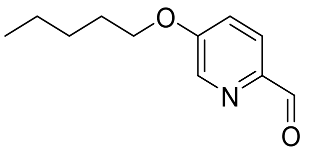

Compound **8d** was synthesised from the general procedure described above, using 1-bromopentane. Column chromatography purification: (DCM/MeOH, 50:1 v/v, R_f = 0.54).

Yield 0.42 g, 2.2 mmol, 67%.

^{1}H NMR (300 MHz, 298 K, DMSO): δ_H 9.88 (s, 1H, CHO), 8.47 (d, $^4J_{HH}$ = 3.0 Hz, 1H, NCH), 7.92 (d, $^3J_{HH}$ = 8.5 Hz, 1H, NCCH), 7.57 (dd, $^3J_{HH}$ = 8.5 Hz, $^4J_{HH}$ = 3.0 Hz, 1H, NCCHC*H*), 4.16 (t, $^3J_{HH}$ = 6.5 Hz, 2H, OCH_2), 1.82-1.69 (m, 2H, OCH$_2$C*H*$_2$), 1.50-1.23 (m, 4H, C*H*$_2$C*H*$_2$CH$_3$), 0.89 (t, $^3J_{HH}$ = 7.0 Hz, 3H, CH_3).

^{13}C{^{1}H} NMR (75 MHz, 298 K, DMSO): δ_C 192.0 (CHO), 158.3 (CO), 145.6 (*C*CHO), 138.8 (NCH), 123.5 (NCCH*C*H), 120.9 (NC*C*H), 68.6 (OCH_2), 28.1 (OCH$_2$*C*H$_2$), 27.5 (*C*H$_2$CH$_2$CH$_3$) 21.8 (*C*H$_2$CH$_3$), 13.8 (CH_3).

MS (ESI): m/z 194.1 $[M+H]^+$.

FTIR: υ cm^{-1} 2954 m, 2932 m, 2860 w, 2814 w, 1702 s, 1571 s, 1468 m, 1311 s, 1260 s, 1208 s, 1124 m, 1011 m, 844 m, 607 m.

Elemental analysis found (calculated for $C_{11}H_{15}NO_2$) % C 68.51 (68.37), H 7.88 (7.82), N 7.22 (7.25).

2-(2-Bromoethoxy)ethan-1-ol[9]

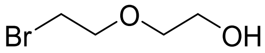

Diethylene glycol (5.00 g, 47.1 mmol, 1 eq.) and 48% w/w aq. HBr (5.4 ml, 3.62 g, 47.1 mmol) were added to toluene (125 ml) and the mixture was stirred at reflux (110 °C) using a Dean-Stark apparatus for 12 h. The reaction mixture was allowed to cool to ambient temperature then the reaction was quenched with 1 M sodium hydroxide at 0 °C (50 ml). The organic layer was diluted with ethyl acetate, washed with water and brine, and then dried over sodium sulfate. The solvent was removed under reduced pressure to leave a yellow oil. The product was obtained by silica gel column chromatography (hexane/ethyl acetate, 3:1, R_f = 0.42) as a yellow oil.

Yield: 1.04 g, 6.15 mmol, 13%.

^{1}H NMR (400 MHz, 298 K, $CDCl_3$): δ_H 3.83 (t, $^{3}J_{HH}$ = 5.5 Hz, 2H, C$H_2$$CH_2$Br), 3.77 (m, 2H, C$H_2$OH), 3.64 (t, $^{3}J_{HH}$ = 3.5 Hz, 2H, C$H_2$$CH_2$OH), 3.50 (t, $^{3}J_{HH}$ = 5.5 Hz, 2H, CH_2Br), 2.15 (br s, 1H, OH).

^{13}C{^{1}H} NMR (101 MHz, 298 K, $CDCl_3$): δ_C 72.2 (*C*$H_2$$CH_2$OH), 70.9 (*C*$H_2$$CH_2$Br), 61.7 ($CH_2$OH), 30.6 ($CH_2$Br).

MS (ESI): m/z 193.0 [M+Na]$^+$, 171.0 [M+H]$^+$.

FTIR: υ cm^{-1} 3385 m (br), 2922 w, 2877 w, 1412 m, 1323 m, 1270 m, 1122 s, 1055 m, 892 m, 715 m, 532 m.

5-(2-(2-Hydroxyethoxy)ethoxy)picolinaldehyde (**8e**)

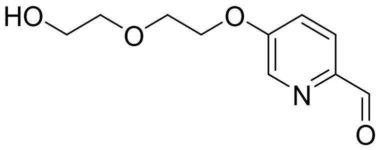

Compound **8e** was synthesised from the general procedure described above, using 2-(2-bromoethoxy)ethan-1-ol. Column chromatography purification: (DCM/MeOH, 50:1 v/v, R_f = 0.53).

Yield 0.34 g, 1.6 mmol, 50%.

^{1}H NMR (400 MHz, 298 K, DMSO): δ_H 9.89 (s, 1H, CHO), 8.51 (d, $^{4}J_{HH}$ = 2.0 Hz, 1H, NCH), 7.94 (d, $^{3}J_{HH}$ = 8.5 Hz, 1H, NCCH), 7.61 (dd, $^{3}J_{HH}$ = 8.5 Hz, $^{4}J_{HH}$ = 2.0 Hz, 1H, NCCHC*H*), 4.63 (br s, 1H, OH), 4.37-4.25 (m, 2H, Py-OCH_2), 3.84-3.74 (m, 2H, CH_2OH), 3.50 (s, 4H, CH_2OCH_2).

^{13}C{^{1}H} NMR (101 MHz, 298 K, DMSO): δ_C 191.9 (CHO), 158.1 (CO), 145.6 (*C*CHO), 138.7 (NCH), 123.4 (NCCHC*H*), 121.0 (NC*C*H), 72.4 (Py-OCH_2), 68.5 (O*C*H_2), 68.2 (O*C*H_2), 60.2 (CH_2OH).

MS (ESI): m/z 234.1 [M+Na]$^+$.

FTIR: υ cm^{-1} 2936 m, 2928 m, 2874 w, 1700 s, 1565 s, 1441 m, 1321 s, 1263 s, 1200 s, 1123 m, 1020 m, 890 m, 844 m, 601 m, 550 m.

Elemental analysis found (calculated for $C_{10}H_{13}NO_4$) % C 56.49 (56.87), H 6.59 (6.20), N 6.40 (6.63).

5-(Benzyloxy)picolinaldehyde (8f)

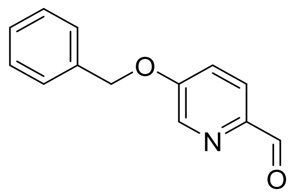

Compound **8f** was synthesised from the general procedure described above, using benzyl bromide. Column chromatography purification: (DCM/MeOH, 50:1 v/v, R_f = 0.45).

Yield 0.42 g, 2.0 mmol, 69%.

^{1}H NMR (400 MHz, 298 K, DMSO): δ_H 9.89 (s, 1H, CHO), 8.57 (d, $^4J_{HH}$ = 2.5 Hz, 1H, NCH), 7.95 (d, $^3J_{HH}$ = 8.5 Hz, 1H, NCCH), 7.67 (dd, $^3J_{HH}$ = 8.5 Hz, $^4J_{HH}$ = 3.0 Hz, 1H, NCCHC*H*), 7.54-7.29 (m, 5H, Ph), 5.32 (s, 2H, CH_2).

$^{13}C\{^1H\}$ NMR (101 MHz, 298 K, DMSO): δ_C 192.0 (CHO), 157.9 (CO), 145.8 (*C*CHO), 139.0 (NCH), 135.8/128.5128.3/128.0 (Ph), 123.5 (NCCHC*H*), 121.5 (NC*C*H), 70.1 (CH_2).

MS (ESI): m/z 236.2 $[M+Na]^+$.

FTIR: ʋ cm^{-1} 3063 w, 3034 w, 2818 w, 1698 s, 1567 s, 1256 m, 1204 s, 1124 s, 1012 m, 788 m, 662 m, 427 m.

Elemental analysis found (calculated for $C_6H_5NO_2$) % C 72.92 (73.23), H 5.23 (5.20), N 6.72 (6.57).

Ethyl 4-trimethylsilylethynylbenzoate[10]

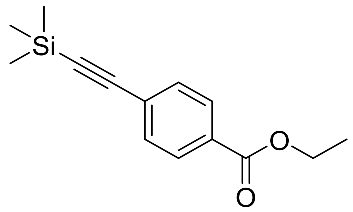

Ethyl-4-iodobenzoate (25.0 g, 90.5 mmol, 1 eq.), tetrakis(triphenylphosphine)palladium (0.09 g, 0.08 mmol, 0.001 eq.) and copper iodide (0.090 g, 0.47 mmol, 0.005 eq.) were added to triethylamine (250 ml) under argon atmosphere and stirred for 15 min. Trimethylsilylacetylene (16.0 ml, 11.4 g, 0.116 mol, 1.3 eq.) was added, the mixture was stirred at ambient temperature for 30 min and then for 12 h at reflux (95 °C). The resultant solution was filtered and concentrated under reduced pressure and the residue was purified by silica-gel chromatography (hexane/ethyl acetate, 4:1, R_f = 0.60) to obtain a yellow solid.

Yield: 14.6 g, 59.3 mmol, 66%.

^{1}H NMR (400 MHz, 298 K, DMSO): δ_H 7.98 (d, $^3J_{HH}$ = 8.0 Hz, 2H, Ph), 7.52 (d, $^3J_{HH}$ = 8.0 Hz, 2H, Ph), 4.38 (q, $^3J_{HH}$ = 7.0 Hz, 2H, CH_2), 1.40 (t, $^3J_{HH}$ = 7.0 Hz, 3H, CH_2CH_3), 0.27 (s, 9H, $SiCH_3$).

$^{13}C\{^1H\}$ NMR (101 MHz, 298 K, $CDCl_3$): δ_C 166.1 (C=O), 132.0/130.2/129.5/127.8 (Ph), 104.3 (SiC≡*C*), 97.7 (Si*C*≡C), 61.3 (CH_2), 14.5 (CH_2CH_3), 0.07 ($SiCH_3$).

MS (ESI): m/z 269.2 $[M+Na]^+$.

FTIR: ʋ cm^{-1} 2960 w, 2904 w, 2160 m, 1717 m, 1231 s, 1202 m, 1031 m, 864 s, 839 s, 814 s, 697 m, 634 m, 533 m.

4-Trimethylsilylethynylphenylmethanol[10]

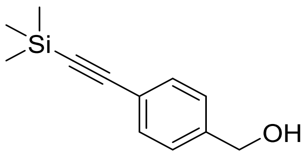

A solution of ethyl 4-trimethylsilylethynylbenzoate (14.6 g, 59.3 mmol, 1 eq.) in anhydrous diethyl ether (100 ml) was added to a suspension of lithium aluminium hydride (2.4 g, 63 mmol, 1.1 eq.) in anhydrous diethyl ether (100 ml) at 0 °C. The mixture was stirred at 0 °C for 4 h before dropwise addition of acetone (20 ml). After filtration, the filtrate was washed with brine (3×100 ml) and the organic layer was dried over $MgSO_4$. The solution was filtered and concentrated under reduced pressure. The residue was purified by silica-gel chromatography (hexane/ethyl acetate, 4:1, R_f = 0.35) to yield a yellow solid.

Yield: 8.78 g, 43.0 mmol, 73%.

^{1}H NMR (400 MHz, 298 K, DMSO): δ_H 7.45 (d, $^3J_{HH}$ = 8.0 Hz, 2H, Ph), 7.28 (d, $^3J_{HH}$ = 8.0 Hz, 2H, Ph), 4.66 (s, 2H, CH_2), 2.13 (s, 1H, OH), 0.25 (s, 9H, $SiCH_3$).

^{13}C{^{1}H} NMR (101 MHz, 298 K, $CDCl_3$): δ_C 141.2/132.2/126.6/122.2 (Ph), 102.3 (SiC≡*C*), 98.2 (Si*C*≡C), 61.1 (CH_2), 0.09 ($SiCH_3$).

MS (ESI): m/z 205.1 $[M+H]^+$.

FTIR: ʋ cm^{-1} 3285 w (br), 2959 w, 2156 m, 1271 s, 1248 m, 1031 m, 860 s, 837 s, 819 s, 757 s, 697 m, 636 m, 586 m, 533 m.

(4-Ethynylphenyl)methanol[10]

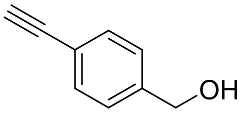

Under argon atmosphere, a solution of 4-trimethylsilylethynylphenylmethanol (5.00 g, 24.5 mmol, 1 eq.) in anhydrous methanol (25 ml) was added dropwise to a solution of potassium carbonate (0.034 g, 0.024 mmol, 0.01 eq.) in anhydrous methanol (25 ml). The mixture was stirred for 18 h at ambient temperature followed by filtration and concentration of the filtrate under reduced pressure. The residue was purified by silica-gel chromatography (hexane/ethyl acetate, 4:1, R_f = 0.30), to produce a yellow oil.

Yield: 1.88 g, 14.2 mmol, 58%.

^{1}H NMR (400 MHz, 298 K, DMSO): δ_H 7.47 (d, $^3J_{HH}$ = 8.0 Hz, 2H, Ph), 7.30 (d, $^3J_{HH}$ = 8.0 Hz, 2H, Ph), 4.67 (d, $^3J_{HH}$ = 5.5 Hz, 2H, CH_2), 3.07 (s, 1H, C≡CH), 2.09 (t, $^3J_{HH}$ = 5.5 Hz, 1H, OH).

^{13}C{^{1}H} NMR (101 MHz, 298 K, $CDCl_3$): δ_C 141.5/132.6/126.1/121.5 (Ph), 85.5 (*C*≡CH), 84.3 (C≡*C*H), 62.1 (CH_2).

MS (ESI): m/z 155.1 $[M+Na]^+$.

FTIR: ʋ cm^{-1} 3281 m (br), 2923 w, 2872 w, 1505 m, 1410 m, 1208 m, 1027 m, 1013 s, 839 m, 815 s, 615 s, 555 m, 516 m, 495 m.

1-(Bromomethyl)-4-ethynylbenzene[10]

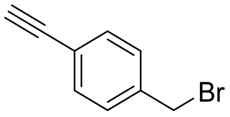

(4-Ethynylphenyl)methanol (1.75 g, 13.2 mmol, 1 eq.) was dissolved in DCM (50 ml) under argon atmosphere and cooled to 0 °C. Carbon tetrabromide (5.84 g, 17.6 mmol, 1.33 eq.) and triphenylphosphine (4.17 g, 15.9 mmol, 1.2 eq.) were added and the mixture was stirred for 1 h at 0 °C. After washing with saturated $NaHCO_3$ solution (3×50 ml), the organic layer was dried over $MgSO_4$. The mixture was filtered and concentrated under reduced pressure, then purified by silica-gel chromatography (hexane/ethyl acetate, 10:1, R_f = 0.55) to give a yellow solid.

Yield: 0.23 g, 1.2 mmol, 8.9%.

^{1}H NMR (400 MHz, 298 K, DMSO): δ_H 7.46 (d, $^3J_{HH}$ = 7.5 Hz, 2H, Ph), 7.35 (d, $^3J_{HH}$ = 7.5 Hz, 2H, Ph), 4.47 (s, 1H, CH_2), 3.10 (s, 1H, C≡CH).

^{13}C{^{1}H} NMR (101 MHz, 298 K, $CDCl_3$): δ_C 143.1/132.5/126.0/121.1 (Ph), 85.9 (*C*≡CH), 83.9 (C≡*C*H), 32.7 (CH_2).

MS (ESI): m/z 197.0 $[M+H]^+$ (^{81}Br), 195.0 $[M+H]^+$ (^{79}Br).

FTIR: υ cm^{-1} 3270 m, 2928 w, 2890 w, 1713 s, 1604 m, 1268 s, 1240 s, 1171 m, 1118 s, 1015 m, 855 m, 814 s, 764 s, 694 m, 639 s, 617 s, 554 m.

5-((4-Ethynylbenzyl)oxy)picolinaldehyde (**8g**)

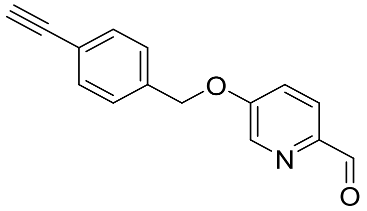

Compound **8g** was synthesised from the general procedure described above on a reduced scale, using 1-(bromomethyl)-4-ethynylbenzene. Column chromatography purification: (DCM/MeOH, 50:1 v/v, R_f = 0.45).

Yield 0.10 g, 0.42 mmol, 45%.

^{1}H NMR (400 MHz, 298 K, DMSO): δ_H 9.89 (s, 1H, CHO), 8.58 (s, 1H, NCH), 7.95 (d, $^3J_{HH}$ = 8.0 Hz, 1H, NCCH), 7.66 (d, $^3J_{HH}$ = 8.5 Hz, 1H, NCCHC*H*), 7.52 (s, 4H, Ph), 5.35 (s, 2H, CH_2), 4.24 (s, 1H, C≡CH).

^{13}C{^{1}H} NMR (101 MHz, 298 K, DMSO): δ_C 192.0 (CHO), 159.1 (CO), 144.4 (*C*CHO), 139.7 (NCH), 136.3 (CH_2*C*), 132.6 (CH_2CCHC*H*), 127.9 (CH_2C*C*H), 123.0 (NCCHC*H*), 121.9 (NC*C*H), 86.2 (*C*≡CH), 83.5 (C≡*C*H), 68.6 (CH_2).

MS (ESI): m/z 260.1 $[M+Na]^+$.

FTIR: υ cm^{-1} 3210 m, 2806 w, 1704 s, 1573 s, 1451 m, 1262 s, 1220 s, 1031 m, 1015 m, 851 m, 815 s, 733 m, 679 m, 609 s, 530 m, 413 m.

Elemental analysis found (calculated for $C_{11}H_{15}NO_2$) % C 76.03 (75.94), H 4.99 (4.67), N 5.86 (5.90).

1.3 Synthesis and characterisation of chiral diamines

(*R*,*R*)-α,α'-*bis*(2-amino-2-phenylethoxy)-*m*-xylene [(*R*,*R*)-**5**]

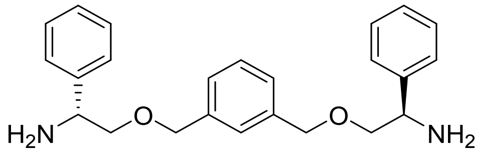

(*R*)-2-Phenylglycinol (*R*-**1**) (1.14 g, 8.25 mmol, 2.2 eq.) was dissolved in anhydrous THF (50 ml) under inert atmosphere. [15]-Crown-[5] (1.2 ml, 1.3 g, 6.0 mmol, 1.6 eq.) was added by injection. The solution was then added dropwise to a stirred solution of sodium hydride (0.42 g, 17 mmol, 4.6 eq.) in anhydrous THF (50 ml). The mixture was placed under partial vacuum and stirred at ambient temperature for 1 h. A solution of α,α'-dibromo-*m*-xylene (1.0 g, 3.8 mmol, 1 eq.) in anhydrous THF (50 ml) was added dropwise. The mixture was placed under partial vacuum and stirred at ambient temperature for 1 h, then for 24 h at 65 °C. The deep pink reaction mixture was allowed to cool before quenching with 2:1 saturated potassium chloride aq./water (60 ml). The crude was extracted using diethyl ether (3 × 75 ml), dried over sodium sulfate, filtered through celite, and the solvent removed under reduced pressure to leave a yellow oil. The product was obtained by silica gel column chromatography (DCM/MeOH/triethylamine, 500:10:8 v/v/v, R_f = 0.54) as a yellow oil.

Yield: 0.97 g, 2.6 mmol, 68 %.

^{1}H NMR (400 MHz, 298 K, $CDCl_3$): δ_H 7.40-7.21 (m, 14H, Ph), 4.54 (s, 4H, OCH_2Ph), 4.25 (dd, $^3J_{HH}$ = 9.5 Hz, 4.0 Hz, 2H, OCH_2C*H*), 3.61 (dd, $^2J_{HH}$ = 9.0 Hz, $^3J_{HH}$ = 4.0 Hz, 2H, OCH_2CH), 3.46 (t, $^2J_{HH}/^3J_{HH}$ = 9.0 Hz, 2H, OCH_2CH), 1.73 (br s, 4H, NH_2).

^{13}C{^{1}H} NMR (101 MHz, 298 K, $CDCl_3$): δ_C 142.4/138.4/128.4/128.3/127.5/126.9/ 126.4 (Ph), 76.6 (O*C*H_2CH), 73.2 (O*C*H_2Ph), 55.7 (OCH_2*C*H).

MS (ESI): m/z 399.3 $[M+Na]^+$, 377.3 $[M+H]^+$.

FTIR: υ cm^{-1} 3058 w, 3027 w, 2852 w, 1492 m, 1452 m, 1353 m, 1154 m, 1105 s, 1081 s, 759 s, 701 s.

(*S*,*S*)-α,α'-*bis*(2-amino-2-phenylethoxy)-*m*-xylene [(*S*,*S*)-**5**]

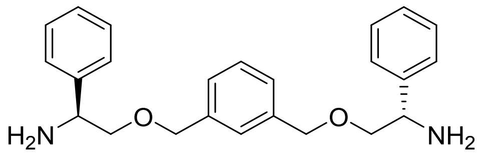

Compound (*S*,*S*)-**5** was synthesised using the procedure described for (*R*,*R*)-**5**, substituting (*R*)-2-phenylglycinol (*R*-**1**) for (*S*)-2-phenylglycinol (*S*-**1**).

Yield: 1.04 g, 2.8 mmol, 73 %.

^{1}H NMR (400 MHz, 298 K, $CDCl_3$): δ_H 7.42-7.21 (m, 14H, Ph), 4.52 (s, 4H, OCH_2Ph), 4.21 (dd, $^{3}J_{HH}$ = 9.0 Hz, 4.0 Hz, 2H, OCH_2C*H*), 3.61 (dd, $^{2}J_{HH}$ = 9.0 Hz, $^{3}J_{HH}$ = 3.5 Hz, 2H, OCH_2CH), 3.44 (t, $^{2}J_{HH}$/$^{3}J_{HH}$ = 9.0 Hz, 2H, OCH_2CH), 1.77 (br s, 4H, NH_2).

^{13}C{^{1}H} NMR (101 MHz, 298 K, $CDCl_3$): δ_C 142.2/138.2/128.4/128.2/127.7/126.8/ 126.6/ 126.2 (Ph), 76.6 (O*C*H_2CH), 73.4 (O*C*H_2Ph), 55.5 (OCH_2*C*H).

MS (ESI): m/z 399.3 [M+Na]$^+$, 377.3 [M+H]$^+$.

FTIR: υ cm^{-1} 3058 w, 3027 w, 2855 w, 1492 m, 1452 m, 1353 m, 1154 m, 1084 s, 759 s, 701 s.

(*R*,*R*)-α,α'-*bis*(2-amino-2-phenylethoxy)-*p*-xylene [(*R*,*R*)-**6**]

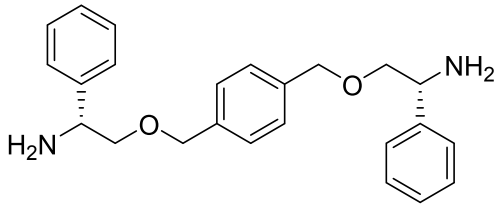

Compound (*R*,*R*)-**6** was synthesised using the procedure described for (*R*,*R*)-**5**, substituting α,α'-dibromo-*m*-xylene for α,α'-dibromo-*p*-xylene.

Yield: 1.12 g, 3.0 mmol, 79 %.

^{1}H NMR (400 MHz, 298 K, $CDCl_3$): δ_H 7.44-7.23 (m, 14H, Ph), 4.55 (s, 4H, OCH_2Ph), 4.21 (dd, $^{3}J_{HH}$ = 9.0 Hz, 4.0 Hz, 2H, OCH_2C*H*), 3.61 (dd, $^{2}J_{HH}$ = 9.0 Hz, $^{3}J_{HH}$ = 4.0 Hz, 2H, OCH_2CH), 3.44 (t, $^{2}J_{HH}$/$^{3}J_{HH}$ = 9.0 Hz, 2H, OCH_2CH), 1.79 (br s, 4H, NH_2).

^{13}C{^{1}H} NMR (101 MHz, 298 K, $CDCl_3$): δ_C 142.6/137.8/128.6/128.0/127.5/127.0 (Ph), 76.8 (O*C*H_2CH), 73.2 (O*C*H_2Ph), 55.8 (OCH_2*C*H).

MS (ESI): m/z 399.3 [M+Na]$^+$, 377.3 [M+H]$^+$.

FTIR: υ cm^{-1} 3056 w, 3026 w, 2854 w, 1492 m, 1452 m, 1356 m, 1088 s, 1020 m, 759 s, 701 s.

(*S*,*S*)-α,α'-*bis*(2-amino-2-phenylethoxy)-*p*-xylene [(*S*,*S*)-**6**]

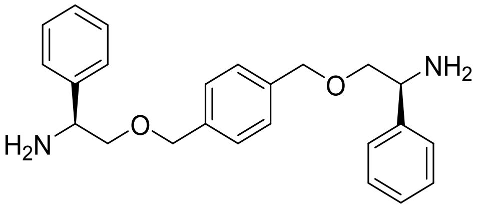

Compound (*S*,*S*)-**6** was synthesised using the procedure described for (*R*,*R*)-**5**, substituting (*R*)-2-phenylglycinol (*R*-**1**) for (*S*)-2-phenylglycinol (*S*-**1**) and α,α'-dibromo-*m*-xylene for α,α'-dibromo-*p*-xylene.

Yield: 1.02 g, 2.7 mmol, 72 %.

^{1}H NMR (400 MHz, 298 K, $CDCl_3$): δ_H 7.44-7.25 (m, 14H, Ph), 4.54 (s, 4H, OC*H*$_2$Ph), 4.21 (dd, $^3J_{HH}$ = 9.0 Hz, 4.0 Hz, 2H, OCH_2C*H*), 3.60 (dd, $^2J_{HH}$ = 9.0 Hz, $^3J_{HH}$ = 3.5 Hz, 2H, OC*H*$_2$CH), 3.45 (t, $^2J_{HH}/^3J_{HH}$ = 9.0 Hz, 2H, OC*H*$_2$CH), 1.79 (br s, 4H, NH_2).

^{13}C{^{1}H} NMR (101 MHz, 298 K, $CDCl_3$): δ_C 142.5/137.7/128.4/127.8/127.4/126.9 (Ph), 76.6 (O*C*H$_2$CH), 73.0 (O*C*H$_2$Ph), 55.6 (OCH_2*C*H).

MS (ESI): m/z 399.3 $[M+Na]^+$, 377.3 $[M+H]^+$.

FTIR: υ cm^{-1} 3058 w, 3026 w, 2854 w, 1514 m, 1492 m, 1452 m, 1355 m, 1086 s, 1020 m, 759 s, 701 s.

1.4 Synthesis and characterisation of chiral amines

(*R*)-2-(2,2'-Bipyridin-5-ylmethoxy)-1-phenylethanamine [(*R*)-**7**][11]

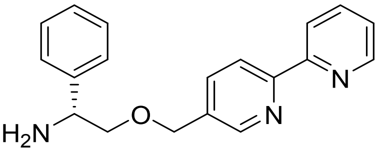

(*R*)-2-Phenylglycinol (1.23 g, 8.89 mmol, 1 eq.) was dissolved in anhydrous THF (20 ml) and added dropwise to a stirred suspension of sodium hydride (0.36 g, 15 mmol, 1.7 eq.) in anhydrous THF (10 ml) under argon atmosphere. The solution was stirred for 1 h at room temperature. A solution of 5-(chloromethyl)-2,2'-bipyridine (1.82 g, 8.89 mmol, 1 eq.) in anhydrous THF (20 ml) was added dropwise and the solution was stirred for 1 h at room temperature before heating at reflux (65 ˚C) for a further 2 h. The mixture was cooled to ambient temperature and brine (40 ml) was added into the solution. The product was extracted with diethyl ether (4 × 60 ml), dried over sodium sulphate and the solvent was removed under reduced pressure to leave an orange oil. The crude product was purified by silica gel flash chromatography (petroleum ether/EtOAc/triethylamine, 8:8:1 v/v/v, R_f = 0.45) to give the (*R*)-2-(2,2'-bipyridin-5-ylmethoxy)-1-phenylethanamine product as a yellow oil.

Yield: 1.77 g, 5.80 mmol, 65%.

^{1}H NMR (400 MHz, 298 K, $CDCl_3$) δ 8.68 (1H, d, $^3J_{HH}$ = 7.0 Hz, NCH), 8.63 (1H, d, $^4J_{HH}$ = 1.0 Hz, NCH), 8.38 (2H, dd, $^3J_{HH}$ = 8.0 Hz, $^4J_{HH}$ = 4.0 Hz, NCCH), 7.80 (2H, ddd, $^3J_{HH}$ = 8.0, 7.5 Hz, $^4J_{HH}$ = 4.0 Hz), 7.43-7.27 (6H, m, Ph, NCHC*H*), 4.63 (2H, s, OCH_2bpy), 4.26 (1H, dd, $^3J_{HH}$ = 8.5 Hz, $^4J_{HH}$ = 4.0 Hz, CH_2C*H*), 3.66 (1H, dd, $^3J_{HH}$ = 9.0 Hz, $^4J_{HH}$ = 4.0 Hz, CH_2), 3.51 (1H, t, $^3J_{HH}$ = 9.0 Hz, CH_2), 1.69 (2H, s, NH_2).

^{13}C {^{1}H} NMR (101 MHz, 298 K, $CDCl_3$) δ 156.0/155.7/149.2/148.6/142.3 (Ph-C, bpy-C) 137.0/136.4/133.7/128.5/127.5/126.8/123.7/121.1/120.8 (Ph-CH, bpy-CH), 77.4 (CH_2-bpy), 70.7 (*C*H$_2$CH), 55.6 (CH_2*C*H).

MS (ESI): m/z 306.2 $[M+H]^+$.

FTIR: υ cm^{-1} 3300 w, 3048 w, 3029 w, 2899 w, 2843 w, 1572 w, 1562 w, 1564 w, 1497 w, 1447 m, 1423 w, 1413 w, 1388 w, 1251 m, 1090 m, 1026 m, 984 w, 928 w.

(*S*)-2-(2,2'-Bipyridin-5-ylmethoxy)-1-phenylethanamine [(*S*)-**7**][11]

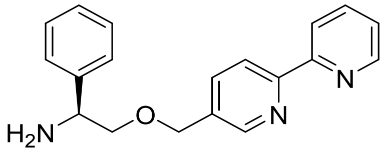

Compound [(*S*)-**7**] was synthesised using the procedure described for [(*R*)-**7**], substituting (*R*)-2-phenylglycinol for (*S*)-2-phenylglycinol.

Yield: 1.92 g, 6.28 mmol, 71%.

^{1}H NMR (400 MHz, 298 K, $CDCl_3$) δ 8.69 (1H, d, $^3J_{HH}$ = 7.0 Hz, NCH), 8.63 (1H, d, $^4J_{HH}$ = 1.0 Hz, NCH), 8.37 (2H, dd, $^3J_{HH}$ = 7.5 Hz, $^4J_{HH}$ = 4.0 Hz, NCCH), 7.80 (2H, ddd, $^3J_{HH}$ = 8.0 Hz, 7.5 Hz, $^4J_{HH}$ = 4.0 Hz), 7.41-7.24 (6H, m, Ph, NCHC*H*), 4.63 (2H, s, OCH_2bpy), 4.26 (1H, dd, $^3J_{HH}$ = 8.5 Hz, $^4J_{HH}$ = 4.0 Hz, CH_2CH), 3.64 (1H, dd, $^3J_{HH}$ = 9.0 Hz, $^4J_{HH}$ = 3.5 Hz, CH_2), 3.51 (1H, t, $^3J_{HH}$ = 9.0 Hz, CH_2), 1.70 (2H, s, NH_2).

^{13}C {^{1}H} NMR (101 MHz, 298 K, $CDCl_3$) δ 156.1/155.7/149.2/148.4/142.7 (Ph-C, bpy-C) 137.2/136.1/133.7/128.5/127.3/126.8/123.8/121.0/120.8 (Ph-CH, bpy-CH), 77.7 (CH_2-bpy), 70.3 (CH_2CH), 55.2 (CH_2CH).

MS (ESI): m/z 306.2 $[M+H]^+$.

FTIR: υ cm^{-1} 3302 w, 3047 w, 3028 w, 2899 w, 2841 w, 1570 w, 1565 w, 1561 w, 1497 w, 1449 m, 1429 w, 1410 w, 1385 w, 1251 m, 1092 m, 1028 m, 984 w, 931 w.

1.5 Synthesis and characterisation of $[^{56/57}Fe_2L_3]Cl_4$ flexicates

The diamine (3.0 eq.) and corresponding pyridinecarboxaldehyde (6.0 eq.) were dissolved in methanol (25 ml) and stirred for 24 h at ambient temperature to form a yellow solution. Anhydrous $^{56/57}$Fe(II) chloride (2.0 eq.) was added and an instantaneous colour change to deep purple was observed. The solution was then heated at reflux (80 °C) for 48 h then concentrated under reduced pressure. The crude was dissolved in minimum methanol (~2 ml) then pipetted into ethyl acetate (75 ml). The precipitate was filtered by fine filter paper, washed with ethyl acetate (3×25 ml), dissolved in methanol and the solvent was removed under reduced pressure to give the desired product as a dark purple solid, which was dried overnight at 50 °C *in vacuo.* Complexes were fully characterised, with ^{57}Fe compound spectra similar to the ^{56}Fe analogues.

Δ_{Fe}-[$Fe_2L^{5c}_3$]Cl_4·9H_2O (Δ_{Fe}-**9c**)

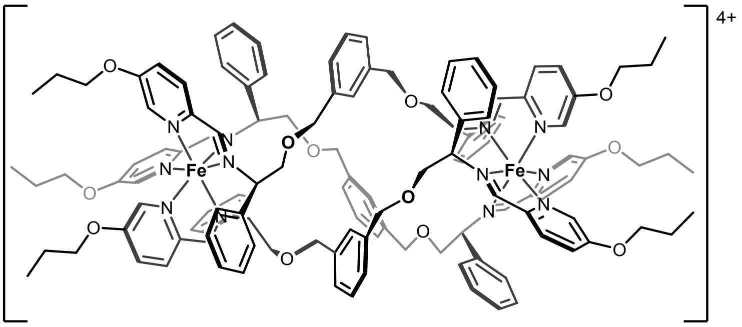

Δ_{Fe}-[Fe_2**L**$^{5c}_3$]$^{4+}$

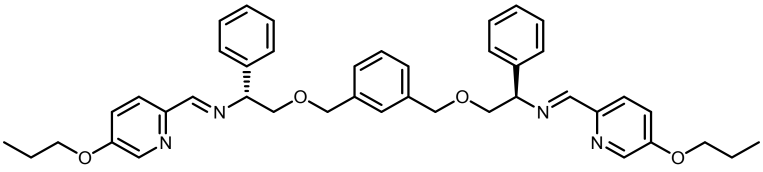

(*R*,*R*)-**L**^{5c}

0.05 g (0.13 mmol) of diamine used.

Yield: 0.08 g, 0.03 mmol, 77%.

^{1}H NMR (500 MHz, 298 K, MeOD): δ_H 9.11 (s, 6H, N=CH), 8.33 (s, 3H, OCH_2CC*H*C), 7.65 (d, $^3J_{HH}$ = 7.5 Hz, 6H, OCH_2CC*H*CHC*H*C), 7.52-7.43 (m, 12H, NCCH, OCH_2CCHC*H*CHC), 7.27 (dd, $^3J_{HH}$ = 9.0 Hz, $^4J_{HH}$ = 2.5 Hz, 6H, NCCHC*H*), 7.06 (t, $^3J_{HH}$ = 7.0 Hz, 6H, OCH_2CHCCHCHC*H*), 6.91 (t, $^3J_{HH}$ = 7.5 Hz, 12H, OCH_2CHCC*H*), 6.60 (s, 12H, OCH_2CHCCHC*H*), 6.36 (d, $^3J_{HH}$ = 2.5 Hz, 6H, NC*H*CO), 5.75 (dd, $^3J_{HH}$ = 11.5, 2.5 Hz, 6H, OCH_2C*H*), 5.00 (d, $^2J_{HH}$ = 11.0 Hz, 6H, OC*H*$_2$Ph), 4.37 (t, $^2J_{HH}$/$^3J_{HH}$ = 11.5 Hz, 6H, OC*H*$_2$CH), 3.91 (t, $^3J_{HH}$ = 6.0 Hz, 12H, OC*H*$_2$CH$_2$), 3.02 (d, $^2J_{HH}$ = 11.5 Hz, $^3J_{HH}$ = 3.0 Hz, 6H, OC*H*$_2$CH), 1.74-1.65 (m, 12H, C*H*$_2$CH$_3$), 0.94 (t, $^3J_{HH}$ = 7.5 Hz, 18H, CH_3). Second OC*H*$_2$Ph peak obscured by H_2O peak at 4.87 ppm (observed in 2D-NMR).

^{13}C{^{1}H} NMR (126 MHz, 298 K, MeOD): δ_C 169.8 (C=N), 157.7 (*C*C=N), 151.5 (NCH*C*O), 142.7 (*C*HCC=N), 138.0 (OCH_2*C*), 135.2 (OCH_2CH*C*), 129.9 (OCH_2CHC*C*H), 129.0 (OCH_2CCH*C*H), 128.9 (N*C*HCO), 128.4 (OCH_2CHCCH*C*H), 128.0 (OCH_2CHC*C*H), 127.4 (NCHC*C*H), 127.0 (OCH_2CHCCHCH*C*H), 121.1 (OCH_2C*C*HC), 74.5 (O*C*H$_2$C), 73.0 (O*C*H$_2$CH), 70.7 (O*C*H$_2$CH$_2$), 70.0 (OCH_2*C*H), 21.6 (*C*H$_2$CH$_3$), 9.00 (CH_3).

HRMS: Calculated for [Fe_2L_3]$^{4+}$ m/z 530.7312, found m/z 530.7270.

FTIR: υ cm^{-1} 3353 w (br), 3029 w, 2970 w, 2938 w, 2874 w, 1593 m, 1558 s, 1496 m, 1453 m, 1304 m, 1278 s, 1233 s, 1074 m, 1003 m, 967 m, 759 m, 699 s.

Elemental Analysis found (calculated for $C_{126}H_{138}Cl_4Fe_2N_{12}O_{12}$·8$H_2O$): % C 62.45 (62.79), H 6.54 (6.44), N 6.59 (6.97).

Λ_{Fe}-[$Fe_2L^{5c}{}_3$]Cl_4·9H_2O (Λ_{Fe}-9c)

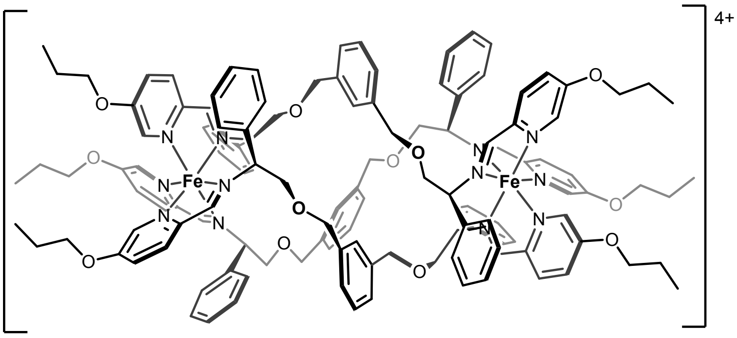

Λ_{Fe}-[Fe_2**L**$^{5c}{}_3$]$^{4+}$

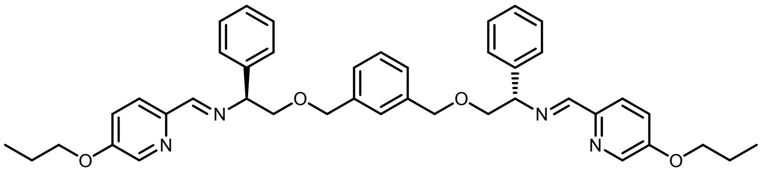

(*S*,*S*)-**L**^{5c}

0.05 g (0.13 mmol) of diamine used.

Yield: 0.08 g, 0.03 mmol, 76%.

^{1}H NMR (500 MHz, 298 K, MeOD): δ_H 9.10 (s, 6H, N=CH), 8.33 (s, 3H, OCH_2CC*H*C), 7.65 (d, $^3J_{HH}$ = 7.5 Hz, 6H, OCH_2CC*H*CHC*H*C), 7.54-7.42 (m, 12H, NCCH, OCH_2CCHC*H*CHC), 7.27 (dd, $^3J_{HH}$ = 9.0 Hz, $^4J_{HH}$ = 2.5 Hz, 6H, NCCHC*H*), 7.06 (t, $^3J_{HH}$ = 7.5 Hz, 6H, OCH_2CHCCHCHC*H*), 6.91 (t, $^3J_{HH}$ = 7.5 Hz, 12H, OCH_2CHCC*H*), 6.61 (s, 12H, OCH_2CHCCHC*H*), 6.36 (d, $^3J_{HH}$ = 2.5 Hz, 6H, NC*H*CO), 5.75 (dd, $^3J_{HH}$ = 11.5, 3.0 Hz, 6H, OCH_2C*H*), 5.00 (d, $^2J_{HH}$ = 11.0 Hz, 6H, OCH_2Ph), 4.37 (t, $^2J_{HH}$/$^3J_{HH}$ = 11.5 Hz, 6H, OCH_2CH), 3.91 (td, $^3J_{HH}$ = 6.5 Hz, $^4J_{HH}$ = 1.5 Hz, 12H, OCH_2CH_2), 3.02 (d, $^2J_{HH}$ = 11.5 Hz, $^3J_{HH}$ = 3.0 Hz, 6H, OCH_2CH), 1.75-1.64 (m, 12H, CH_2CH_3), 0.94 (t, $^3J_{HH}$ = 7.5 Hz, 18H, CH_3). Second OCH_2Ph peak obscured by H_2O peak at 4.87 ppm (observed in 2D-NMR).

^{13}C{^{1}H} NMR (126 MHz, 298 K, MeOD): δ_C 169.8 (C=N), 157.7 (*C*C=N), 151.5 (NCH*C*O), 142.7 (*C*HCC=N), 138.0 (OCH_2*C*), 135.2 (OCH_2CH*C*), 129.9 (OCH_2CHCCH), 129.2 (OCH_2CCH*C*H), 128.9 (N*C*HCO), 128.4 (OCH_2CHCCH*C*H), 128.0 (OCH_2CHC*C*H), 127.4 (NCHC*C*H), 127.0 (OCH_2CHCCHCH*C*H), 121.1 (OCH_2C*C*HC), 74.5 (O*C*H_2C), 73.0 (O*C*H_2CH), 70.7 (O*C*H_2CH_2), 70.0 (OCH_2*C*H), 21.6 (*C*H_2CH_3), 9.00 (CH_3).

HRMS: Calculated for [Fe_2L_3]$^{4+}$ m/z 530.7312, found m/z 530.7279.

FTIR: υ cm^{-1} 3370 w (br), 3032 w, 2963 w, 2935 w, 2873 w, 1592 m, 1556 s, 1492 m, 1452 m, 1303 m, 1277 s, 1232 s, 1073 m, 1002 m, 966 m, 758 m, 700 s.

Elemental Analysis found (calculated for $C_{126}H_{138}Cl_4Fe_2N_{12}O_{12}$·8$H_2O$): % C 62.38 (62.79), H 6.66 (6.44), N 6.60 (6.97).

Δ_{Fe}-[$Fe_2L^{5d}_3$]Cl_4·$9H_2O$ (Δ_{Fe}-9d)

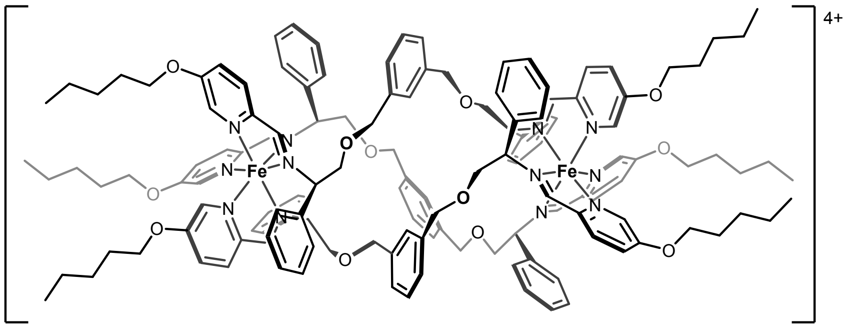

Δ_{Fe}-[Fe_2**L**$^{5d}_3$]$^{4+}$

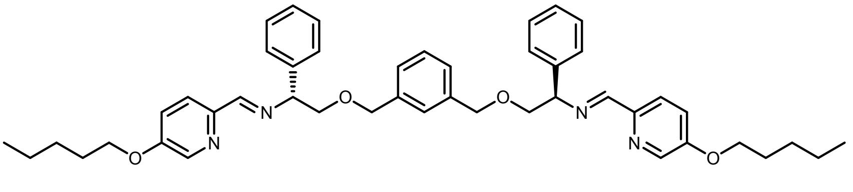

(*R*,*R*)-**L**^{5d}

0.21 g (0.56 mmol) of diamine used.

Yield: 0.42 g, 0.16 mmol, 87%.

^{1}H NMR (500 MHz, 298 K, MeOD): δ_H 9.10 (s, 6H, N=CH), 8.32 (s, 3H, OCH$_2$CC*H*C), 7.63 (d, $^3J_{HH}$ = 7.5 Hz, 6H, OCH$_2$CC*H*CHC*H*C), 7.50-7.44 (m, NCCH, OCH$_2$CCHC*H*CHC), 7.35 (t, $^3J_{HH}$ = 7.5 Hz, 6H, NCCHC*H*), 7.04 (t, $^3J_{HH}$ = 7.0 Hz, 6H, OCH$_2$CHCCHCHC*H*), 6.87 (s, 12H, OCH$_2$CHCCC*H*), 6.60 (s, 12H, OCH$_2$CHCCHC*H*), 6.36 (d, $^3J_{HH}$ = 2.5 Hz, 6H, NC*H*CO), 5.74 (dd, $^3J_{HH}$ = 9.0, 2.5 Hz, 6H, OCH$_2$C*H*), 4.99 (d, $^2J_{HH}$ = 10.5 Hz, 6H, OC*H*$_2$Ph), 4.84 (d, $^2J_{HH}$ = 11.0 Hz, 6H, OC*H*$_2$Ph), 4.36 (t, $^2J_{HH}$/$^3J_{HH}$ = 11.5 Hz, 6H, OC*H*$_2$CH), 3.93 (t, $^3J_{HH}$ = 6.5 Hz, 12H, OC*H*$_2$CH$_2$), 3.00 (d, $^2J_{HH}$ = 9.0 Hz, $^3J_{HH}$ = 3.0 Hz, 6H, OC*H*$_2$CH), 1.72-1.63 (m, 12H, OCH$_2$C*H*$_2$), 1.38-1.28 (m, 24H, C*H*$_2$C*H*$_2$CH$_3$), 0.89 (t, $^3J_{HH}$ = 6.5 Hz, 18H, CH$_3$). Second OC*H*$_2$Ph peak obscured by H$_2$O peak at 4.87 ppm (observed in 2D-NMR).

^{13}C{^{1}H} NMR (126 MHz, 298 K, MeOD): δ_C 169.9 (C=N), 157.6 (*C*C=N), 151.5 (NCH*C*O), 142.6 (*C*HCC=N), 138.0 (OCH$_2$*C*), 135.2 (OCH$_2$CH*C*), 134.2 (OCH$_2$C*C*HCH), 129.9 (OCH$_2$CHC*C*H), 129.0 (N*C*HCO), 128.4 (OCH$_2$CHCCHC*H*), 128.0 (OCH$_2$CHC*C*H), 127.3 (NCHC*C*H), 127.1 (OCH$_2$CHCCHCHC*H*), 124.4 (OCH$_2$C*C*HC), 74.5 (O*C*H$_2$C), 73.0 (O*C*H$_2$CH), 70.0 (OCH$_2$*C*H), 69.2 (O*C*H$_2$CH$_2$), 28.0 (OCH$_2$*C*H$_2$), 27.5 (*C*H$_2$CH$_2$CH$_3$), 22.0 (*C*H$_2$CH$_3$), 12.8 (CH$_3$).

HRMS: Calculated for [Fe_2L_3]$^{4+}$ m/z 572.7782, found m/z 572.7744.

FTIR: υ cm^{-1} 3327 w (br), 3028 w, 2928 w, 2860 w, 1591 m, 1556 s, 1492 m, 1452 m, 1301 m, 1232 s, 1073 m, 1001 m, 757 m, 698 s.

Elemental Analysis found (calculated for $C_{138}H_{162}Cl_4Fe_2N_{12}O_{12}$·$9H_2O$): % C 63.49 (63.84), H 7.04 (6.99), N 6.11 (6.47).

Λ_{Fe}-[$Fe_2L^{5d}{}_3$]Cl_4·9H_2O (Λ_{Fe}-9d)

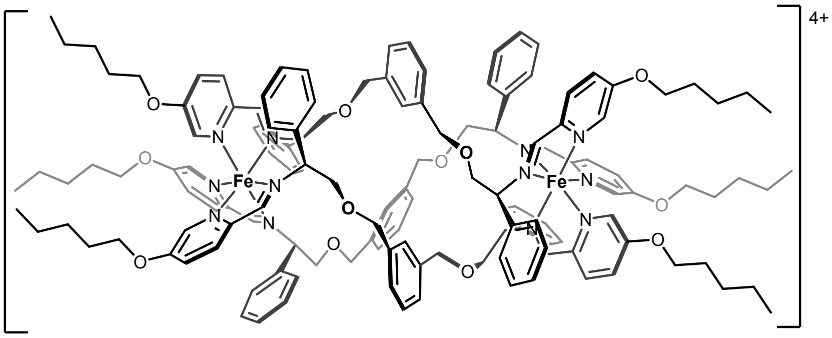

Λ_{Fe}-[Fe_2**L**$^{5d}{}_3$]$^{4+}$

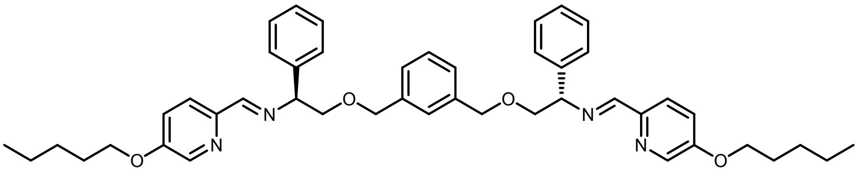

(S,S)-**L^{5d}**

0.21 g (0.56 mmol) of diamine used.

Yield: 0.40 g, 0.15 mmol, 83%.

^{1}H NMR (500 MHz, 298 K, MeOD): δ_H 9.12 (s, 6H, N=CH), 8.30 (s, 3H, OCH_2CC*H*C), 7.66 (d, $^3J_{HH}$ = 7.5 Hz, 6H, NCC*H*), 7.52-7.42 (m, 21H, NC*H*CO, OCH_2CHCCC*H*, OCH_2CC*H*CH), 7.35 (t, $^3J_{HH}$ = 7.5 Hz, 6H, NCCHC*H*), 7.03 (t, $^3J_{HH}$ = 7.5 Hz, 6H, OCH_2CHCC*H*), 6.88 (s, 12H, $OCH_2CHCCHC$*H*), 6.58 (s, 6H, $OCH_2CHCCHCHC$*H*), 5.73 (dd, $^3J_{HH}$ = 9.5, 2.5 Hz, 6H, OCH_2C*H*), 4.99 (d, $^2J_{HH}$ = 10.5 Hz, 6H, OC*H*$_2$Ph), 4.85 (d, $^2J_{HH}$ = 11.0 Hz, 6H, OC*H*$_2$Ph), 4.37 (t, $^2J_{HH}$/$^3J_{HH}$ = 11.5 Hz, 6H, OC*H*$_2$CH), 3.91 (t, $^3J_{HH}$ = 6.0 Hz, 12H, OC*H*$_2$CH$_2$), 3.00 (d, $^2J_{HH}$ = 8.5 Hz, $^3J_{HH}$ = 3.0 Hz, 6H, OC*H*$_2$CH), 1.74–1.62 (m, 12H, OCH_2C*H*$_2$), 1.38–1.26 (m, 24H, C*H*$_2$C*H*$_2$CH$_3$), 0.90 (t, $^3J_{HH}$ = 6.0 Hz, 18H, CH_3).

^{13}C{^{1}H} NMR (126 MHz, 298 K, MeOD): δ_C 170.2 (C=N), 157.7 (*C*C=N), 151.5 (NCH*C*O), 142.6 (*C*HCC=N), 138.1 (OCH_2*C*), 135.3 (OCH_2CH*C*), 134.3 (OCH_2C*C*HCH), 129.7 (OCH_2CHCC*C*H), 129.0 (N*C*HCO), 128.8 (OCH_2CCH*C*H), 128.4 ($OCH_2CHCCCH$*C*H), 128.0 (OCH_2CHC*C*H), 127.2 (NCHC*C*H), 127.0 ($OCH_2CHCCHCH$*C*H), 124.3 (OCH_2C*C*HC), 74.6 (O*C*H$_2$C), 73.3 (O*C*H$_2$CH), 69.9 (OCH_2*C*H), 69.4 (O*C*H$_2$CH$_2$), 28.1 (OCH_2*C*H$_2$), 27.5 (*C*H$_2$CH$_2$CH$_3$), 22.0 (*C*H$_2$CH$_3$), 12.9 (CH_3).

HRMS: Calculated for [Fe_2L_3]$^{4+}$ m/z 572.7782, found m/z 572.7740.

FTIR: υ cm^{-1} 3323 w (br), 3029 w, 2928 w, 2862 w, 1589 m, 1557 s, 1491 m, 1450 m, 1301 m, 1232 s, 1074 m, 999 m, 757 m, 699 s.

Elemental Analysis found (calculated for $C_{138}H_{162}Cl_4Fe_2N_{12}O_{12}$·8$H_2O$): % C 64.04 (64.28), H 7.22 (6.96), N 6.53 (6.52).

Δ_{Fe}-[$Fe_2L^{5e}{}_3$]$Cl_4\cdot 12H_2O$ (Δ_{Fe}-9e)

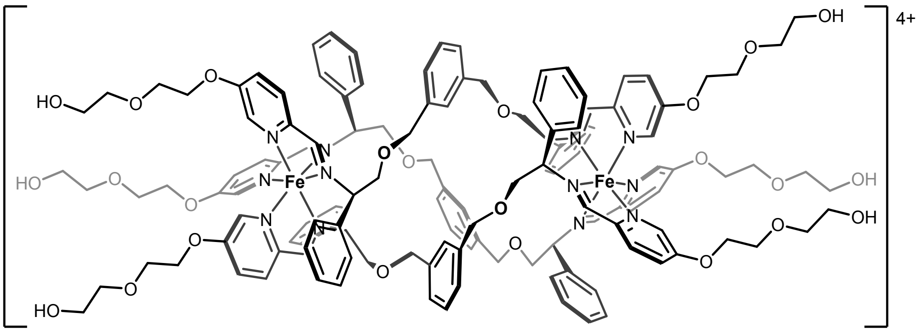

Δ_{Fe}-[Fe_2**L**$^{5e}{}_3$]$^{4+}$

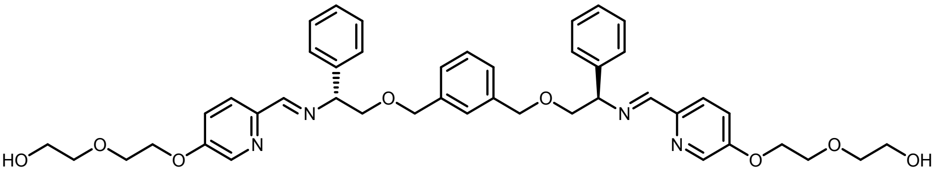

(R,R)-**L**^{5e}

0.20 g (0.53 mmol) of diamine used.

Yield: 0.43 g, 0.16 mmol, 89%.

^{1}H NMR (500 MHz, 298 K, MeOD): δ_H 8.96 (s, 6H, N=CH), 8.23 (s, 3H, OCH_2CC*H*C), 7.53 (d, $^3J_{HH}$ = 9.0 Hz, 6H, NCC*H*), 7.39 (t, $^3J_{HH}$ = 7.5 Hz, 3H, OCH_2CC*H*C*H*), 7.35-7.30 (m, 6H, NC*H*CO), 7.25-7.16 (m, 6H, NCCHC*H*), 6.94 (t, $^3J_{HH}$ = 7.5 Hz, 6H, OCH_2CHCC*H*), 6.81 (t, $^3J_{HH}$ = 6.0 Hz, 12H, OCH_2CHCC*H*), 6.52-6.41 (m, 12H, $OCH_2CHCCHC$*H*), 6.31 (s, 6H, $OCH_2CHCCHCHC$*H*), 5.62 (dd, $^3J_{HH}$ = 11.0, 2.5 Hz, 6H, OCH_2C*H*), 4.90 (d, $^2J_{HH}$ = 10.5 Hz, 6H, OC*H*$_2$Ph), 4.24 (t, $^2J_{HH}$/$^3J_{HH}$ = 12.0 Hz, 6H, OC*H*$_2$CH), 4.02 (s, 12H, PyOC*H*$_2$), 3.63 (s, 12H, $PyOCH_2C$*H*$_2$), 3.55-3.47 (m, 12H, C*H*$_2$$CH_2OH$), 3.41 (t, $^3J_{HH}$ = 4.5 Hz, 12H, CH_2C*H*$_2$OH), 2.92 (dd, $^3J_{HH}$ = 11.0, 3.0 Hz, 6H, OCH_2C*H*). Presence of water (δ_H 4.88-4.80) obscures one of the OC*H*$_2$Ph peaks, however it can be detected by 2D-NMR (HSQC and HMBC).

^{13}C{^{1}H} NMR (126 MHz, 298 K, MeOD): δ_C 170.0 (C=N), 157.3 (*C*C=N), 151.6 (NCH*C*O), 143.1 (*C*HCC=N), 138.3 (OCH_2*C*), 135.0 (OCH_2CH*C*), 129.9 (OCH_2C*C*HCH), 129.2 (N*C*HCO), 128.8 (OCH_2CCH*C*H), 128.5 (OCH_2CHCCH*C*H), 128.0 (OCH_2CHC*C*H), 127.2 (NCHC*C*H), 126.9 ($OCH_2CHCCHCH$*C*H), 121.7 (OCH_2*C*CHC), 74.4 (O*C*H_2C), 73.0 (O*C*H_2CH), 72.6 (C*H*$_2$OH), 70.1 (OCH_2*C*H), 68.7 ($PyOCH_2C$*H*$_2$), 68.5 (PyO*C*H_2), 60.3 (C*H*$_2$$CH_2OH$).

HRMS: Calculated for [Fe_2L_3]$^{4+}$ m/z 599.7394, found m/z 599.7357.

FTIR: υ cm^{-1} 3339 m (br), 3032 w, 2865 m, 1592 m, 1557 s, 1484 m, 1452 m, 1304 m, 1232 s, 1117 s, 1071 s, 1043 s, 928 m, 759 m, 700 s.

Elemental Analysis found (calculated for $C_{132}H_{150}Cl_4Fe_2N_{12}O_{24}\cdot 12H_2O$): % C 57.31 (57.48), H 6.64 (6.36), N 5.75 (6.09).

Λ_{Fe}-[$Fe_2L^{5e}_3$]$Cl_4\cdot12H_2O$ (Λ_{Fe}-9e)

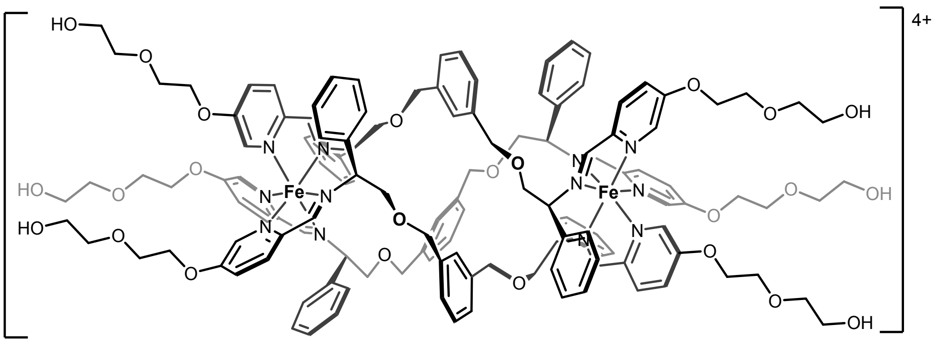

Λ_{Fe}-[Fe_2**L**$^{\mathbf{5e}}_3$]$^{4+}$

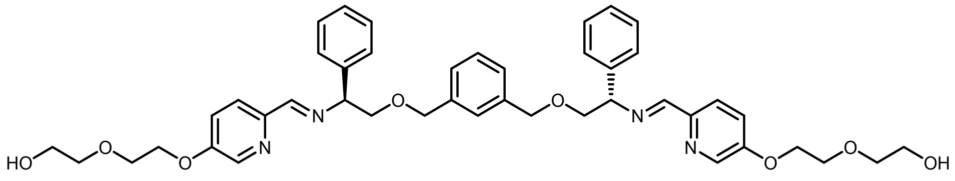

(*S*,*S*)-**L**$^{\mathbf{5e}}$

0.22 g (0.58 mmol) of diamine used.

Yield: 0.45 g, 0.17 mmol, 85%.

^{1}H NMR (500 MHz, 298 K, MeOD): δ_H 8.96 (s, 6H, N=CH), 8.23 (s, 3H, OCH_2CCHC), 7.53 (d, $^3J_{HH}$ = 7.5 Hz, 6H, NCC*H*), 7.40 (t, $^3J_{HH}$ = 7.0 Hz, 3H, OCH_2CCHCH), 7.37-7.29 (m, 6H, NC*H*CO), 7.24-7.16 (m, 6H, NCCHC*H*), 6.94 (t, $^3J_{HH}$ = 7.5 Hz, 6H, OCH_2CHCCH), 6.80 (t, $^3J_{HH}$ = 7.0 Hz, 12H, OCH_2CHCCH), 6.47 (s, 12H, $OCH_2CHCCHCH$), 6.31 (s, 6H, $OCH_2CHCCHCHCH$), 5.63 (dd, $^3J_{HH}$ = 10.5, 2.5 Hz, 6H, OCH_2CH), 4.89 (d, $^2J_{HH}$ = 10.5 Hz, 6H, OCH_2Ph), OCH_2Ph obscured by H_2O peak, 4.24 (t, $^2J_{HH}/^3J_{HH}$ = 11.0 Hz, 6H, OCH_2CH), 4.01 (s, 12H, $PyOCH_2$), 3.62 (s, 12H, $PyOCH_2CH_2$), 3.54-3.45 (m, 12H, CH_2CH_2OH), 3.42 (t, $^3J_{HH}$ = 5.0 Hz, 12H, CH_2OH), 2.91 (dd, $^3J_{HH}$ = 11.0, 3.5 Hz, 6H, OCH_2CH).

^{13}C{^{1}H} NMR (126 MHz, 298 K, MeOD): δ_C 169.9 (C=N), 157.5 (*C*C=N), 151.7 (NCH*C*O), 143.1 (*C*HCC=N), 138.0 (OCH_2C), 135.1 (OCH_2CHC), 129.8 (OCH_2CCHCH), 129.2 (N*C*HCO), 129.0 (OCH_2CCHCH), 128.4 ($OCH_2CHCCHCH$), 128.0 (OCH_2CHCCH), 127.3 (NCHC*C*H), 127.0 ($OCH_2CHCCHCHCH$), 121.9 (OCH_2CCHC), 74.5 (OCH_2C), 73.0 (OCH_2CH), 72.4 (CH_2OH), 70.0 (OCH_2CH), 68.6 ($PyOCH_2CH_2$), 68.5 ($PyOCH_2$), 60.6 (CH_2CH_2OH).

HRMS: Calculated for [Fe_2L_3]$^{4+}$ m/z 599.7394, found m/z 599.7345.

FTIR: υ cm^{-1} 3341 m (br), 3031 w, 2865 m, 1592 m, 1557 s, 1483 m, 1450 m, 1304 m, 1233 s, 1119 s, 1069 s, 1041 s, 926 m, 759 m, 699 s.

Elemental Analysis found (calculated for $C_{132}H_{150}Cl_4Fe_2N_{12}O_{24}\cdot12H_2O$): % C 57.27 (57.48), H 6.58 (6.36), N 5.81 (6.09).

Δ_{Fe}-[$Fe_2L^{5f}_3$]Cl_4·8H_2O (Δ_{Fe}-9f)

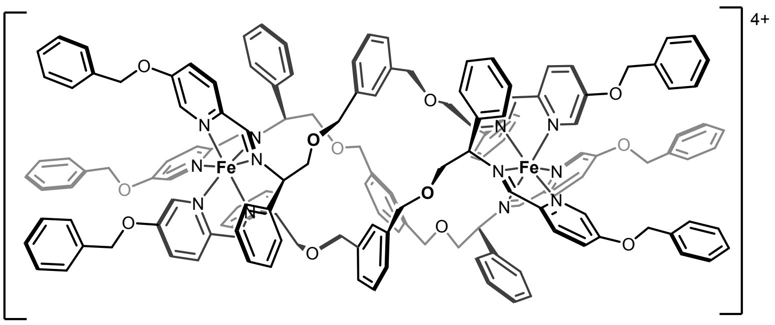

Δ_{Fe}-[Fe_2**L**$^{5f}_3$]$^{4+}$

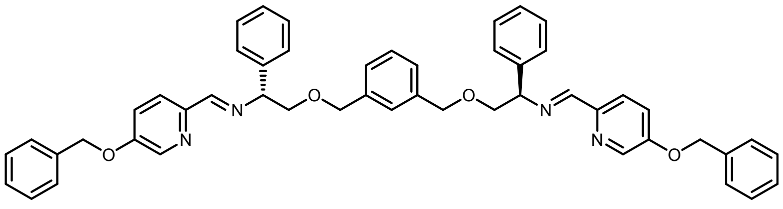

(*R*,*R*)-**L**^{5f}

0.21 g (0.56 mmol) of diamine used.

Yield: 0.42 g, 0.16 mmol, 84%.

^{1}H NMR (500 MHz, 298 K, MeOD): δ_H 8.97 (s, 6H, N=CH), 8.26 (s, 3H, OCH_2CC*H*C), 7.60 (d, $^3J_{HH}$ = 7.5 Hz, 6H, OCH_2CC*H*CHC*H*C), 7.46 (t, $^3J_{HH}$ = 7.5 Hz, 6H, OCH_2CCHC*H*CHC), 7.39-7.31 (m, 30H, OCH_2Ph-*H*), 7.25-7.17 (m, 12H, C*H*NCC*H*), 6.96 (t, $^3J_{HH}$ = 7.5 Hz, 6H, NCCHC*H*), 6.77 (s, 12H, OCH_2CHCC*H*), 6.43 (s, 12H, OCH_2CHCCHC*H*), 6.25 (s, 6H, OCH_2CHCCHCHC*H*), 5.64 (dd, $^3J_{HH}$ = 11.0, 2.0 Hz, 6H, OCH_2C*H*), 5.06 (q, $^4J_{HH}$ = 12.5 Hz, 12H, OC*H*$_2$Ph), 4.94 (d, $^2J_{HH}$ = 11.0 Hz, 6H, OC*H*$_2$PhCH_2), 4.79 (d, $^2J_{HH}$ = 11.0 Hz, 6H, OC*H*$_2$PhCH_2), 4.28 (t, $^2J_{HH}$/$^3J_{HH}$ = 11.5 Hz, 6H, OC*H*$_2$CH), 2.94 (d, $^3J_{HH}$ = 8.5 Hz, 6H, OC*H*$_2$CH).

^{13}C{^{1}H} NMR (126 MHz, 298 K, MeOD): δ_C 169.8 (C=N), 156.8 (*C*C=N), 151.8 (NCH*C*O), 141.7 (*C*HCC=N), 137.9 (COCH$_2$*C*), 137.9 (OCH$_2$*C*), 137.2 (O*C*CHN), 135.0 (OCH$_2$CH*C*), 129.8 (N*C*HCO), 129.0 (OCH$_2$CHC*C*H), 128.9 (OCH$_2$C*C*HCH*C*HC), 128.6 (OCH$_2$CHCCH*C*H), 128.5 (OCH$_2$C*C*HCHCHCH), 128.4 (OCH$_2$CCH*C*HCH*C*H), 127.9 (OCH$_2$CHCCHCH*C*H), 127.5 (NCCH*C*H), 127.3 (OCH$_2$CCH*C*HCHC), 127.0 (OCH$_2$CCHCH*C*HCH), 123.1 (OCH$_2$C*C*HC), 74.4 (O*C*H$_2$PhCH$_2$), 72.9 (O*C*H$_2$CH), 70.4 (O*C*H$_2$Ph), 69.9 (OCH$_2$*C*H).

HRMS: Calculated for [Fe_2L_3]$^{4+}$ m/z 602.7313, found m/z 602.7274.

FTIR: υ cm^{-1} 3329 w (br), 3029 w, 2863 w, 1590 m, 1556 m, 1493 m, 1452 m, 1301 m, 1226 s, 1073 m, 1001 m, 738 m, 697 s.

Elemental Analysis found (calculated for $C_{150}H_{138}Cl_4Fe_2N_{12}O_{12}$·8$H_2O$): % C 66.61 (66.77), H 5.82 (5.75), N 6.21 (6.23).

Λ_{Fe}-[Fe_2**L**$^{5f}{}_3$]$Cl_4 \cdot 8H_2O$ (**Λ_{Fe}-9f**)

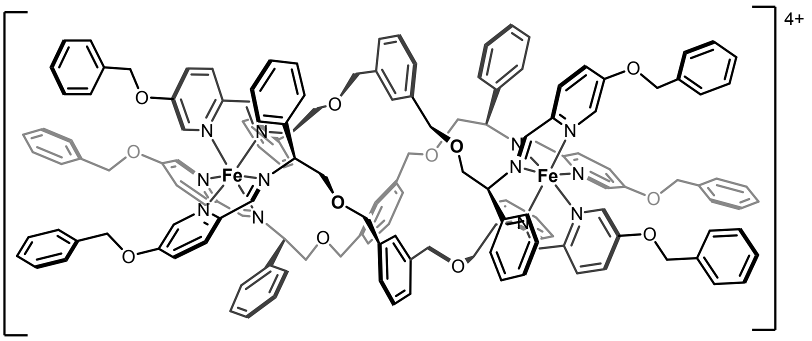

Λ_{Fe}-[Fe_2**L**$^{5f}{}_3$]$^{4+}$

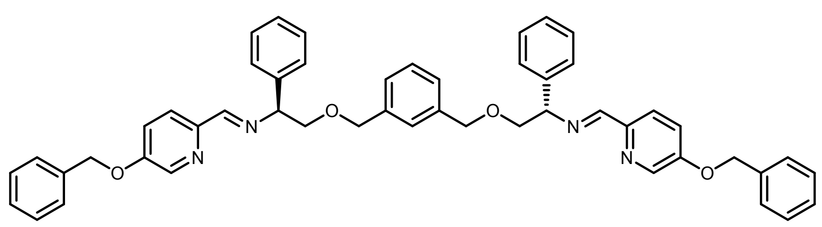

(*S*,*S*)-**L**^{5f}

0.21 g (0.56 mmol) of diamine used.

Yield: 0.44 g, 0.16 mmol, 88%.

^{1}H NMR (500 MHz, 298 K, MeOD): δ_H 8.95 (s, 6H, N=CH), 8.25 (s, 3H, OCH_2CCHC), 7.63 (d, $^3J_{HH}$ = 7.5 Hz, 6H, $OCH_2CCHCHCHC$), 7.45 (t, $^3J_{HH}$ = 7.5 Hz, 6H, $OCH_2CCHCHCHC$), 7.39-7.34 (m, 30H, OCH_2Ph-*H*), 7.22-7.15 (m, 12H, C*H*NCC*H*), 6.94 (t, $^3J_{HH}$ = 7.5 Hz, 6H, NCCHC*H*), 6.79 (s, 12H, OCH_2CHCCH), 6.43 (s, 12H, $OCH_2CHCCHCH$), 6.24 (s, 6H, $OCH_2CHCCHCHCH$), 5.64 (dd, $^3J_{HH}$ = 11.0, 2.0 Hz, 6H, OCH_2CH), 5.05 (q, $^4J_{HH}$ = 12.5 Hz, 12H, OCH_2Ph), 4.95 (d, $^2J_{HH}$ = 10.5 Hz, 6H, OCH_2PhCH_2), 4.77 (d, $^2J_{HH}$ = 11.0 Hz, 6H, OCH_2PhCH_2), 4.27 (t, $^2J_{HH}/^3J_{HH}$ = 11.5 Hz, 6H, OCH_2CH), 2.95 (d, $^3J_{HH}$ = 9.0 Hz, 6H, OCH_2CH).

$^{13}C\{^1H\}$ NMR (126 MHz, 298 K, MeOD): δ_C 169.9 (C=N), 156.9 (*C*C=N), 151.6 (NCH*C*O), 141.7 (*C*HCC=N), 137.9 ($COCH_2C$), 137.8 (OCH_2C), 137.4 (O*C*CHN), 135.1 (OCH_2CHC), 129.8 (N*C*HCO), 129.4 (OCH_2CHCCH), 128.9 ($OCH_2CCHCHCHC$), 128.7 ($OCH_2CHCCHCH$), 128.7 ($OCH_2CCHCHCHCH$), 128.2 ($OCH_2CCHCHCHCH$), 127.8 ($OCH_2CHCCHCHCH$), 127.6 (NCCH*C*H), 127.4 ($OCH_2CCHCHCHC$), 127.1 ($OCH_2CCHCHCHCH$), 123.1 (OCH_2CCHC), 74.4 (OCH_2PhCH_2), 72.9 (OCH_2CH), 70.6 (OCH_2Ph), 69.9 (OCH_2CH).

HRMS: Calculated for [Fe_2L_3]$^{4+}$ m/z 602.7313, found m/z 602.7276.

FTIR: υ cm^{-1} 3324 w (br), 3028 w, 2863 w, 1589 m, 1555 m, 1491 m, 1450 m, 1301 m, 1225 s, 1075 m, 1000 m, 738 m, 698 s.

Elemental Analysis found (calculated for $C_{150}H_{138}Cl_4Fe_2N_{12}O_{12} \cdot 8H_2O$): % C 66.58 (66.77), H 5.93 (5.75), N 6.06 (6.23).

Δ_{Fe}-[$Fe_2L^{5g}{}_3$]$Cl_4\cdot 6H_2O$ (Δ_{Fe}-9g)

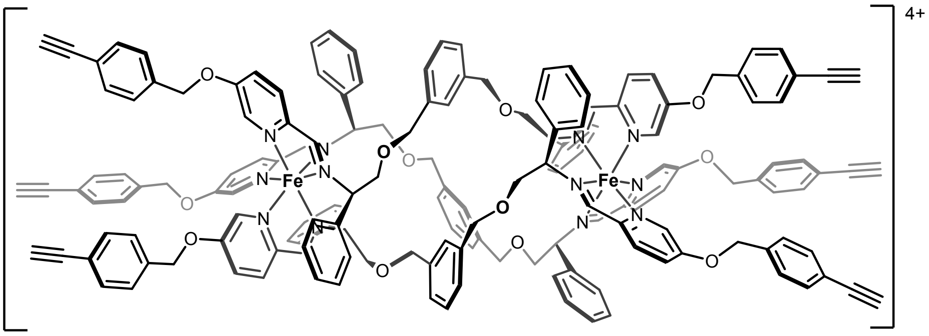

Δ_{Fe}-[Fe_2**L**$^{5g}{}_3$]$^{4+}$

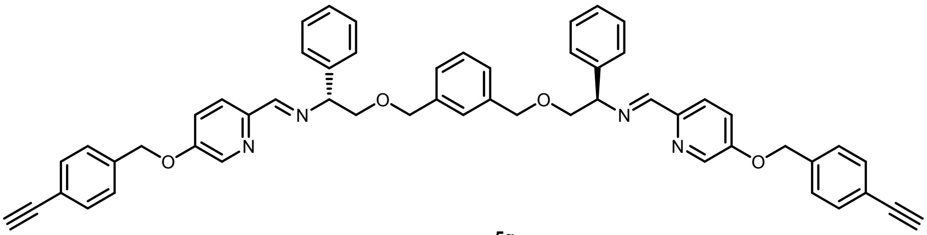

(*R*,*R*)-**L**^{5g}

0.21 g (0.56 mmol) of diamine used.

Yield: 0.45 g, 0.16 mmol, 86%.

^{1}H NMR (500 MHz, 298 K, MeOD): δ_H 9.00 (s, 6H, N=CH), 8.28 (s, 3H, OCH$_2$CC*H*C), 7.60 (d, $^{3}J_{HH}$ = 7.5 Hz, 6H, OCH$_2$CC*H*CHCH), 7.46 (m, 15H, OCH$_2$CCHC*H*CH, C*H*CC≡CH), 7.35 (d, $^{3}J_{HH}$ = 8.0 Hz, 12H, C*H*CHCC≡CH), 7.25-7.22 (m, 12H, C*H*NCC*H*), 6.98 (t, $^{3}J_{HH}$ = 7.5 Hz, 6H, NCCHC*H*), 6.80 (s, 12H, OCH$_2$CHCC*H*), 6.49 (s, 12H, OCH$_2$CHCCHC*H*), 6.23 (s, 6H, OCH$_2$CHCCHCHC*H*), 5.66 (dd, $^{3}J_{HH}$ = 10.0, 2.5 Hz, 6H, OCH$_2$C*H*), 5.08 (s, 12H, OC*H*$_2$Ph-C≡CH), 4.94 (d, $^{2}J_{HH}$ = 11.0 Hz, 6H, OC*H*$_2$Ph), 4.79 (d, $^{2}J_{HH}$ = 11.0 Hz, 6H, OC*H*$_2$Ph), 4.29 (t, $^{2}J_{HH}$/$^{3}J_{HH}$ = 10.5 Hz, 6H, OC*H*$_2$CH), 3.68 (s, 6H, C≡CH), 2.96 (dd, $^{3}J_{HH}$ = 10.0, 2.0 Hz, 6H, OC*H*$_2$CH).

^{13}C{^{1}H} NMR (126 MHz, 298 K, MeOD): δ_C 171.2 (C=N), 155.9 (*C*C=N), 152.1 (NCH*C*O), 141.6 (*C*HCC=N), 139.8 (COCH$_2$*C*), 137.6 (CH$_2$OCH$_2$*C*), 135.3 (OCH$_2$CH*C*), 132.2 (*C*C≡CH), 130.6 (*C*HCC≡CH), 129.9 (OCH$_2$CHC*C*H), 129.4 (N*C*HCO), 129.3 (*C*HCHCC≡CH), 128.9 (OCH$_2$C*C*HCHCH), 128.6 (OCH$_2$CHCCH*C*H), 128.1 (OCH$_2$CCH*C*HCH), 127.7 (NCCH*C*H), 127.2 (OCH$_2$CHCCHCH*C*H), 122.4 (OCH$_2$C*C*HC), 74.1 (O*C*H$_2$PhCH$_2$), 73.1 (O*C*H$_2$CH), 70.3 (OCH$_2$*C*H), 69.0 (O*C*H$_2$PhCC≡H), 84.5 (*C*≡CH), 82.7 (C≡*C*H).

HRMS: Calculated for [Fe_2L_3]$^{4+}$ m/z 638.7313, found m/z 638.7264.

FTIR: υ cm^{-1} 3276 w, 3027 w, 2857 m, 1590 m, 1556 s, 1493 m, 1299 m, 1287 m, 1227 s, 1114 s, 1073 s, 1007 m, 823 m, 699 s, 531 m.

Elemental Analysis found (calculated for $C_{162}H_{138}Cl_4Fe_2N_{12}O_{12}\cdot 6H_2O$): % C 69.11 (69.33), H 5.64 (5.39), N 5.79 (5.99).

Λ_{Fe}-[$Fe_2L^{5g}{}_3$]Cl_4·$6H_2O$ (Λ_{Fe}-9g)

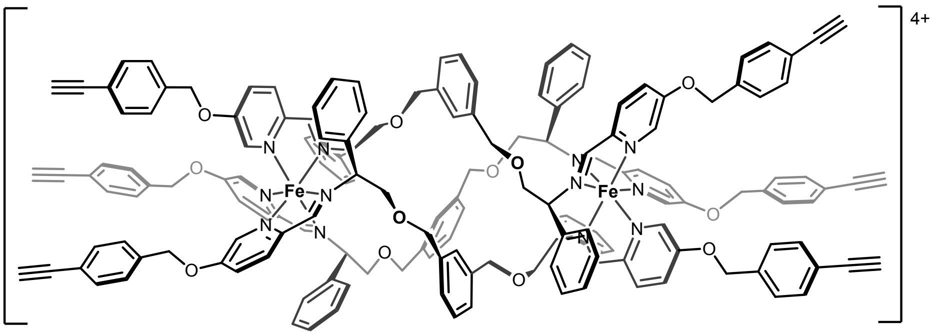

Λ_{Fe}-[Fe_2**L**$^{5g}{}_3$]$^{4+}$

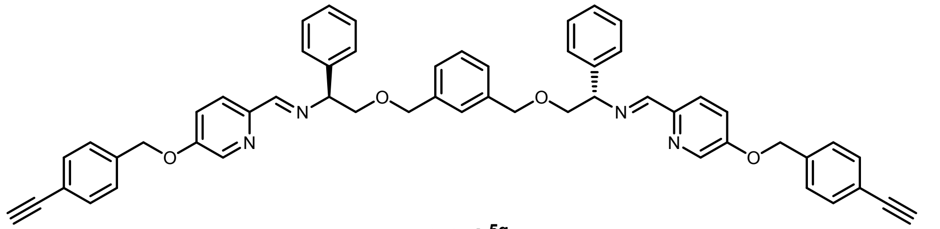

(*S*,*S*)-**L**^{5g}

0.21 g (0.56 mmol) of diamine used.

Yield: 0.46 g, 0.16 mmol, 88%.

^{1}H NMR (500 MHz, 298 K, MeOD): δ_H 9.02 (s, 6H, N=CH), 8.29 (s, 3H, OCH_2CCHC), 7.60 (d, $^3J_{HH}$ = 7.5 Hz, 6H, $OCH_2CCHCHCH$), 7.44 (m, 15H, $OCH_2CCHCHCH$, CHCC≡CH), 7.34 (d, $^3J_{HH}$ = 7.5 Hz, 12H, CHCHCC≡CH), 7.25-7.20 (m, 12H, CHNCCH), 7.00 (t, $^3J_{HH}$ = 7.5 Hz, 6H, NCCHCH), 6.82 (s, 12H, OCH_2CHCCH), 6.48 (s, 12H, $OCH_2CHCCCHCH$), 6.23 (s, 6H, $OCH_2CHCCHCHCH$), 5.66 (dd, $^3J_{HH}$ = 10.0, 2.5 Hz, 6H, OCH_2CH), 5.10 (s, 12H, OCH_2Ph-C≡CH), 4.94 (d, $^2J_{HH}$ = 11.0 Hz, 6H, OCH_2Ph), 4.79 (d, $^2J_{HH}$ = 11.0 Hz, 6H, OCH_2Ph), 4.28 (t, $^2J_{HH}$/$^3J_{HH}$ = 10.5 Hz, 6H, OCH_2CH), 3.67 (s, 6H, C≡CH), 2.96 (dd, $^3J_{HH}$ = 10.0, 2.5 Hz, 6H, OCH_2CH).

^{13}C{^{1}H} NMR (126 MHz, 298 K, MeOD): δ_C 171.6 (C=N), 155.9 (CC=N), 152.4 (NCHCO), 141.3 (CHCC=N), 139.6 ($COCH_2C$), 137.6 (CH_2OCH_2C), 135.3 (OCH_2CHC), 132.1 (CC≡CH), 130.6 (CHCC≡CH), 129.8 (OCH_2CHCCH), 129.4 (NCHCO), 129.3 (CHCHCC≡CH), 128.8 ($OCH_2CCHCHCH$), 128.6 ($OCH_2CHCCHCH$), 128.2 ($OCH_2CCHCHCH$), 127.7 (NCCHCH), 127.4 ($OCH_2CHCCHCHCH$), 122.2 (OCH_2CCHC), 74.4 (OCH_2PhCH_2), 73.1 (OCH_2CH), 70.3 (OCH_2CH), 69.2 (OCH_2PhCC≡H), 84.7 (C≡CH), 82.5 (C≡CH).

HRMS: Calculated for [Fe_2L_3]$^{4+}$ m/z 638.7314, found m/z 638.7275.

FTIR: υ cm^{-1} 3271 w, 3028 w, 2855 m, 1591 m, 1555 s, 1493 m, 1300 m, 1287 m, 1227 s, 1112 m, 1073 s, 1005 m, 821 m, 699 s, 530 m.

Elemental Analysis found (calculated for $C_{162}H_{138}Cl_4Fe_2N_{12}O_{12}\cdot 6H_2O$): % C 69.07 (69.33), H 5.49 (5.39), N 5.93 (5.99).

Δ_{Fe}-[$Fe_2L^{6e}{}_3$]Cl_4·11H_2O (Δ_{Fe}-10e)

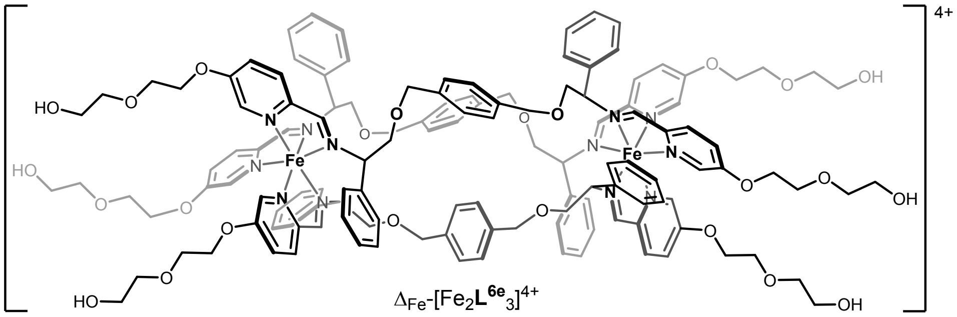

Δ_{Fe}-[Fe_2**L**$^{6e}{}_3$]$^{4+}$

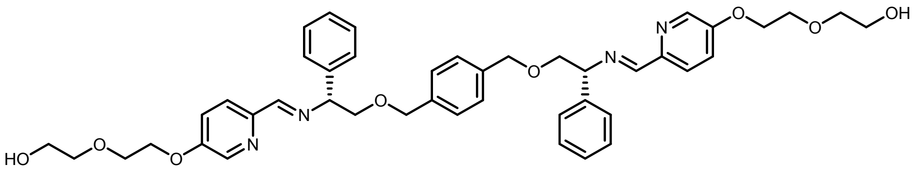

(R,R)-**L**^{6e}

0.22 g (5.8 mmol) of diamine used.

Yield: 0.42 g, 0.15 mmol, 80%.

^{1}H NMR (500 MHz, 298 K, MeOD): δ_H 9.18 (s, 6H, N=CH), 7.59-7.53 (m, 11H, Ar), 7.46 (s, 30H, Ar), 7.38 (s, 16H, Ar), 7.29 (s, 8H, Ar), 7.18-7.11 (m, 12H, Ar), 6.86 (d, $^3J_{HH}$ = 7.5 Hz, 12H, OCH$_2$CHCC*H*), 6.49 (s, 6H, C*H*NCC*H*), 5.86 (d, $^3J_{HH}$ = 10.5 Hz, 6H, OCH$_2$C*H*), 5.10 (d, $^3J_{HH}$ = 13.0 Hz, 12H, OC*H*$_2$Ph), 4.30 (t, $^2J_{HH}$/$^3J_{HH}$ = 10.5 Hz, 6H, OC*H*$_2$CH), 4.16 (s, 12H, PyOC*H*$_2$), 3.81-3.79 (m, 12H, PyOCH$_2$C*H*$_2$), 3.66-3.62 (m, $^3J_{HH}$ = 3.5 Hz, 12H, C*H*$_2$CH$_2$OH), 3.55 (s, 12H, CH$_2$C*H*$_2$OH). Presence of water (δ_H 5.04-4.75) obscures the second OC*H*$_2$Ph peak and residual MeOD solvent (δ_H 3.36-3.28) obscures the second OC*H*$_2$CH peak, however they can be detected by 2D-NMR (HSQC and HMBC).

^{13}C{1H} NMR (126 MHz, 298 K, MeOD): δ_C 170.3 (C=N), 157.9 (*C*C=N), 152.3 (NCH*C*O), 144.4 (*C*HCC=N), 137.1/134.6/130.1/129.5/128.3/128.1/127.9/127.8/ 127.5/127.1 (Ar), 123.9 (OCH$_2$C*C*H), 73.4 (O*C*H$_2$Ph), 72.8 (*C*H$_2$OH), 72.1 (O*C*H$_2$CH), 70.2 (OCH$_2$*C*H), 69.2 (PyOCH$_2$*C*H$_2$), 68.7 (PyO*C*H$_2$), 60.8 (*C*H$_2$CH$_2$OH).

HRMS: Calculated for [Fe_2L_3]$^{4+}$ m/z 599.7394, found m/z 599.7362.

FTIR: υ cm^{-1} 3331 w (br), 3030 w, 2858 w, 1592 m, 1558 m, 1494 m, 1452 m, 1305 m, 1233 m, 1074 s, 1045 s, 1019 m, 756 m, 698 s.

Elemental Analysis found (calculated for $C_{132}H_{150}Cl_4Fe_2N_{12}O_{24}$·11$H_2O$): % C 57.55 (57.86), H 6.43 (6.33), N 6.00 (6.13).

Λ$_{Fe}$-[$Fe_2L^{6e}{}_3$]Cl_4·12H_2O (Λ$_{Fe}$-10e)

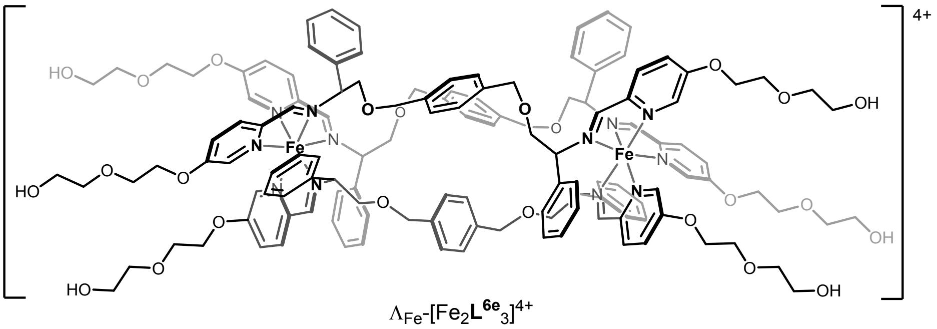

Λ$_{Fe}$-[Fe_2**L**$^{6e}{}_3$]$^{4+}$

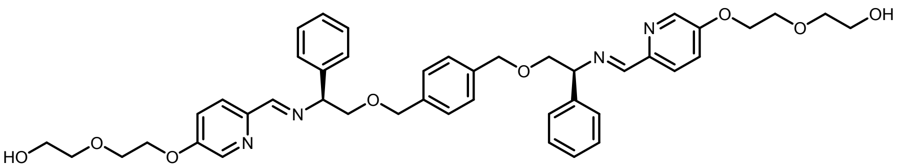

(*S*,*S*)-**L**^{6e}

0.20 g (0.53 mmol) of diamine used.

Yield: 0.42 g, 0.15 mmol, 87%.

^{1}H NMR (500 MHz, 298 K, MeOD): δ_H 9.20 (s, 6H, N=CH), 7.58-7.49 (m, 14H, Ar), 7.46 (s, 28H, Ar), 7.38 (s, 15H, Ar), 7.27 (s, 8H, Ar), 7.18-7.13 (m, 10H, Ar), 6.83 (d, $^3J_{HH}$ = 7.5 Hz, 12H, OCH$_2$CHCC*H*), 6.47 (s, 6H, C*H*NCC*H*), 5.86 (d, $^3J_{HH}$ = 10.0 Hz, 6H, OCH$_2$C*H*), 5.12 (d, $^3J_{HH}$ = 12.5 Hz, 12H, OC*H*$_2$Ph), 4.32 (t, $^2J_{HH}$/$^3J_{HH}$ = 10.5 Hz, 6H, OC*H*$_2$CH), 4.15 (s, 12H, PyOC*H*$_2$), 3.80-3.75 (m, 12H, PyOCH$_2$C*H*$_2$), 3.66-3.62 (m, $^3J_{HH}$ = 3.5 Hz, 12H, C*H*$_2$CH$_2$OH), 3.53 (s, 12H, CH$_2$C*H*$_2$OH). Presence of water (δ_H 5.06-4.80) obscures the second OC*H*$_2$Ph peak and residual MeOD solvent (δ_H 3.34-3.28) obscures the second OC*H*$_2$CH peak, however they can be detected by 2D-NMR (HSQC and HMBC).

^{13}C{1H} NMR (126 MHz, 298 K, MeOD): δ_C 170.4 (C=N), 157.6 (*C*C=N), 152.9 (NCH*C*O), 144.2 (*C*HCC=N), 137.4/134.7/129.9/128.6/128.2/127.5/127.2/127.0/126.7 (Ar), 123.6 (OCH$_2$C*C*H), 73.4 (O*C*H$_2$Ph), 72.6 (*C*H$_2$OH), 72.2 (O*C*H$_2$CH), 70.5 (OCH$_2$*C*H), 69.4 (PyOCH$_2$*C*H$_2$), 68.4 (PyO*C*H$_2$), 60.5 (*C*H$_2$CH$_2$OH).

HRMS: Calculated for [Fe_2L_3]$^{4+}$ m/z 599.7394, found m/z 599.7359.

FTIR: υ cm^{-1} 3330 w (br), 3027 w, 2857 w, 1591 m, 1556 m, 1495 m, 1452 m, 1303 m, 1233 m, 1072 s, 1047 s, 756 m, 699 s.

Elemental Analysis found (calculated for $C_{132}H_{150}Cl_4Fe_2N_{12}O_{24}$·12$H_2O$): % C 57.21 (57.48), H 6.51 (6.36), N 5.95 (6.09).

Δ_{Fe}-[$Fe_2L^{6f}{}_3$]Cl_4·$7H_2O$ (**Δ_{Fe}-10f**)

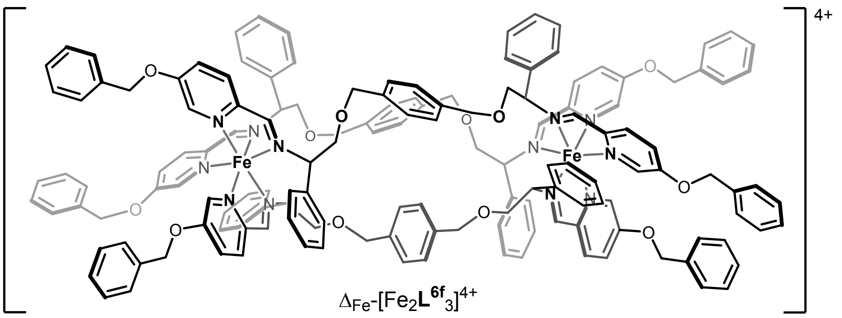

Δ_{Fe}-[Fe_2**L**$^{6f}{}_3$]$^{4+}$

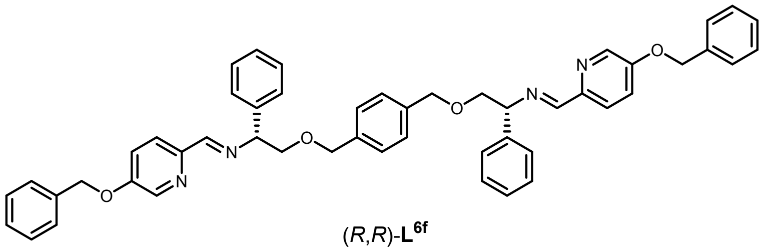

(*R*,*R*)-**L^{6f}**

0.21 g (0.56 mmol) of diamine used.

Yield: 0.42 g, 0.16 mmol, 84%.

^{1}H NMR (500 MHz, 298 K, MeOD): δ_H 9.04 (s, 6H, N=CH), 7.59-7.28 (m, 42H, OCH_2C*C*H*C*HC, OCH_2C*C*HCH*C*H, OCH_2CHCCHCH*CH*), 7.23 (t, $^3J_{HH}$ = 7.0 Hz, 6H, OCH_2CCH*CH*CH), 7.06 (t, $^3J_{HH}$ = 7.5 Hz, 12H, OCH_2CHCCH*CH*) 6.94 (t, $^3J_{HH}$ = 7.5 Hz, 12H, OCH_2CHCC*H*), 6.70 (d, $^3J_{HH}$ = 7.5 Hz, 12H, OCH_2CHCC*H*), 6.32 (s, 6H, C*H*NCC*H*), 5.76 (dd, $^3J_{HH}$ = 10.5, 2.0 Hz, 6H, OCH_2C*H*), 5.10 (q, $^4J_{HH}$ = 13.0 Hz, 12H, PyOC*H*$_2$Ph), 5.05 (d, $^2J_{HH}$ = 13.0 Hz, 6H, OC*H*$_2$Ph), 4.54 (d, $^2J_{HH}$ = 13.0 Hz, 6H, OC*H*$_2$Ph), 4.21 (t, $^2J_{HH}$/$^3J_{HH}$ = 11.0 Hz, 6H, OC*H*$_2$CH), 3.49 (dd, $^2J_{HH}$ = 11.0 Hz, $^3J_{HH}$ = 2.5 Hz, 6H, OC*H*$_2$CH).

^{13}C{^{1}H} NMR (126 MHz, 298 K, MeOD): δ_C 170.2 (C=N), 156.3 (*C*C=N), 151.9 (NCH*C*O), 144.0 (*C*HCC=N), 137.3 ($COCH_2$*C*), 135.6 ($CHOCH_2$*C*), 134.9 (OCH_2CH*C*), 129.8 (OCH_2CHC*C*H), 129.2 (N*C*HCO), 128.6 (OCH_2C*C*HCHCH), 128.5 (OCH_2CHCCH*C*H), 127.9 (OCH_2CCH*C*HCH), 127.8 (OCH_2CCHCH*C*H), 127.5 (NCCH*C*H), 126.9 (OCH_2CHCCHCH*C*H), 123.5 (OCH_2C*C*HCHC), 72.8 (O*C*H$_2$PhCH$_2$), 72.3 (O*C*H$_2$CH), 70.5 (OCH_2*C*H), 70.4 (O*C*H$_2$Ph).

HRMS: Calculated for [Fe_2L_3]$^{4+}$ m/z 602.7313, found m/z 602.7276.

FTIR: υ cm^{-1} 3333 w (br), 3028 w, 2859 w, 1590 m, 1556 s, 1493 m, 1452 m, 1227 s, 1108 m, 1076 m, 994 m, 739 m, 697 s.

Elemental Analysis found (calculated for $C_{150}H_{138}Cl_4Fe_2N_{12}O_{12}\cdot 8H_2O$): % C 66.63 (66.77), H 5.94 (5.75), N 6.07 (6.23).

Λ_{Fe}-[$Fe_2L^{6f}_3$]Cl_4·$8H_2O$ (Λ_{Fe}-10f)

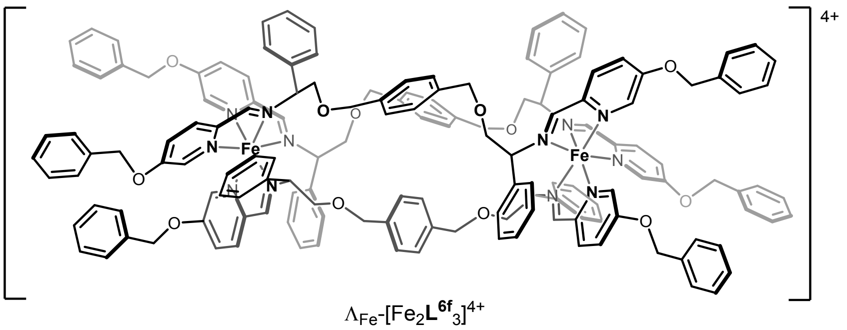

Λ_{Fe}-[Fe_2**L**$^{6f}_3$]$^{4+}$

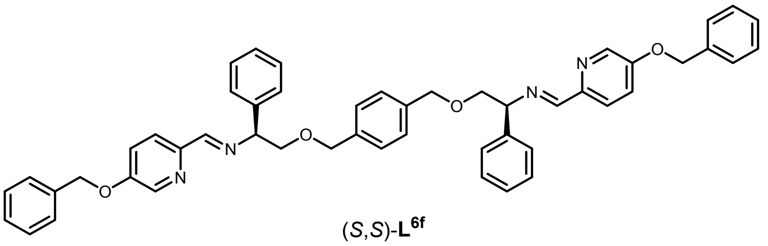

(S,S)-**L**^{6f}

0.21 g (5.6 mmol) of diamine used.

Yield: 0.40 g, 0.15 mmol, 80%.

^{1}H NMR (500 MHz, 298 K, MeOD): δ_H 9.03 (s, 6H, N=CH), 7.65-7.28 (m, 42H, OCH$_2$C*C*HC*H*C, OCH$_2$C*C*HCHC*H*, OCH$_2$CHCCHCHC*H*), 7.23 (d, $^3J_{HH}$ = 7.0 Hz, 6H, OCH$_2$CCHC*H*CH), 7.05 (t, $^3J_{HH}$ = 7.5 Hz, 12H, OCH$_2$CHCCHC*H*) 6.94 (t, $^3J_{HH}$ = 7.0 Hz, 12H, OCH$_2$CHCC*H*), 6.70 (d, $^3J_{HH}$ = 7.0 Hz, 12H, OCH$_2$CHCC*H*), 6.32 (s, 6H, C*H*NCC*H*), 5.76 (dd, $^3J_{HH}$ = 11.5, 2.0 Hz, 6H, OCH$_2$C*H*), 5.12 (q, $^4J_{HH}$ = 12.5 Hz, 12H, PyOC*H*$_2$Ph), 5.05 (d, $^2J_{HH}$ = 13.5 Hz, 6H, OC*H*$_2$Ph), 4.54 (d, $^2J_{HH}$ = 13.0 Hz, 6H, OC*H*$_2$Ph), 4.20 (t, $^2J_{HH}$/$^3J_{HH}$ = 11.0 Hz, 6H, OC*H*$_2$CH), 3.49 (dd, $^2J_{HH}$ = 11.0 Hz, $^3J_{HH}$ = 2.5 Hz, 6H, OC*H*$_2$CH).

^{13}C{^{1}H} NMR (126 MHz, 298 K, MeOD): δ_C 170.0 (C=N), 156.0 (*C*C=N), 151.7 (NCHC*O*), 144.0 (*C*HCC=N), 137.1 (COCH$_2$*C*), 135.2 (CHOCH$_2$*C*), 134.9 (OCH$_2$CHC*C*), 129.8 (OCH$_2$CHCCH), 129.1 (N*C*HCO), 128.7 (OCH$_2$C*C*HCHCH), 128.4 (OCH$_2$CHCCHCH), 127.8 (OCH$_2$CCH*C*HCH), 127.8 (OCH$_2$CCHCH*C*H), 127.4 (NCCH*C*H), 127.0 (OCH$_2$CHCCHCH*C*H), 123.4 (OCH$_2$C*C*HCHC), 72.7 (O*C*H$_2$PhCH$_2$), 72.2 (O*C*H$_2$CH), 70.5 (OCH$_2$*C*H), 70.3 (O*C*H$_2$Ph).

HRMS: Calculated for [Fe_2L_3]$^{4+}$ m/z 602.7313, found m/z 602.7274.

FTIR: υ cm^{-1} 3339 w (br), 3022 w, 2864 w, 1581 m, 1550 s, 1496 m, 1450 m, 1219 s, 1111 m, 1078 m, 990 m, 740 m, 695 s.

Elemental Analysis found (calculated for $C_{150}H_{138}Cl_4Fe_2N_{12}O_{12}$·$8H_2O$): % C 66.56 (66.77), H 6.01 (5.75), N 6.02 (6.23).

1.6 Synthesis and characterisation of [$^{56/57}Fe_2L_3$]Cl_4 triplexes

The amine (3.0 eq.) and corresponding pyridinecarboxaldehyde (3.0 eq.) were dissolved in methanol (25 ml) and stirred for 24 h at ambient temperature to form a yellow solution. Anhydrous $^{56/57}$Fe(II) chloride (2.0 eq.) was added and an instantaneous colour change to deep purple was observed. The solution was then heated at reflux (80 °C) for 48 h, filtered through a celite plug and then concentrated under reduced pressure. The crude was dissolved in minimum methanol (~2 ml) then pipetted into ethyl acetate (75 ml). The precipitate was filtered by fine filter paper, washed with ethyl acetate (3×25 ml), dissolved in methanol and the solvent was removed under reduced pressure to give the desired product as a purple solid, which was dried overnight at 50 °C *in vacuo.* Complexes were fully characterised, with ^{57}Fe compound spectra similar to the ^{56}Fe analogues.

Δ_{Fe}-[$Fe_2L^{7c}{}_3$]Cl_4·10H_2O (Δ_{Fe}-11c)

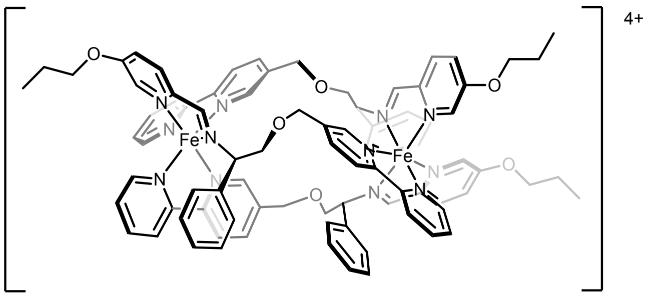

Δ_{Fe}-[Fe_2**L**$^{7c}{}_3$]$^{4+}$

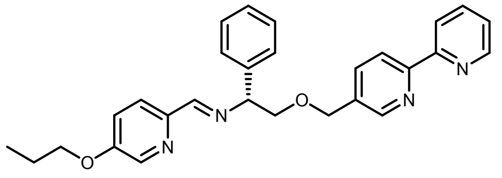

(*R*)-**L**^{7c}

0.07 g (0.23 mmol) of amine used.

Yield: 0.11 g, 0.06 mmol, 79%.

^{1}H NMR (400 MHz, 298 K, D_2O): δ_H 9.47 (s, 1H, N=CH), 9.37 (s, 1H, N=CH), 9.10 (s, 2H, bpy), 8.97 (s, 1H, N=CH), 8.37 (d, $^3J_{HH}$ = 8.0 Hz, 2H, Ar), 8.27 (d, $^3J_{HH}$ = 9.0 Hz, 1H, Ar), 8.20 (d, $^3J_{HH}$ = 9.0 Hz, 1H, Ar), 8.03-6.77 (m, 37H, Ar), 6.69 (s, 1H, Ar), 6.59 (t, $^3J_{HH}$ = 7.5 Hz, 2H, Ar), 6.48 (t, $^3J_{HH}$ = 7.5 Hz, 2H, Ar), 6.23 (s, 1H, Ar), 5.70 (s, 2H, Ar), 5.23 (d, $^3J_{HH}$ = 10.0 Hz, 1H, OCH_2CH), 5.14 (d, $^2J_{HH}$ = 13.0 Hz, 2H, OCH_2-bpy), 4.89 (d, $^2J_{HH}$ = 13.0 Hz, 1H, OCH_2-bpy), 4.51-3.99 (m, 11H, OCH_2-bpy/OCH_2CH), 3.87 (t, $^3J_{HH}$ = 6.0 Hz, 2H, $OCH_2CH_2CH_3$), 3.81 (t, $^3J_{HH}$ = 6.0 Hz, 2H, $OCH_2CH_2CH_3$), 3.69 (t, $^3J_{HH}$ = 6.0 Hz, 2H, $OCH_2CH_2CH_3$), 3.46 (d, $^3J_{HH}$ = 7.0 Hz, 1H, OCH_2CH), 3.29 (d, $^3J_{HH}$ = 10.0 Hz, 1H, OCH_2CH), 3.18 (d, $^3J_{HH}$ = 11.0 Hz, 1H, OCH_2CH), 1.58 (dd, $^3J_{HH}$ = 13.5 Hz, $^4J_{HH}$ = 7.0 Hz, 2H, $OCH_2CH_2CH_3$), 1.50 (dd, $^3J_{HH}$ = 13.5 Hz, $^4J_{HH}$ = 7.0 Hz, 2H, $OCH_2CH_2CH_3$), 1.40 (dd, $^3J_{HH}$ = 13.5 Hz, $^4J_{HH}$ = 7.0 Hz, 2H, $OCH_2CH_2CH_3$), 0.80 (t, $^3J_{HH}$ = 7.0 Hz, 3H, CH_3), 0.72 (t, $^3J_{HH}$ = 7.0 Hz, 3H, CH_3), 0.65 (t, $^3J_{HH}$ = 7.0 Hz, 3H, CH_3).

^{13}C {^{1}H} NMR (126 MHz, 298 K, D_2O): δ_C 170.2/169.9/169.3 (N=*C*H), 159.7/158.9/158.6/158.3/158.0/157.9/157.8/157.5/157.4/157.3/154.6/153.8/153.3/151.2/150.8/150.6/143.6/143.5/142.4/139.8/139.6/138.8/138.5/138.5/136.9/136.7/136.3/134.5/132.6/132.4/131.3/130.7/130.5/129.3/129.0/128.9/128.8/128.7/128.6/127.2/126.9/123.6/123.5/123.4/122.7/122.5/121.9/121.8/121.7/121.6 (Ar), 72.4/72.2 (CH_2*C*H), 71.1/71.0/70.9 (OCH_2), 70.2 (CH_2*C*H), 69.2/69.1/68.7 (*C*H_2-bpy), 68.5/68.4/67.9 (*C*H_2CH), 21.5/21.3/21.2 (*C*H_2CH_3), 9.41/9.37/9.23 (CH_3).

HRMS: Calculated for [Fe_2L_3]$^{4+}$ m/z 367.1330, found m/z 367.1319.

FTIR: υ cm^{-1} 3349 m (br), 2930 w, 1604 w, 1591 s, 1556 s, 1467 m, 1362 m, 1303 w, 1277 s, 1109 s, 1074 m, 1002 m, 964 m, 937 m, 841 m, 791 m, 754 m, 697 s.

Elemental Analysis found (Calculated for $C_{84}H_{84}Cl_4Fe_2N_{12}O_6 \cdot 10H_2O$) % C 55.68 (56.32), H 5.51 (5.85), N 9.01 (9.38).

Λ_{Fe}-[$Fe_2L^{7c}{}_3$]Cl_4·$10H_2O$ (Λ_{Fe}-11c)

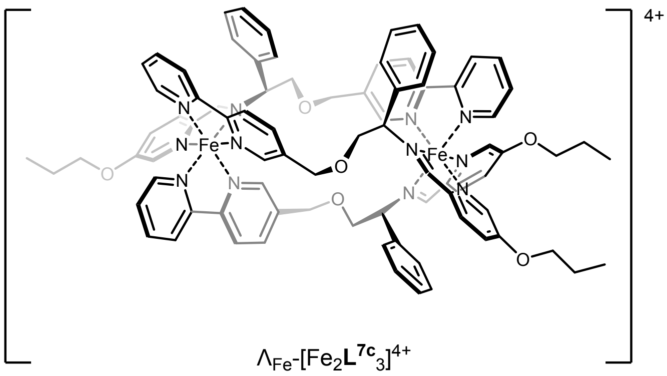

Λ_{Fe}-[Fe_2**L**$^{7c}{}_3$]$^{4+}$

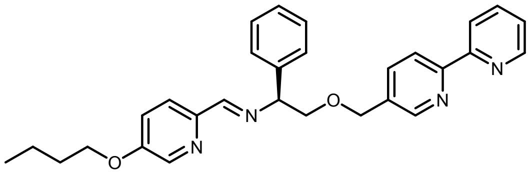

(S)-**L**^{7c}

0.07 g (0.23 mmol) of amine used.

Yield: 0.11 g, 0.07 mmol, 83%.

^{1}H NMR (400 MHz, 298 K, D_2O): δ_H 9.53 (s, 1H, N=CH), 9.43 (s, 1H, N=CH), 9.15 (s, 2H, bpy), 9.03 (s, 1H, N=CH), 8.43 (d, $^3J_{HH}$ = 6.0 Hz, 3H, Ar), 8.34 (d, $^3J_{HH}$ = 7.5 Hz, 2H, Ar), 8.27 (d, $^3J_{HH}$ = 9.0 Hz, 1H, Ar), 8.20 (d, $^3J_{HH}$ = 7.5 Hz, 1H, Ar), 8.10-6.80 (m, 37H, Ar), 6.71 (s, 1H, Ar), 6.64 (t, $^3J_{HH}$ = 7.5 Hz, 2H, Ar), 6.51 (t, $^3J_{HH}$ = 7.5 Hz, 2H, Ar), 6.28 (s, 1H, Ar), 5.76 (s, 2H, Ar), 5.28 (d, $^3J_{HH}$ = 8.5 Hz, 1H, OCH_2C*H*), 5.19 (d, $^2J_{HH}$ = 12.0 Hz, 2H, OC*H*$_2$-bpy), 4.94 (d, $^2J_{HH}$ = 12.5 Hz, 1H, OC*H*$_2$-bpy), 4.56-4.04 (m, 11H, OC*H*$_2$-bpy/OC*H*$_2$C*H*), 3.90-3.80 (m, 4H, OC*H*$_2CH_2CH_3$), 3.74 (t, $^3J_{HH}$ = 6.0 Hz, 2H, OC*H*$_2CH_2CH_3$), 3.51 (d, $^3J_{HH}$ = 7.0 Hz, 1H, OC*H*$_2$CH), 3.34 (d, $^3J_{HH}$ = 8.5 Hz, 1H, OC*H*$_2$CH), 3.18 (d, $^3J_{HH}$ = 11.0 Hz, 1H, OC*H*$_2$CH), 1.60 (dd, $^3J_{HH}$ = 13.5 Hz, $^4J_{HH}$ = 7.0 Hz, 2H, OCH_2C*H*$_2CH_3$), 1.50 (dd, $^3J_{HH}$ = 13.5 Hz, $^4J_{HH}$ = 7.0 Hz, 2H, OCH_2C*H*$_2CH_3$), 1.41 (dd, $^3J_{HH}$ = 13.0 Hz, $^4J_{HH}$ = 7.0 Hz, 2H, OCH_2C*H*$_2CH_3$), 0.80 (t, $^3J_{HH}$ = 7.0 Hz, 3H, CH_3), 0.73 (t, $^3J_{HH}$ = 7.0 Hz, 3H, CH_3), 0.68 (t, $^3J_{HH}$ = 7.0 Hz, 3H, CH_3).

^{13}C {^{1}H} NMR (126 MHz, 298 K, D_2O): δ_C 170.2/169.9/169.3 (N=*C*H), 159.7/158.9/158.6/158.3/158.0/157.9/157.9/157.5/157.4/157.3/154.6/153.8/153.3/151.2/150.8/150.6/143.6/142.4/139.8/139.6/138.8/138.5/136.9/136.7/136.3/134.5/132.6/132.4/131.3/130.7/130.5/129.3/128.9/128.8/128.6/127.2/126.9/123.6/123.5/123.4/122.7/122.5/121.8/121.7/121.6 (Ar), 72.4/72.2 (CH_2*C*H), 71.1/71.0/71.0 (OCH_2), 70.2 (CH_2*C*H), 69.2/69.1/68.7 (*C*H$_2$-bpy), 68.5/68.4/67.9 (*C*H$_2$CH), 21.4/21.3/21.2 (*C*H$_2CH_3$), 9.37/9.28/9.19 (CH_3).

HRMS: Calculated for [Fe_2L_3]$^{4+}$ m/z 367.1330, found m/z 367.1319.

FTIR: υ cm^{-1} 3349 m (br), 2929 w, 1604 w, 1591 s, 1555 s, 1467 m, 1362 m, 1300 w, 1277 s, 1111 s, 1074 m, 1003 m, 964 m, 937 m, 839 m, 791 m, 754 m, 698 s.

Elemental Analysis found (Calculated for $C_{84}H_{84}Cl_4Fe_2N_{12}O_6$·$10H_2O$) % C 56.28 (56.32), H 5.59 (5.85), N 9.15 (9.38).

Δ_{Fe}-[$Fe_2L^{7d}_3$]Cl_4·5H_2O (Δ_{Fe}-11d)

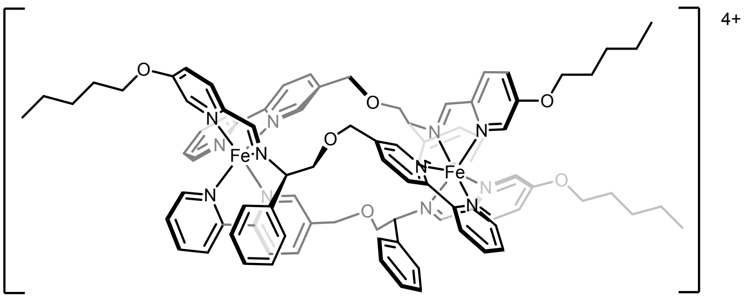

Δ_{Fe}-[Fe_2**L**$^{7d}_3$]$^{4+}$

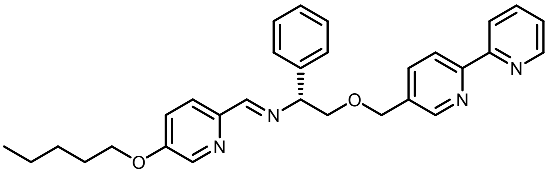

(*R*)-**L**^{7d}

0.07 g (0.23 mmol) of amine used.

Yield: 0.09 g, 0.06 mmol, 76%.

^{1}H NMR (500 MHz, 298 K, D_2O): δ_H 9.52 (s, 1H, N=CH), 9.43 (s, 1H, N=CH), 9.14 (s, 2H, bpy), 9.04 (s, 1H, N=CH), 8.43 (d, $^3J_{HH}$ = 8.0 Hz, 2H, Ar), 8.32 (d, $^3J_{HH}$ = 9.0 Hz, 1H, Ar), 8.20 (d, $^3J_{HH}$ = 9.0 Hz, 1H, Ar), 8.05-7.95 (m, 2H, bpy), 7.92-7.45 (m, 15H, Ar), 7.31 (t, $^3J_{HH}$ = 10.0 Hz, 1H, Ar), 7.27-7.02 (m, 10H, Ar), 6.97 (t, $^3J_{HH}$ = 7.5 Hz, 1H, Ar), 6.93-6.78 (m, 5H, Ar), 6.71 (s, 1H, Ar), 6.64 (t, $^3J_{HH}$ = 7.5 Hz, 2H, Ar), 6.53 (t, $^3J_{HH}$ = 7.5 Hz, 2H, Ar), 6.24 (s, 1H, Ar), 5.74 (s, 2H, Ar), 5.28 (d, $^2J_{HH}$ = 8.5 Hz, 1H, OCH_2C*H*), 5.18 (d, $^2J_{HH}$ = 13.0 Hz, 2H, O*C*H_2-bpy), 4.93 (d, $^2J_{HH}$= 13.0 Hz, 1H, OC*H*$_2$-bpy), 4.53-4.25 (m, 7H, OC*H*$_2$-bpy/OC*H*$_2$C*H*), 4.19 (t, $^3J_{HH}$ = 11.0 Hz, 1H, OC*H*$_2$CH), 4.00-3.73 (m, 6H, OC*H*$_2$(CH_2)$_3$$CH_3$), 3.52 (d, $^3J_{HH}$ = 7.0 Hz, 1H, OC*H*$_2$CH), 3.33 (d, $^3J_{HH}$ = 10.0 Hz, 1H, OC*H*$_2$CH), 3.23 (dd, $^3J_{HH}$ = 9.5 Hz, 1H, OC*H*$_2$CH), 1.63-1.33 (m, 6H, (C*H*$_2$)$_3$$CH_3$), 1.27-1.01 (m, 6H, (C*H*$_2$)$_3$$CH_3$), 0.77 (t, $^3J_{HH}$ = 6.5 Hz, 3H, CH_3), 0.72 (t, $^3J_{HH}$ = 6.5 Hz, 3H, CH_3), 0.68 (t, $^3J_{HH}$ = 6.5 Hz, H, CH_3).

^{13}C {^{1}H} NMR (126 MHz, 298 K, D_2O): δ_C 170.1/169.9/169.4 (N=CH), 159.7/158.8/158.5/ 158.3/158.0/157.9//157.8/157.7/157.5/157.4/157.3/154.6/153.8/153.3/153.2/151.3/150.9/ 150.8/143.3/143.1/142.1/139.8/139.6/138.8/138.6/138.5/136.9/136.7/136.3/134.0/132.6/ 132.3/131.2/130.8/130.6/129.0/128.9/128.8/128.7/128.6/127.2/126.9/123.6/123.5/123.4/ 122.7/122.5/122.3/122.2/122.1/121.9 (Ar), 72.4/72.3/70.2 (OCH_2*C*H), 69.7/69.6/69.5 (O*C*H_2(CH_2)$_3$$CH_3$), 69.2/69.1/68.7 (*C*$H_2$-bpy), 68.5/68.4/67.9 (O*C*H_2CH), 27.6/27.4/27.3/ 27.2/27.0/26.9/21.7/21.6/21.5 ((*C*H_2)$_3$$CH_3$), 13.2/13.1/13.1 ($CH_3$).

HRMS: Calculated for [Fe_2L_3]$^{4+}$ m/z 388.1565, found m/z 388.1531.

FTIR: υ cm^{-1} 3350 m (br), 2928 w, 1604 m, 1590 m, 1555 s, 1467 m, 1440 m, 1402 w, 1375 m, 1304 w, 1278 m, 1234 s, 1109 m, 1074 s, 978 m, 938 m, 841 m, 792 m, 755 m, 698 s.

Elemental Analysis found (Calculated for $C_{90}H_{96}Cl_4Fe_2N_{12}O_6$·5$H_2O$): % C 60.30 (60.55), H 5.82 (5.98), N 9.28 (9.41).

Λ_{Fe}-[$Fe_2L^{7d}{}_3$]Cl_4·5H_2O (Λ_{Fe}-11d)

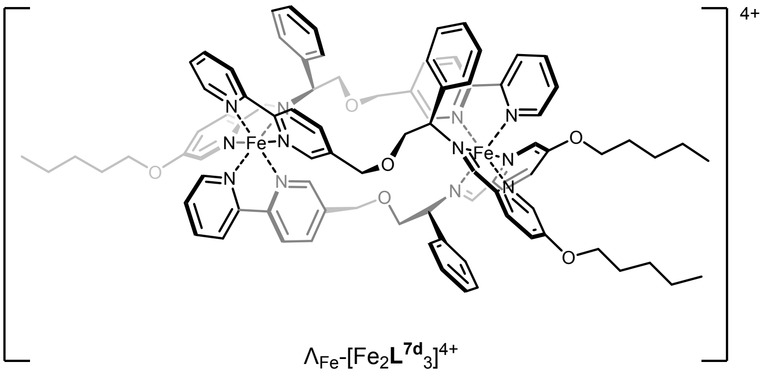

Λ_{Fe}-[Fe_2**L**$^{7d}{}_3$]$^{4+}$

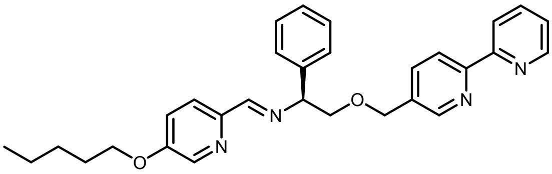

(*S*)-**L**^{7d}

0.07 g (0.23 mmol) of amine used.

Yield: 0.10 g, 0.06 mmol, 80%.

^{1}H NMR (500 MHz, 298 K, D_2O): δ_H 9.52 (s, 1H, N=CH), 9.43 (s, 1H, N=CH), 9.14 (s, 2H, bpy), 9.04 (s, 1H, N=CH), 8.43 (d, $^3J_{HH}$ = 8.0 Hz, 2H, Ar), 8.32 (d, $^3J_{HH}$ = 9.0 Hz, 1H, Ar), 8.20 (d, $^3J_{HH}$ = 9.0 Hz, 1H, Ar), 8.05-7.95 (m, 2H, bpy), 7.92-7.45 (m, 15H, Ar), 7.31 (t, $^3J_{HH}$ = 10.0 Hz, 1H, Ar), 7.27-7.02 (m, 10H, Ar), 6.97 (t, $^3J_{HH}$ = 7.5 Hz, 1H, Ar), 6.93-6.78 (m, 5H, Ar), 6.71 (s, 1H, Ar), 6.64 (t, $^3J_{HH}$ = 7.5 Hz, 2H, Ar), 6.53 (t, $^3J_{HH}$ = 7.5 Hz, 2H, Ar), 6.24 (s, 1H, Ar), 5.74 (s, 2H, Ar), 5.28 (d, $^2J_{HH}$ = 8.5 Hz, 1H, OCH_2CH), 5.18 (d, $^2J_{HH}$ = 13.0 Hz, 2H, OCH_2-bpy), 4.93 (d, $^2J_{HH}$= 13.0 Hz, 1H, OCH_2-bpy), 4.53-4.25 (m, 7H, OCH_2-bpy/OCH_2CH), 4.19 (t, $^3J_{HH}$ = 11.0 Hz, 1H, OCH_2CH), 4.00-3.73 (m, 6H, $OCH_2(CH_2)_3CH_3$), 3.52 (d, $^3J_{HH}$ = 7.0 Hz, 1H, OCH_2CH), 3.33 (d, $^3J_{HH}$ = 10.0 Hz, 1H, OCH_2CH), 3.23 (dd, $^3J_{HH}$ = 9.5 Hz, 1H, OCH_2CH), 1.63-1.33 (m, 6H, $(CH_2)_3CH_3$), 1.27-1.01 (m, 6H, $(CH_2)_3CH_3$), 0.77 (t, $^3J_{HH}$ = 6.5 Hz, 3H, CH_3), 0.72 (t, $^3J_{HH}$ = 6.5 Hz, 3H, CH_3), 0.68 (t, $^3J_{HH}$ = 6.5 Hz, 3H, CH_3).

^{13}C {^{1}H} NMR (126 MHz, 298 K, D_2O): δ_C 170.0/169.8/169.2 (N=CH), 159.8/158.8/158.5/158.3/158.1/157.9//157.8/157.7/157.5/157.3/157.1/154.6/153.8/153.3/153.2/151.3/151.0/150.8/143.3/143.1/142.2/139.8/139.6/138.8/138.6/138.4/136.9/136.7/136.3/134.1/132.6/132.3/131.2/130.7/130.6/129.2/128.9/128.8/128.7/128.5/127.2/126.9/123.6/123.5/123.4/122.7/122.5/122.4/122.3/122.2/121.9 (Ar), 72.4/72.3/70.2 (OCH_2CH), 69.7/69.6/69.6 ($OCH_2(CH_2)_3CH_3$), 69.2/69.1/68.8 (CH_2-bpy), 68.5/68.4/67.8 (OCH_2CH), 27.6/27.5/27.3/ 27.2/27.0/26.9/21.7/21.7/21.5 ($(CH_2)_3CH_3$), 13.2/13.1/13.1 (CH_3).

HRMS: Calculated for [Fe_2L_3]$^{4+}$ m/z 388.1577, found m/z 388.1564.

FTIR: υ cm^{-1} 3349 m (br), 2928 w, 1604 m, 1590 m, 1554 s, 1467 m, 1440 m, 1402 w, 1375 m, 1304 w, 1276 m, 1234 s, 1109 m, 1074 s, 980 m, 938 m, 841 m, 791 m, 755 m, 699 s.

Elemental Analysis found (Calculated for $C_{90}H_{96}Cl_4Fe_2N_{12}O_6$·5$H_2O$): % C 60.71 (60.55), H 5.86 (5.98), N 9.16 (9.41).

Δ_{Fe}-[$Fe_2L^{7e}{}_3$]$Cl_4\cdot 8H_2O$ (Δ_{Fe}-11e)

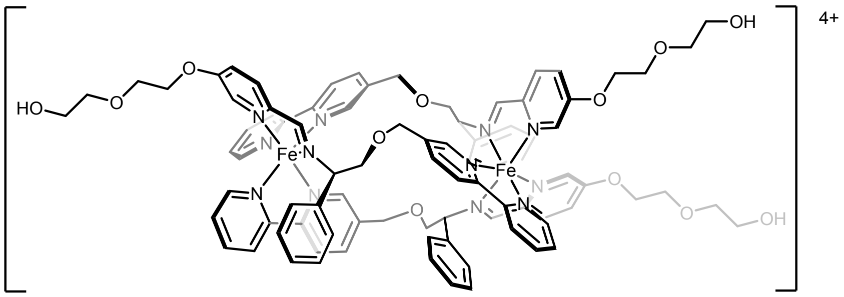

Δ_{Fe}-[Fe_2**L**$^{7e}{}_3$]$^{4+}$

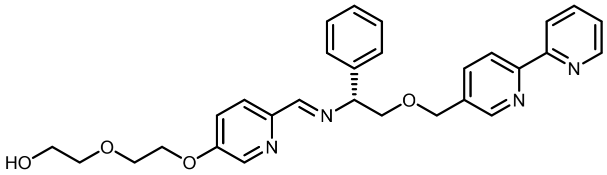

(*R*)-**L**^{7e}

0.10 g (0.33 mmol) of amine used.

Yield: 0.19 g, 0.10 mmol, 94%.

$^{13}C\{^1H\}$ NMR (126 MHz, 298 K, MeOD): δ_C 153.6/151.8/150.8/140.5/138.5/135.1/ 134.8/134.2/133.6/132.7/132.1/130.8/130.1/128.4/127.7/127.6/126.8/126.2/125.3/ 124.5/122.9/111.2 (Ar), 78.5/78.4/77.8/77.5/77.2/77.1/76.7/76.5/76.3/76.1/75.7/75.5/ 75.0/74.8/74.3 (CH_2), 74.0 (CH), 73.7 (CH_2), 73.6/73.5 (CH), 73.1/72.7 (CH_2).

HRMS: Calculated for [Fe_2L_3]$^{4+}$ m/z 401.6378, found m/z 401.6362.

FTIR: υ cm^{-1} 3357 w (br), 2859 m (br), 1602 m, 1509 m, 1497 m, 1466 s, 1240 m, 1095 s, 840 m, 785 m, 753 m, 730 m, 698 m, 670 m.

Elemental Analysis found (calculated for $C_{87}H_{90}Cl_4Fe_2N_{12}O_{12}\cdot 8H_2O$): % C 54.98 (55.19), H 5.78 (5.64), N 8.65 (8.88).

Λ_{Fe}-[$Fe_2L^{7e}{}_3$]Cl_4·$9H_2O$ (Λ_{Fe}-11e)

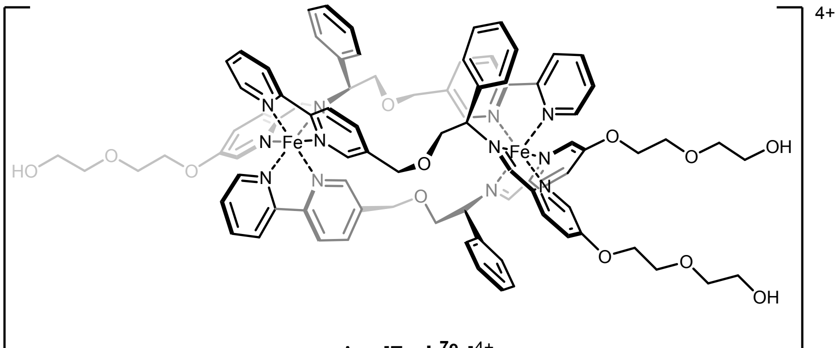

Λ_{Fe}-[Fe_2**L**$^{7e}{}_3$]$^{4+}$

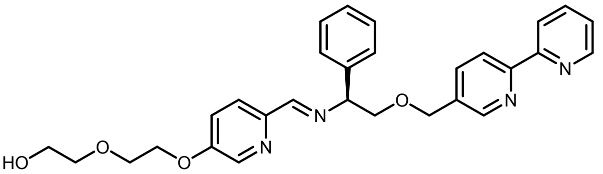

(*S*)-**L**^{7e}

0.10 g (0.33 mmol) of amine used.

Yield: 0.20 g, 0.10 mmol, 96%.

$^{13}C\{^1H\}$ NMR (126 MHz, 298 K, MeOD): δ_C 153.9/151.4/142.6/138.9/135.4/135.2/ 134.7/133.8/133.2/ 132.5/132.2/130.7/130.5/130.0/128.1/127.6/127.2/126.5/125.8/124.7/122.7/111.4/105.4 (Ar), 78.9/78.3/77.7/77.6/77.2/77.1/76.8/76.6/76.4/76.1/ 75.9/75.6/75.2/75.0/74.8/74.1 (CH_2), 73.8 (CH), 73.6 (CH_2), 73.4/73.3 (CH), 73.0/72.8 (CH_2).

HRMS: Calculated for [Fe_2L_3]$^{4+}$ m/z 401.6378, found m/z 401.6364.

FTIR: υ cm^{-1} 3362 w (br), 2855 m (br), 1603 m, 1509 m, 1498 m, 1467 s, 1241 m, 1094 s, 841 m, 787 m, 751 m, 728 m, 698 m, 671 m.

Elemental Analysis found (calculated for $C_{87}H_{90}Cl_4Fe_2N_{12}O_{12}\cdot8H_2O$): % C 55.28 (55.19), H 5.78 (5.77), N 8.65 (8.77).

Δ_{Fe}-[$Fe_2L^{7g}_3$]Cl_4·$10H_2O$ (Δ_{Fe}-11g)

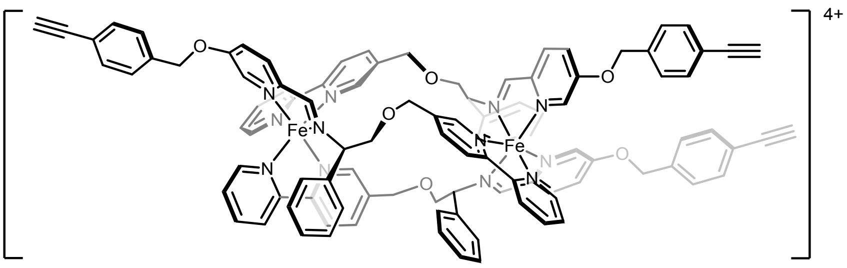

Δ_{Fe}-[Fe_2**L**$^{7g}{}_3$]$^{4+}$

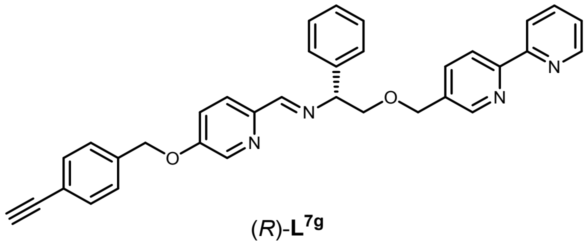

(*R*)-**L^{7g}**

0.15 g (0.49 mmol) of amine used.

Yield: 0.29 g, 0.14 mmol, 87%.

$^{13}C\{^1H\}$ NMR (126 MHz, 298 K, MeOD): δ_C 171.1/171.1/170.4 (N=CH), 159.8/159.8/ 159.7/158.2/157.8/ 157.6/157.5/157.4/157.2/153.9/152.9/151.8/151.6/151.3/142.9/139.9/139.8/139.0/138.5/137.4/137.1/ 136.9/135.7/135.4/135.3/134.5/132.9/132.6/132.2/132.1/132.0/131.8/131.2/130.6/128.9/128.8/128.6/ 128.5/127.6/127.5/127.4/127.2/124.0/123.6/123.4/123.2/122.9/122.8/122.7/122.6/122.0 (Ar), 82.3/81.9/78.5/78.4/70.3/70.2/70.1/69.2/ 69.1/68.9/68.6/68.4 (CH_2, C≡CH, C≡CH), 72.4/72.3/70.3 (CH_2CH).

HRMS: Calculated for [Fe_2L_3]$^{4+}$ m/z 421.1331, found m/z 421.1296.

FTIR: υ cm^{-1} 3261 w (br), 3031 w, 2867 m, 1556 m, 1467 s, 1231 m, 1071 s, 1055 s, 1006 s, 935 m, 823 m, 788 m, 752 m, 696 m, 671 m, 441 m.

Elemental Analysis found (calculated for $C_{102}H_{84}Cl_4Fe_2N_{12}O_6$·$10H_2O$): % C 61.01 (61.03), H 5.55 (5.22), N 8.24 (8.37).

Λ_{Fe}-[$Fe_2L^{7g}{}_3$]Cl_4·10H_2O (Λ_{Fe}-11g)

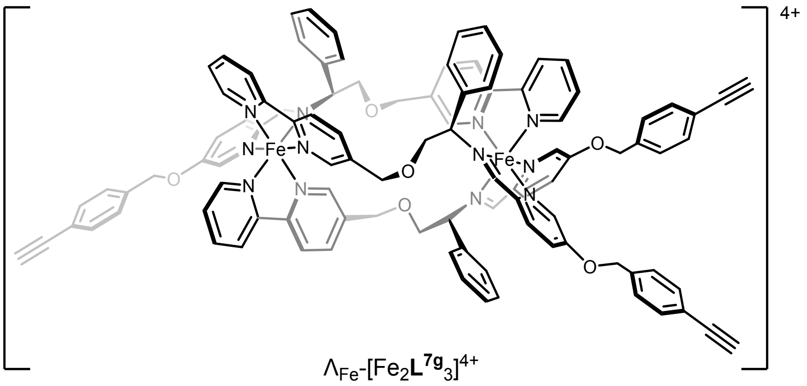

Λ_{Fe}-[Fe_2**L**$^{7g}{}_3$]$^{4+}$

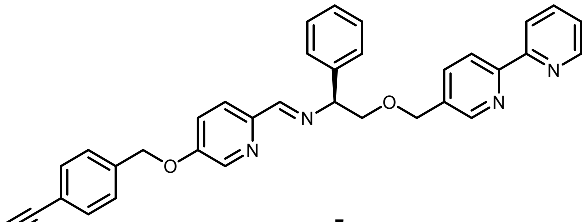

(*S*)-**L**^{7g}

0.15 g (0.49 mmol) of amine used.

Yield: 0.31 g, 0.15 mmol, 94%.

$^{13}C\{^1H\}$ NMR (126 MHz, 298 K, MeOD): δ_C 171.3/171.2/170.4 (N=CH), 159.9/159.7/ 158.4/157.7/157.6/ 157.5/157.3/157.2/153.7/152.9/151.9/151.7/151.0/142.7/142.5/139.9/139.5/139.0/138.8/137.4/137.1/ 136.8/135.7/135.6/135.3/134.6/132.9/132.4/132.3/132.1/132.0/131.7/131.2/130.9/128.9/128.6/128.4/ 127.7/127.5/127.4/127.0/124.2/123.7/123.4/123.2/122.9/122.8/122.6/122.4/122.0 (Ar), 82.6/81.6/78.6/78.4/70.3/70.1/69.5/69.2/68.8/68.6/ 68.3 (CH_2, C≡CH, C≡CH), 72.6/72.3/70.4 (CH_2CH).

HRMS: Calculated for [Fe_2L_3]$^{4+}$ m/z 421.1331, found m/z 421.1320.

FTIR: ʋ cm^{-1} 3267 w (br), 3029 w, 2869 m, 1554 m, 1467 s, 1230 m, 1070 s, 1054 s, 1002 s, 935 m, 821 m, 786 m, 750 m, 698 m, 668 m, 442 m.

Elemental Analysis found (calculated for $C_{102}H_{84}Cl_4Fe_2N_{12}O_6$·10$H_2O$): % C 60.78 (61.03), H 5.52 (5.22), N 8.00 (8.37).

1.7 Synthesis and characterisation of [Zn_2L_3][ClO_4]$_4$ flexicates

The diamine (3.0 eq.) and corresponding pyridinecarboxaldehyde (6.0 eq.) were dissolved in acetonitrile (25 ml) and stirred for 24 h at ambient temperature to form a yellow solution. Anhydrous zinc(II) perchlorate (2.0 eq.) was added and stirred at ambient temperature for 24 h, then concentrated under reduced pressure. The crude was dissolved in minimum acetonitrile (~1 ml) then pipetted into ethyl acetate (75 ml). The precipitate was filtered by fine filter paper, washed with ethyl acetate (3×25 ml), dissolved in acetonitrile and the solvent was removed under reduced pressure to give the desired product as a yellow solid, which was dried overnight at ambient temperature *in vacuo.*

Δ_{Zn}-[$Zn_2L^{5f}{}_3$][ClO_4]$_4$ (Δ_{Zn}-9f)

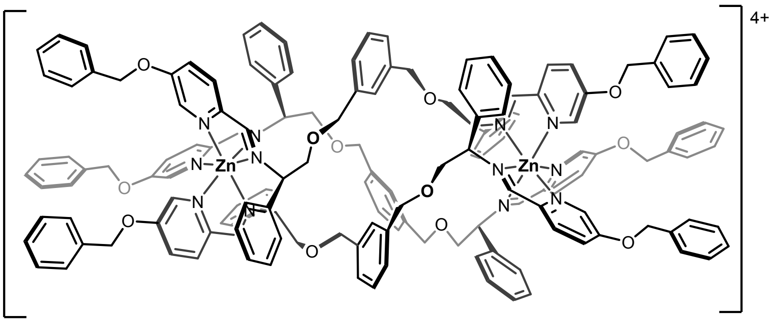

Δ_{Zn}-[Zn_2**L**$^{5f}{}_3$]$^{4+}$

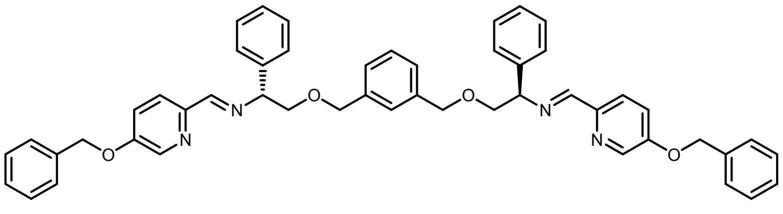

(R,R)-**L**^{5f}

0.07 g (0.18 mmol) of diamine used.

Yield: 0.13 g, 0.05 mmol, 78%.

^{1}H NMR (500 MHz, 298 K, CD_3CN): δ_H 8.35 (s, 6H, N=CH), 8.13 (s, 3H, OCH_2CCHC), 7.50-7.28 (m, 18H, Ar), 7.27-7.08 (m, 33H, Ar), 7.03 (d, $^3J_{HH}$ = 8.5 Hz, 6H, Ar), 6.82 (t, $^3J_{HH}$ = 7.5 Hz, 6H, Ph), 6.61 (t, $^3J_{HH}$ = 7.5 Hz, 12H, Ph), 6.16 (d, $^3J_{HH}$ = 7.5 Hz, 12H, Ph), 5.39 (dd, $^3J_{HH}$ = 11.5, 2.5 Hz, 6H, OCH_2CH), 5.03 (d, $^4J_{HH}$ = 3.0 Hz, 12H, OCH_2Ph), 4.85 (d, $^2J_{HH}$ = 11.0 Hz, 6H, OCH_2PhCH_2), 4.68 (d, $^2J_{HH}$ = 11.0 Hz, 6H, OCH_2PhCH_2), 3.91 (t, $^2J_{HH}/^3J_{HH}$ = 11.5 Hz, 6H, OCH_2CH), 2.99 (dd, $^2J_{HH}$ = 11.5 Hz, $^3J_{HH}$ = 3.0 Hz, 6H, OCH_2CH).

$^{13}C\{^1H\}$ NMR (126 MHz, 298 K, CD_3CN): δ_C 162.1 (C=N), 158.9 (CC=N), 140.1/139.3/ 138.3/136.1/135.9/131.7/130.0/129.8/129.7/129.5/129.3/129.0/128.6/128.0 (Ar), 127.0 (Ph), 125.8 (Ar), 75.1 (OCH_2PhCH_2), 73.0 (OCH_2CH), 71.5 (OCH_2Ph), 67.2 (OCH_2CH).

Λ_{Zn}-[$Zn_2L^{5f}{}_3$][ClO_4]$_4$ (Λ_{Zn}-9f)

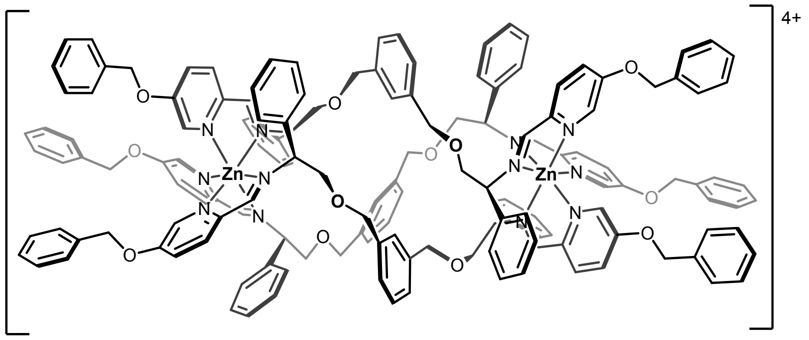

Λ_{Zn}-[Zn_2**L**$^{5f}{}_3$]$^{4+}$

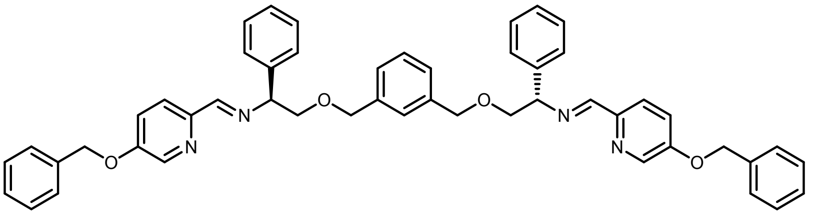

(S,S)-**L**^{5f}

0.07 g (0.18 mmol) of diamine used.

Yield: 0.12 g, 0.04 mmol, 75%.

^{1}H NMR (500 MHz, 298 K, CD_3CN): δ_H 8.35 (s, 6H, N=CH), 8.12 (s, 3H, OCH_2CCHC), 7.51-7.27 (m, 18H, Ar), 7.27-6.98 (m, 39H, Ar), 6.80 (t, $^{3}J_{HH}$ = 7.5 Hz, 6H, Ph), 6.59 (t, $^{3}J_{HH}$ = 7.5 Hz, 12H, Ph), 6.14 (d, $^{3}J_{HH}$ = 8.0 Hz, 12H, Ph), 5.37 (d, $^{3}J_{HH}$ = 9.5 Hz, 6H, $OCH_2C\mathit{H}$), 5.01 (s, 12H, $OC\mathit{H}_2Ph$), 4.83 (d, $^{2}J_{HH}$ = 11.0 Hz, 6H, $OC\mathit{H}_2PhCH_2$), 4.67 (d, $^{2}J_{HH}$ = 10.5 Hz, 6H, $OC\mathit{H}_2PhCH_2$), 3.90 (t, $^{2}J_{HH}$/$^{3}J_{HH}$ = 11.5 Hz, 6H, $OC\mathit{H}_2CH$), 2.97 (d, $^{2}J_{HH}$ = 9.5 Hz, 6H, $OC\mathit{H}_2CH$).

^{13}C{^{1}H} NMR (126 MHz, 298 K, CD_3CN): δ_C 162.1 (C=N), 158.8 (CC=N), 140.3/139.3/138.3/136.3/136.0/131.9/130.2/129.8/129.7/129.4/129.2/128.8/128.4/127.9 (Ar), 127.1 (Ph), 125.8 (Ar), 75.0 (OCH_2PhCH_2), 72.8 (OCH_2CH), 71.4 (OCH_2Ph), 67.3 (OCH_2CH).

1.8 Synthesis and characterisation of [Zn_2L_3][ClO_4]$_4$ triplexes

The amine (3.0 eq.) and corresponding pyridinecarboxaldehyde (3.0 eq.) were dissolved in acetonitrile (25 ml) and stirred for 24 h at ambient temperature to form a yellow solution. Anhydrous zinc(II) perchlorate (2.0 eq.) was added and a colour change to orange was observed. The solution was then stirred at ambient temperature for 24 h, then concentrated under reduced pressure. The crude was dissolved in minimum acetonitrile (~1 ml) then pipetted into ethyl acetate (75 ml). The precipitate was filtered by fine filter paper, washed with ethyl acetate (3×25 ml), dissolved in acetonitrile and the solvent was removed under reduced pressure to give the desired product as a yellow solid, which was dried overnight at ambient temperature *in vacuo.*

Δ_{Zn}-[$Zn_2L^{7e}{}_3$][ClO_4]$_4$ (**Δ_{Zn}-11e**)

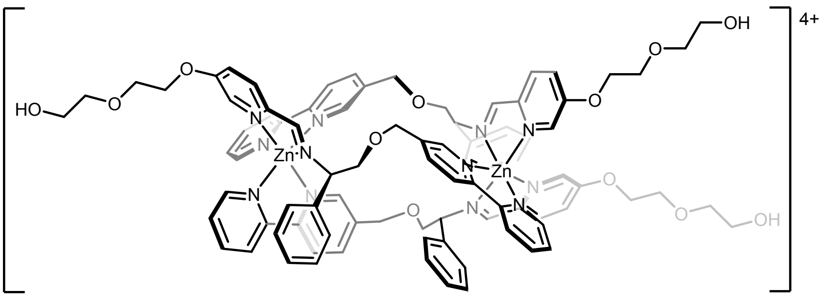

Δ_{Zn}-[Zn_2**L**$^{7e}{}_3$]$^{4+}$

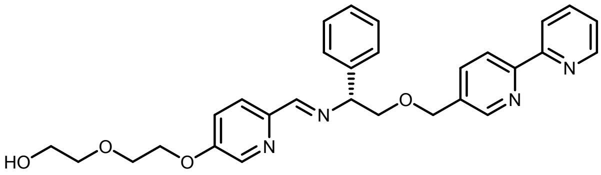

(R)-**L^{7e}**

0.04 g (0.13 mmol) of amine used.

Yield: 0.08 g, 0.04 mmol, 92%.

^{1}H NMR (500 MHz, 298 K, CD_3CN): δ_H 9.22 (s, 1H, N=CH), 9.19 (d, $^3J_{HH}$ = 3.0 Hz, 2H, NC*H*CCH$_2$), 9.12 (s, 1H, NC*H*CCH$_2$), 9.10 (s, 1H, N=CH), 8.73 (s, 1H, N=CH), 8.50 (d, $^3J_{HH}$ = 8.0 Hz, 1H, Ar), 8.45 (d, $^3J_{HH}$ = 6.5 Hz, 1H, Ar), 8.40 (s, 1H, Ar), 8.34 (s, 1H, NC*H*CCH$_2$), 8.29 (m, 7H, Ar), 8.15 (d, $^3J_{HH}$ = 8.5 Hz, 2H), 8.08-7.79 (m, 23H, Ar), 7.79-7.73 (m, 3H, Ar), 7.65-7.56 (m, 2H, Ar), 7.46 (m, 12H, Ar), 7.30-7.16 (m, 10H, Ar), 7.11 (m, 3H, Ar), 7.03 (s, 2H, Ar), 6.99-6.81 (m, 15H, Ar), 6.69 (t, $^3J_{HH}$ = 7.0 Hz, 2H, OCH$_2$CHCCHC*H*), 6.56 (t, $^3J_{HH}$ = 7.5 Hz, 2H, OCH$_2$CHCCHC*H*), 6.08 (d, $^3J_{HH}$ = 3.5 Hz, 2H, OCH$_2$CHCC*H*), 5.95 (d, $^3J_{HH}$ = 3.5 Hz, 2H, OCH$_2$CHCC*H*), 5.41 (dd, $^3J_{HH}$ = 11.5, 3.5 Hz, 1H, OCH$_2$C*H*), 5.20 (d, $^3J_{HH}$ = 2.5 Hz, 1H, bpy-C*H*$_2$), 4.93 (dd, $^3J_{HH}$ = 11.0, 2.5 Hz, 1H, OCH$_2$C*H*), 4.78 (dd, $^3J_{HH}$ = 12.0, 3.5 Hz, 2H, OCH$_2$C*H*), 4.53 (d, $^3J_{HH}$ = 2.0 Hz, 2H, bpy-C*H*$_2$), 4.50 (d, $^3J_{HH}$ = 2.5 Hz, 2H, bpy-C*H*$_2$), 4.31-4.23 (m, 1H, OC*H*$_2$CH), 4.13 (t, $^3J_{HH}$ = 11.5 Hz, 1H, OC*H*$_2$CH), 4.09-3.96 (m, 7H, OC*H*$_2$CH, PyOC*H*$_2$), 3.75-3.39 (m, 21H, OC*H*$_2$CH, C*H*$_2$OC*H*$_2$C*H*$_2$OH).

Δ_{Zn}-[$Zn_2L^{7g}{}_3$][ClO_4]$_4$ (Δ_{Zn}-**11g**)

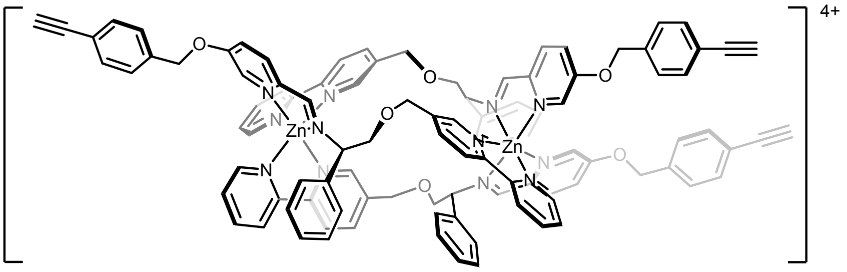

Δ_{Zn}-[Zn_2**L**$^{7g}{}_3$]$^{4+}$

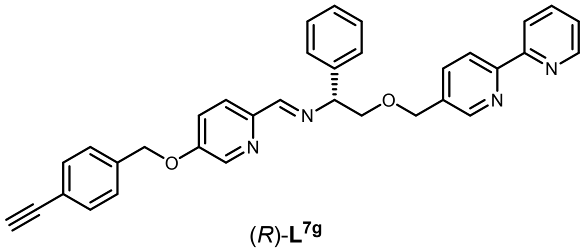

(*R*)-**L**^{7g}

0.04 g (0.13 mmol) of amine used.

Yield: 0.07 g, 0.03 mmol, 80%.

^{1}H NMR (500 MHz, 298 K, CD_3CN): δ_H 9.24-9.18 (m, 2H, N=CH, NC*H*CCH_2), 9.14-9.09 (m, 2H, N=CH, NC*H*CCH_2), 8.71 (s, 1H, N=CH), 8.50 (d, $^3J_{HH}$ = 4.5 Hz, 1H, Ar), 8.44 (t, $^3J_{HH}$ = 5.0 Hz, 1H, Ar), 8.38 (s, 2H, Ar), 8.34 (s, 1H, NC*H*CCH_2), 8.32-8.10 (m, 4H, Ar), 8.10-7.71 (m, 11H, Ar), 7.64-7.56 (m, 1H, Ar), 7.56-7.35 (m, 5H, Ar), 7.34-7.17 (m, 5H, Ar), 7.13-7.05 (m, 2H, Ar), 7.01 (m, 1H, Ar), 6.98-6.77 (m, 7H, Ar), 6.70 (t, $^3J_{HH}$ = 6.0 Hz, 2H, OCH_2CHCCHC*H*), 6.55 (t, $^3J_{HH}$ = 6.0 Hz, 2H, OCH_2CHCCHC*H*), 6.09 (d, $^3J_{HH}$ = 9.0 Hz, 2H, OCH_2CHCC*H*), 5.94 (d, $^3J_{HH}$ = 8.0 Hz, 2H, OCH_2CHCC*H*), 5.41 (dd, $^3J_{HH}$ = 11.5, 3.5 Hz, 1H, CH_2C*H*), 5.21-5.14 (m, 4H, PyOCH_2, bpy-CH_2), 5.12 (s, 2H, PyOCH_2), 5.02 (s, 2H, PyOCH_2), 4.92 (dd, $^3J_{HH}$ = 11.5, 3.0 Hz, 1H, CH_2C*H*), 4.78 (dd, $^3J_{HH}$ = 11.0, 4.5 Hz, 1H, CH_2C*H*), 4.52 (m, 4H, bpy-CH_2), 4.27 (t, $^3J_{HH}$ = 11.0 Hz, 1H, CH_2CH), 4.13 (t, $^3J_{HH}$ = 11.0 Hz, 1H, CH_2CH), 4.08-4.00 (m, 1H, CH_2CH), 3.71-3.64 (m, 3H, C≡CH), 3.60 (dd, $^2J_{HH}$ = 10.5 Hz, $^3J_{HH}$ = 3.0 Hz, 1H, CH_2CH), 3.51 (dd, $^2J_{HH}$ = 11.0 Hz, $^3J_{HH}$ = 2.5 Hz, 1H, CH_2CH), 3.44 (dd, $^2J_{HH}$ = 11.0 Hz, $^3J_{HH}$ = 3.5 Hz, 1H, CH_2CH).

2. NMR Spectra

2.1 Fe metallohelices

^{1}H (500 MHz, 298K) and ^{13}C NMR (126 MHz, 298K) spectra of Δ_{Fe}-$[Fe_2L_3]Cl_4$ metallohelices were recorded in CD_3OD or D_2O. Peaks at 3.31 ppm and 4.87 ppm in CD_3OD ^{1}H spectra are attributed to CD_3OD and H_2O, respectively, with peaks at 49.0 ppm in the ^{13}C spectra attributed to CD_3OD. Peaks at 4.79 ppm in D_2O ^{1}H spectra are attributed to H_2O.

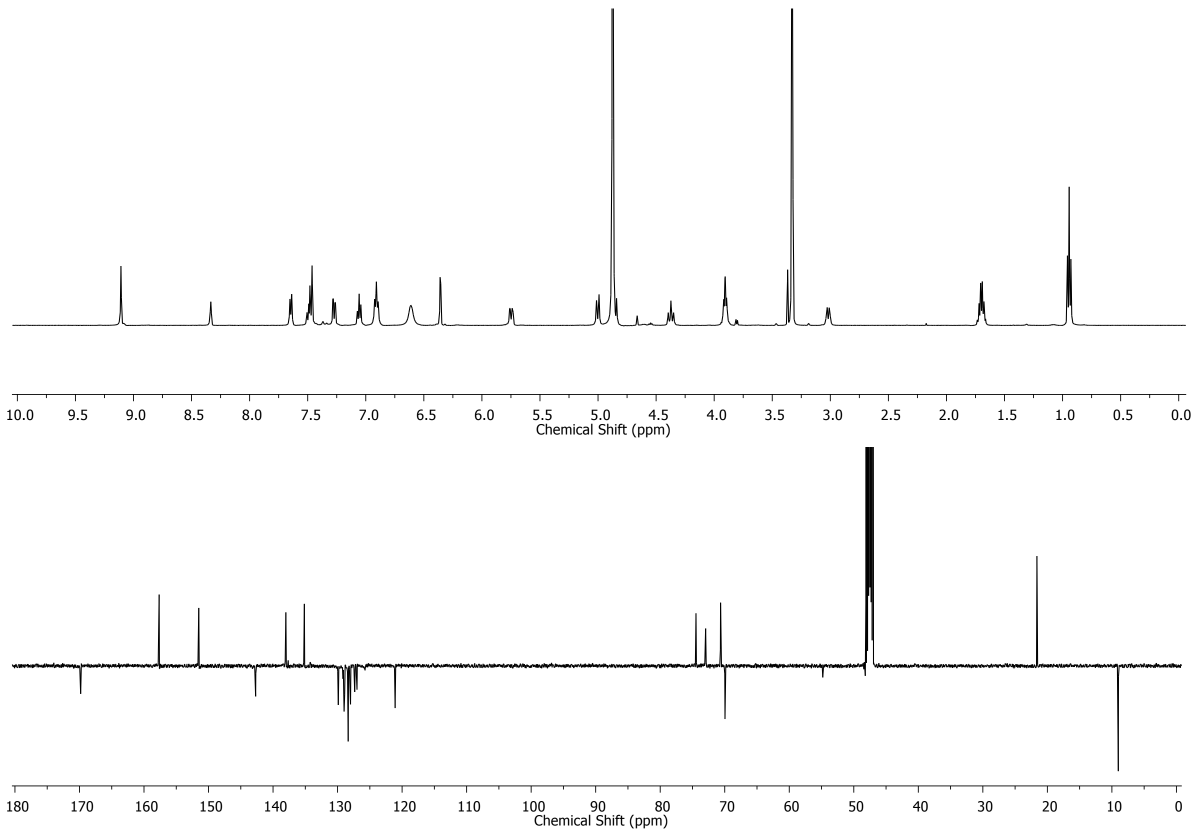

Figure S1 ^{1}H (500 MHz, CD_3OD, 298K) and ^{13}C (126 MHz, CD_3OD, 298K) NMR spectra of Δ_{Fe}-$[Fe_2L^{5c}_3]Cl_4\cdot 9H_2O$ (**Δ_{Fe}-9c**).

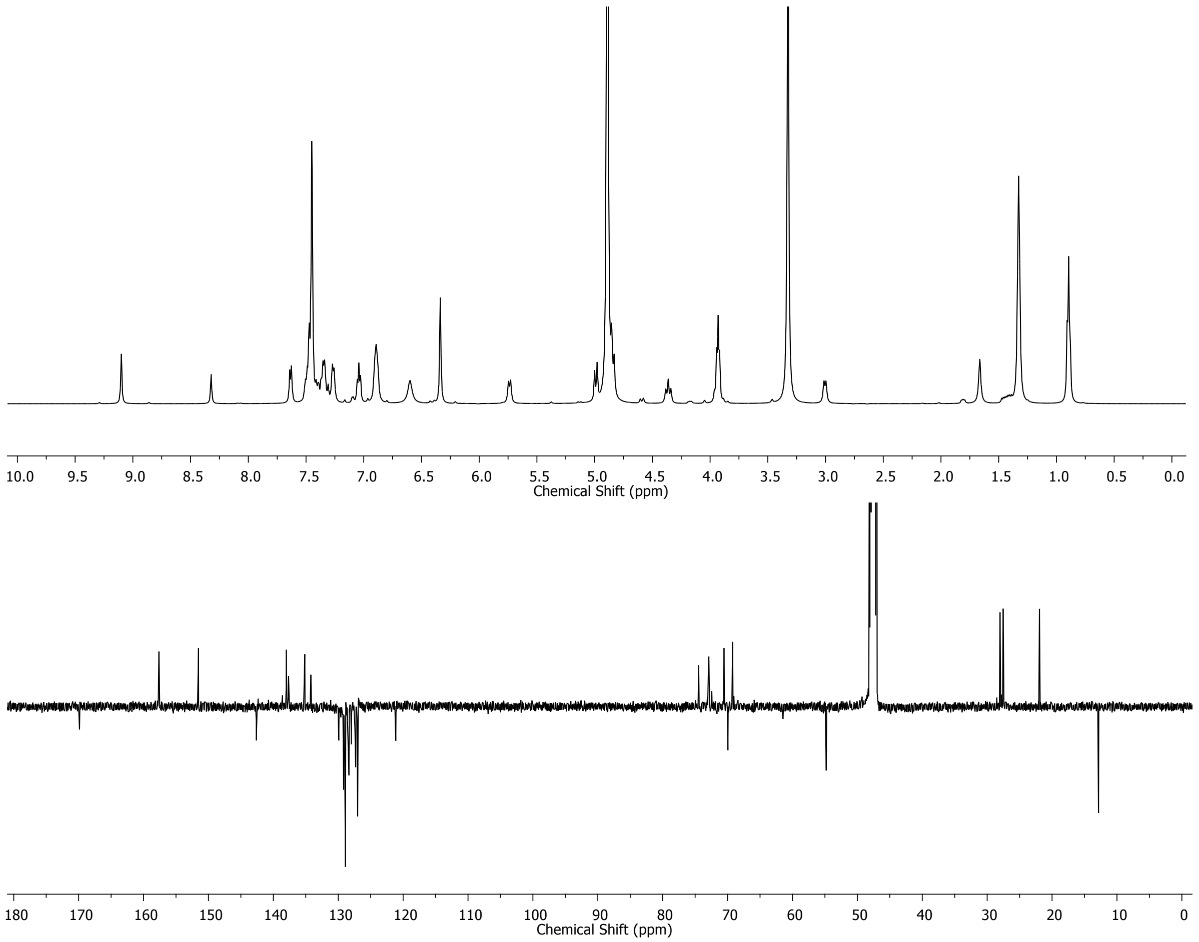

Figure S2 ^{1}H (500 MHz, CD_3OD, 298K) and ^{13}C (126 MHz, CD_3OD, 298K) NMR spectra of Δ_{Fe}-[$Fe_2L^{5d}_3$]$Cl_4\cdot 9H_2O$ (**Δ_{Fe}-9d**).

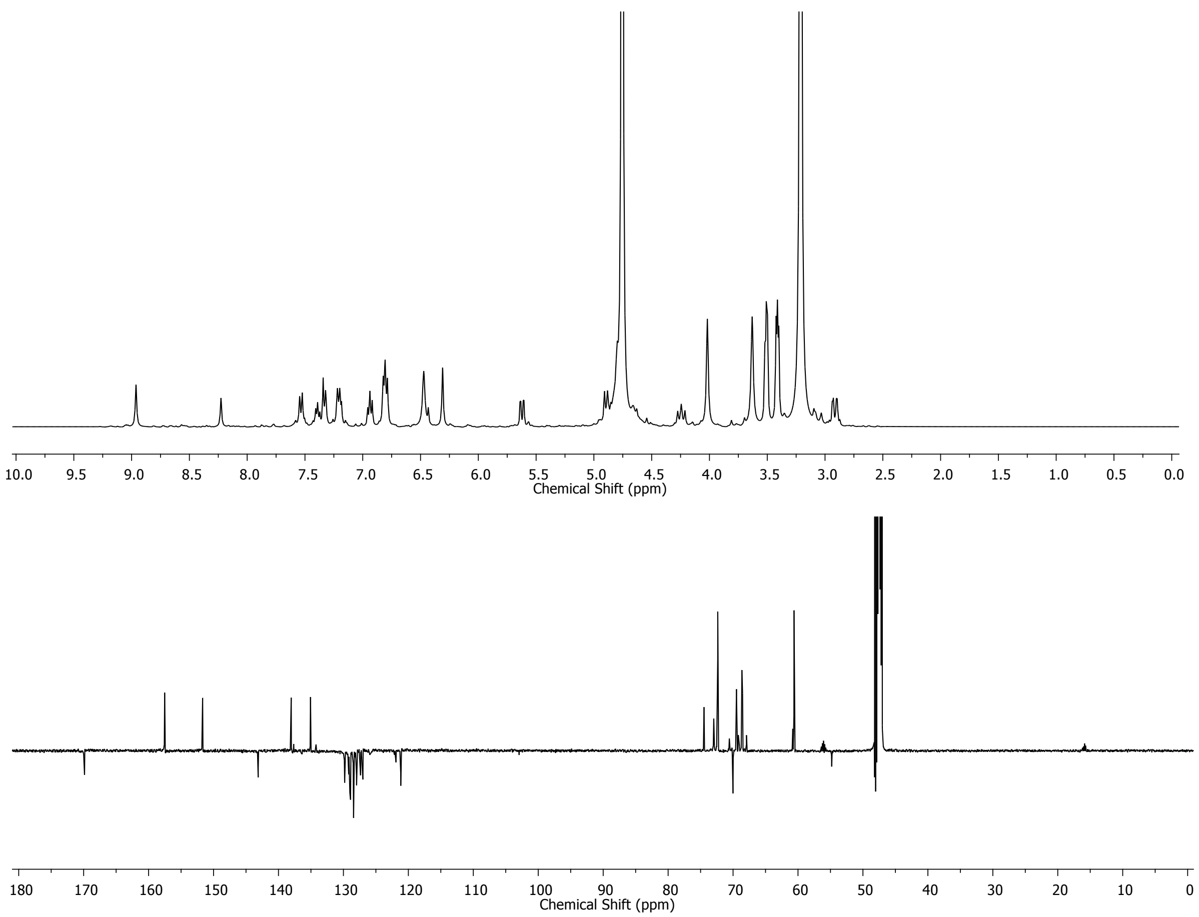

Figure S3 ^{1}H (500 MHz, CD_3OD, 298K) and ^{13}C (126 MHz, CD_3OD, 298K) NMR spectra of Δ_{Fe}-$[Fe_2L^{5e}{}_3]Cl_4\cdot 12H_2O$ (**Δ_{Fe}-9e**).

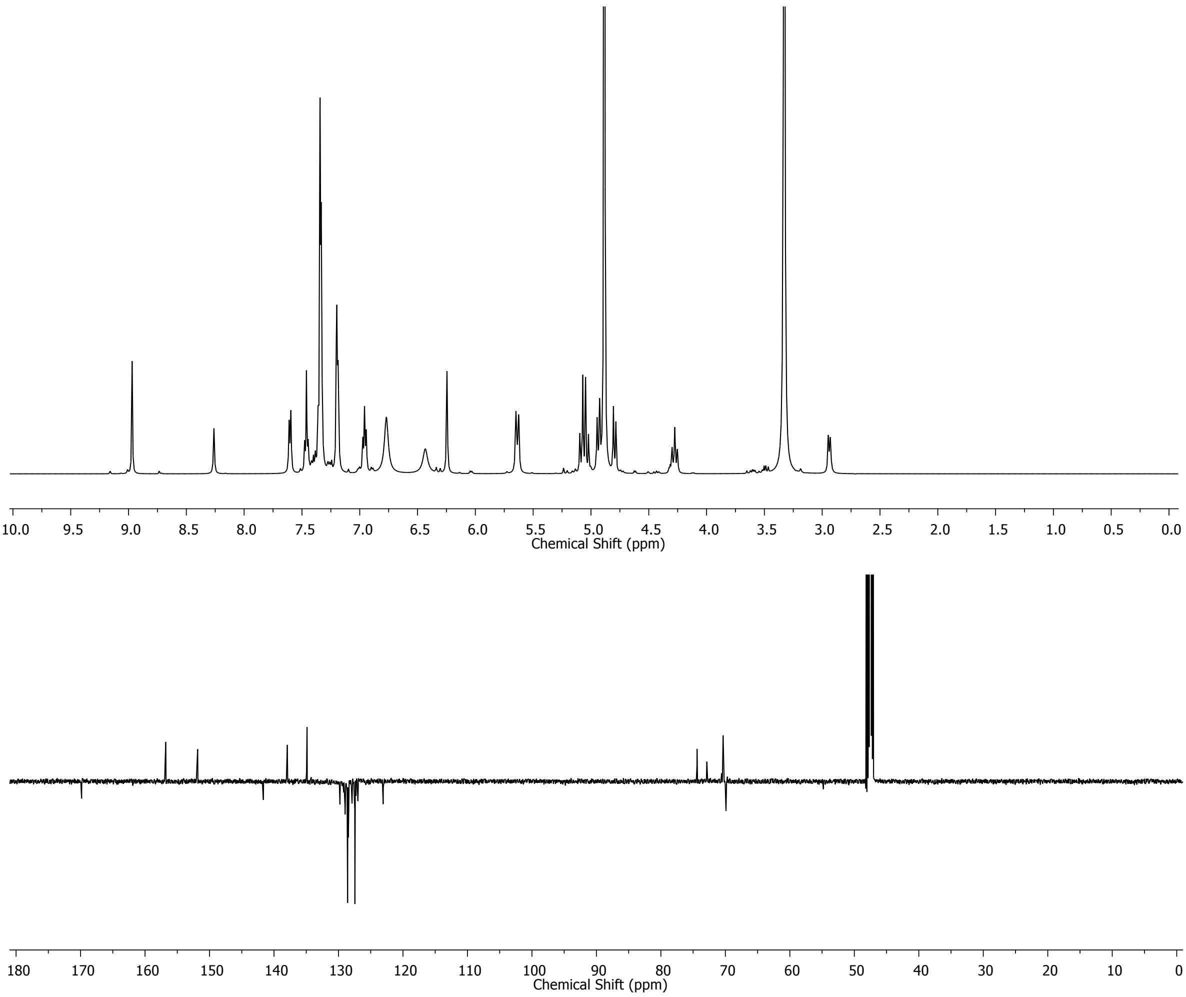

Figure S4 ^{1}H (500 MHz, CD_3OD, 298K) and ^{13}C (126 MHz, CD_3OD, 298K) NMR spectra of Δ_{Fe}-$[Fe_2L^{5f}_3]Cl_4{\cdot}8H_2O$ (**Δ_{Fe}-9f**).

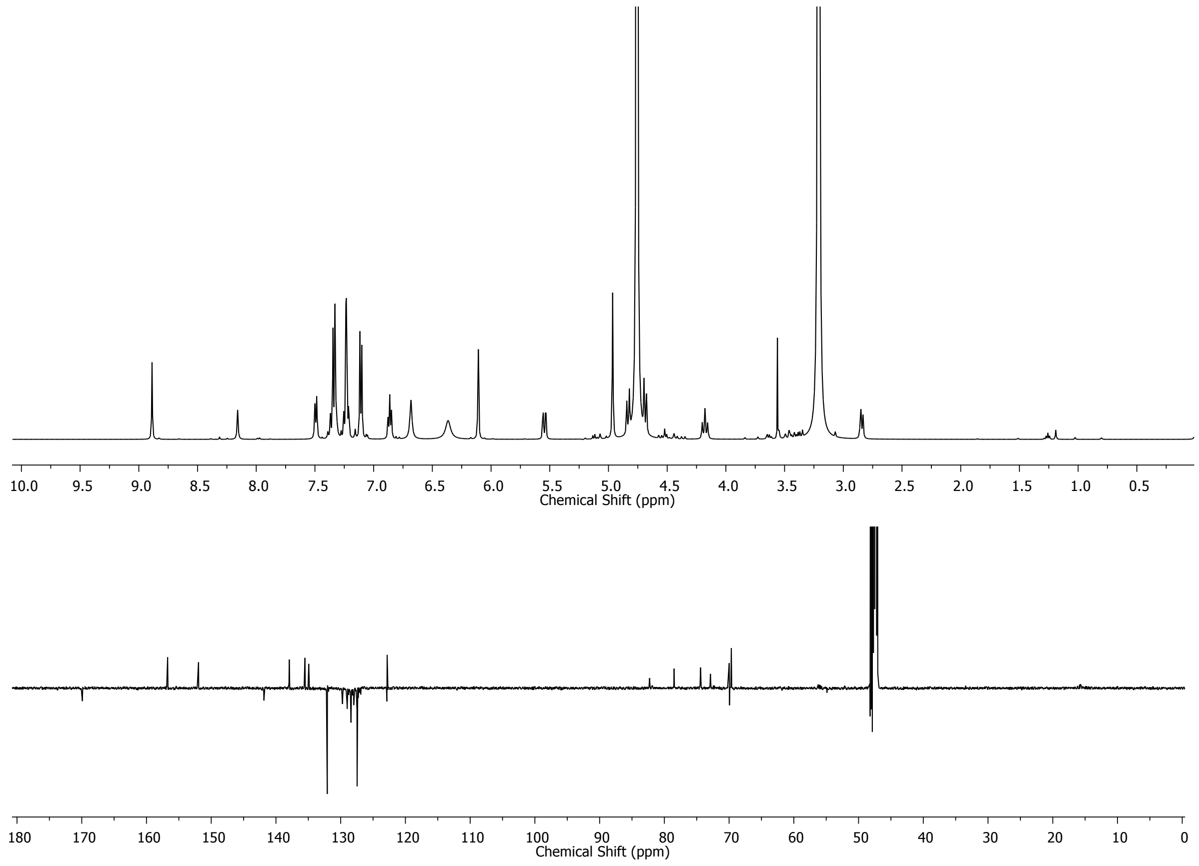

Figure S5 ^{1}H (500 MHz, CD_3OD, 298K) and ^{13}C (126 MHz, CD_3OD, 298K) NMR spectra of Δ_{Fe}-$[Fe_2L^{5g}{}_3]Cl_4\cdot 6H_2O$ (**Δ_{Fe}-9g**).

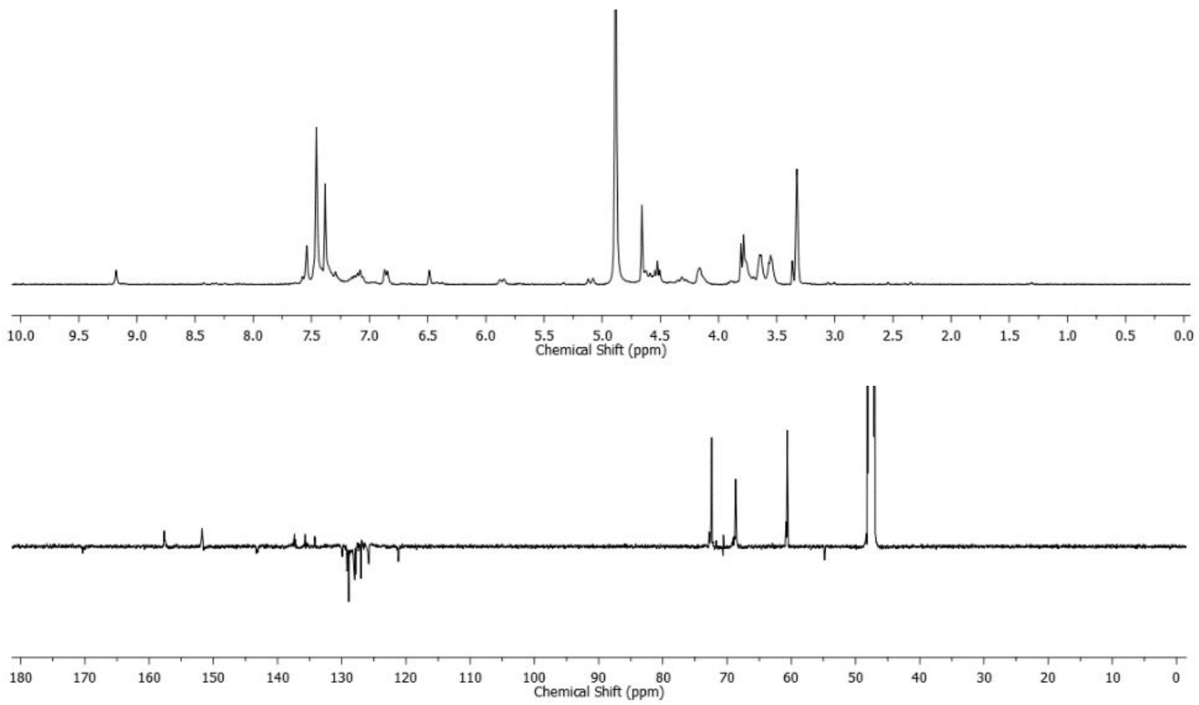

Figure S6 ^{1}H (500 MHz, CD_3OD, 298K) and ^{13}C (126 MHz, CD_3OD, 298K) NMR spectra of Δ_{Fe}-$[Fe_2L^{6e}{}_3]Cl_4\cdot 11H_2O$ (**Δ_{Fe}-10e**).

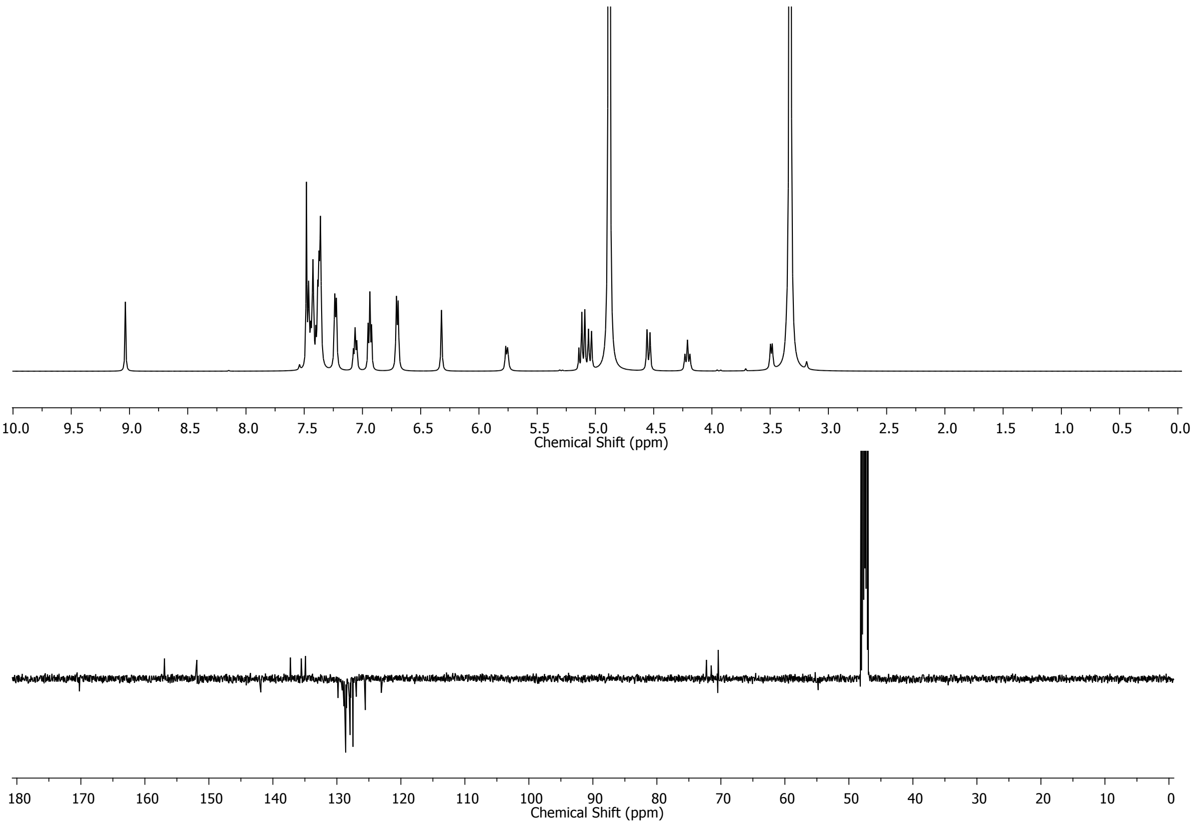

Figure S7 ^{1}H (500 MHz, CD_3OD, 298K) and ^{13}C (126 MHz, CD_3OD, 298K) NMR spectra of Δ_{Fe}-$[Fe_2L^{6f}{}_3]Cl_4\cdot 7H_2O$ (**Δ_{Fe}-10f**).

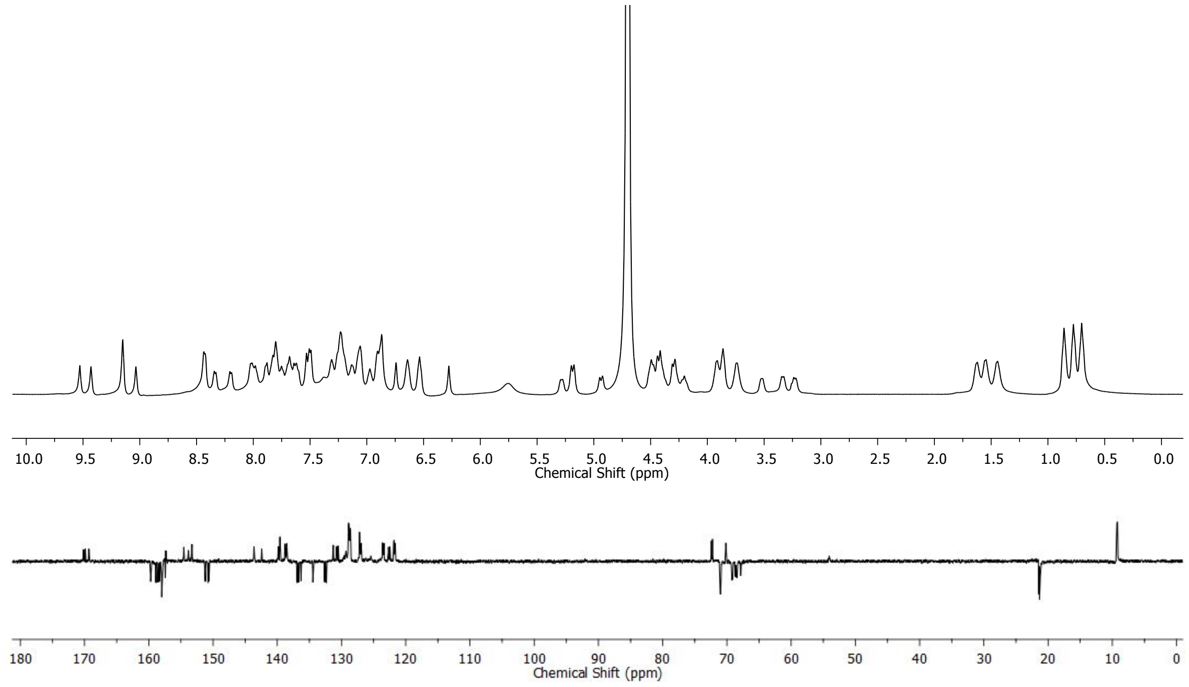

Figure S8 ^{1}H (500 MHz, D_2O, 298K) and ^{13}C (126 MHz, D_2O, 298K) NMR spectra of Δ_{Fe}-$[Fe_2L^{7c}{}_3]Cl_4\cdot 10H_2O$ (**Δ_{Fe}-11c**).

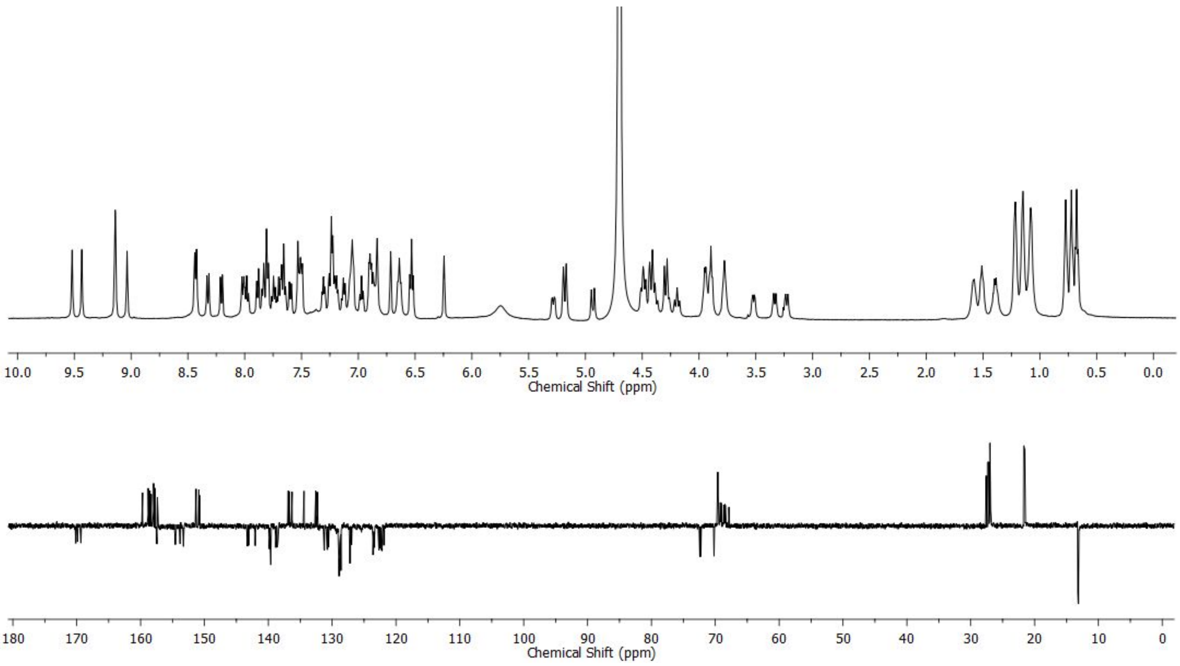

Figure S9 ^{1}H (500 MHz, D_2O, 298K) and ^{13}C (126 MHz, D_2O, 298K) NMR spectra of Δ_{Fe}-$[Fe_2L^{7d}{}_3]Cl_4 \cdot 5H_2O$ (**Δ_{Fe}-11d**).

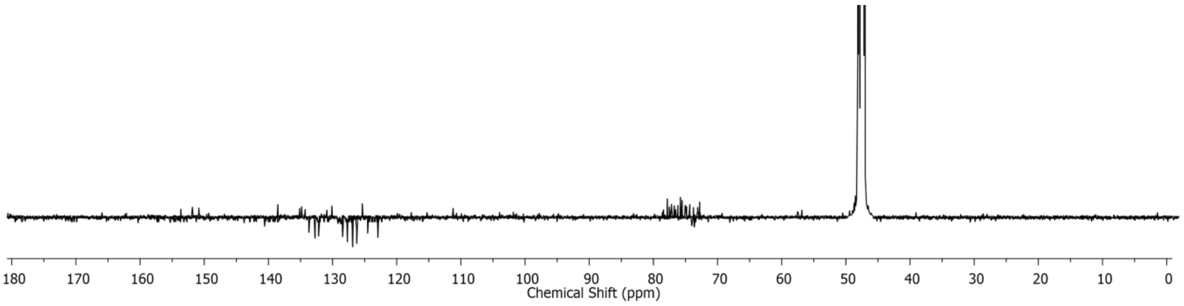

Figure S10 ^{13}C (126 MHz, CD_3OD, 298K) NMR spectrum of Δ_{Fe}-$[Fe_2L^{7e}{}_3]Cl_4 \cdot 8H_2O$ (**Δ_{Fe}-11e**).

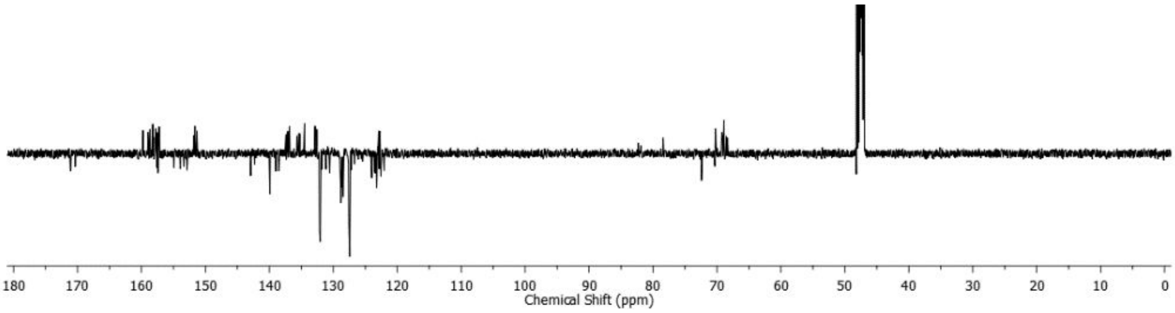

Figure S11 ^{13}C (126 MHz, CD_3OD, 298K) NMR spectrum of Δ_{Fe}-$[Fe_2L^{7g}{}_3]Cl_4 \cdot 10H_2O$ (**Δ_{Fe}-11g**).

2.2 Zn metallohelices

^{1}H (500 MHz, 298K) and ^{13}C NMR (126 MHz, 298K) spectra of Δ_{Zn}-$[Zn_2L_3][ClO_4]_4$ metallohelices were recorded in CD_3CN. Peaks at 1.94 ppm and 2.13 ppm in CD_3CN ^{1}H spectra are attributed to CD_3CN and H_2O, respectively, with peaks at 118.3 ppm in the ^{13}C spectra attributed to CD_3CN.

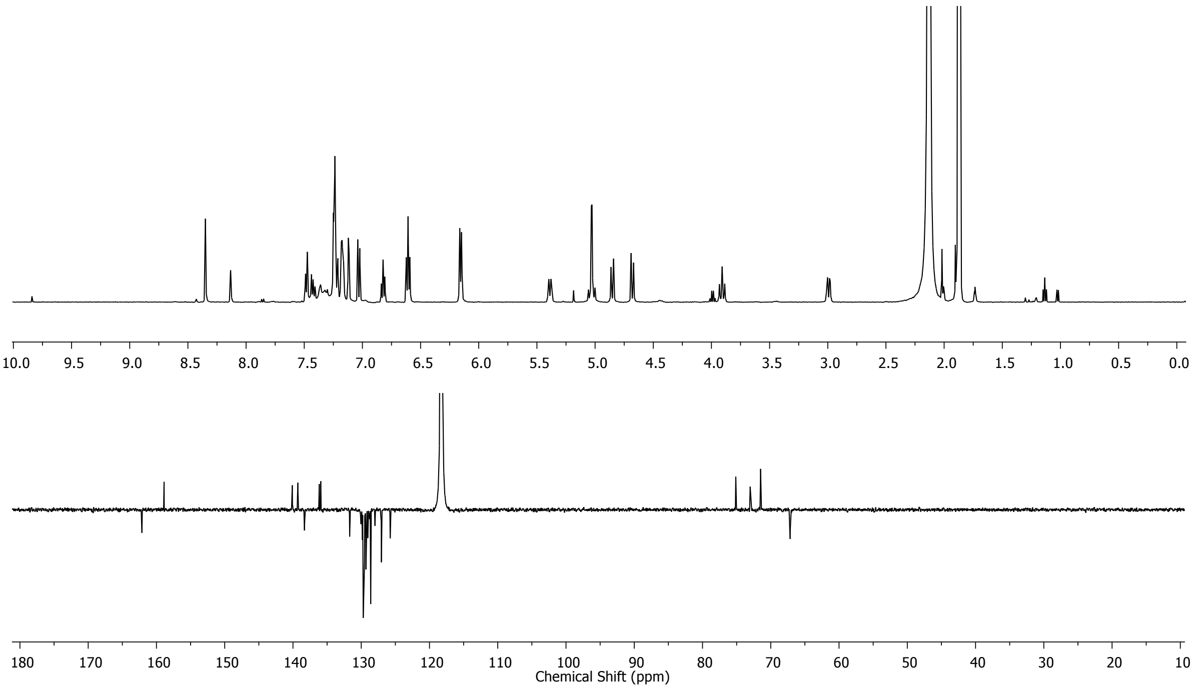

Figure S12 ^{1}H (500 MHz, CD_3CN, 298K) and ^{13}C (126 MHz, CD_3CN, 298K) NMR spectra of Δ_{Zn}-$[Zn_2L^{5f}{}_3][ClO_4]_4$ (**Δ_{Zn}-9f**).

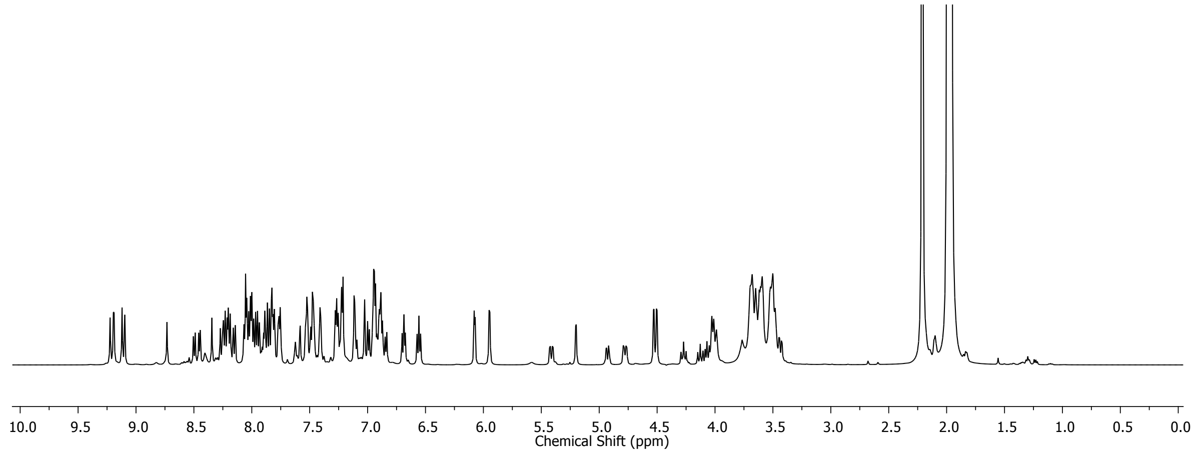

Figure S13 ^{1}H (500 MHz, CD_3CN, 298K) NMR spectrum of Δ_{Zn}-$[Zn_2L^{7e}{}_3][ClO_4]_4$ (**Δ_{Zn}-11e**).

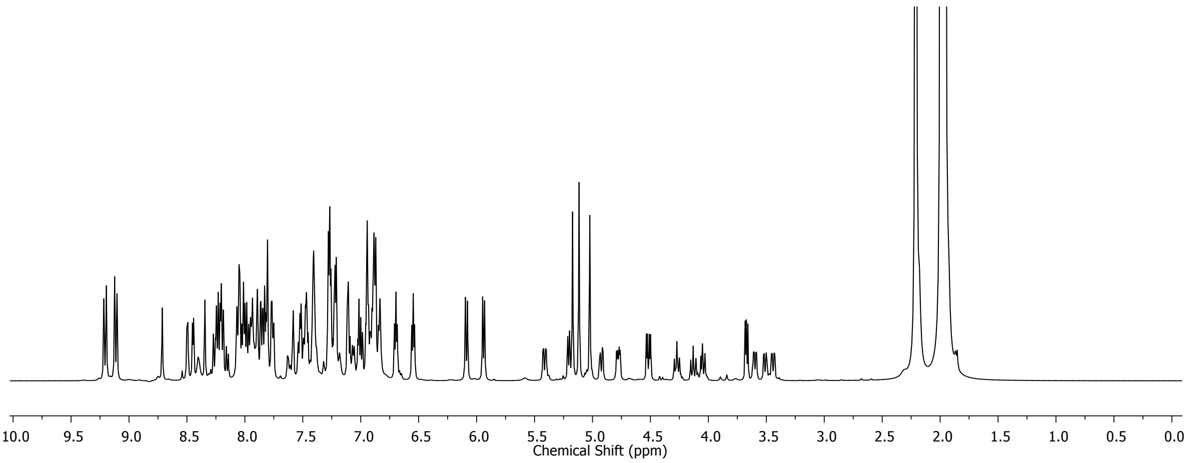

Figure S14 ^{1}H (500 MHz, CD_3CN, 298K) NMR spectrum of Δ_{Zn}-$[Zn_2L^{7g}{}_3][ClO_4]_4$ (**Δ_{Zn}-11g**).

3. High resolution ESI mass spectra

High-resolution ESI mass spectra focus on the $[Fe_2L_3]^{4+}$ tetracation isotopic distributions of the $[Fe_2L_3]Cl_4$ enantiomer pairs. Displayed is the measured spectrum (upper) and a simulated mass spectrum (lower) over the same m/z range for comparison.

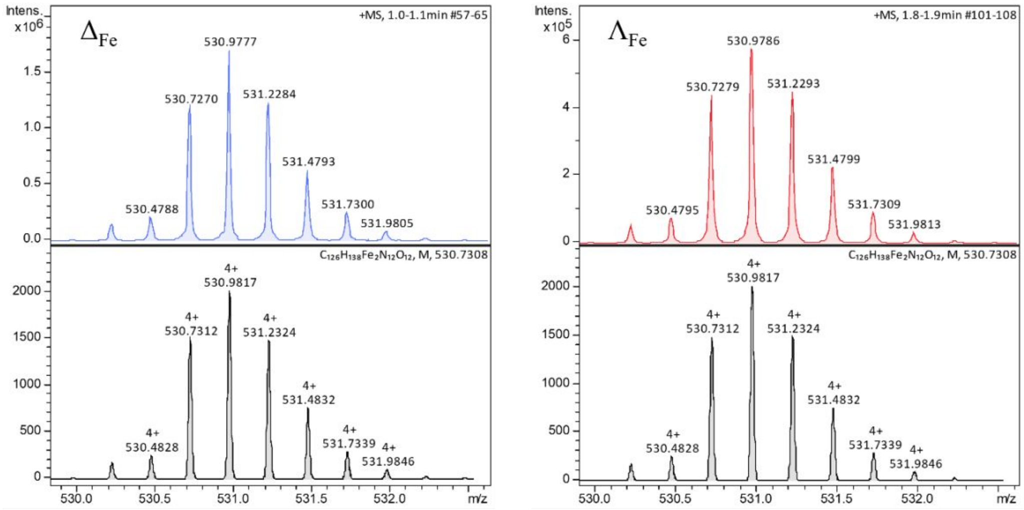

Figure S15 High resolution ESI mass spectra of Δ- and Λ-$[Fe_2L^{5c}_3]Cl_4·9H_2O$ (**9c**).

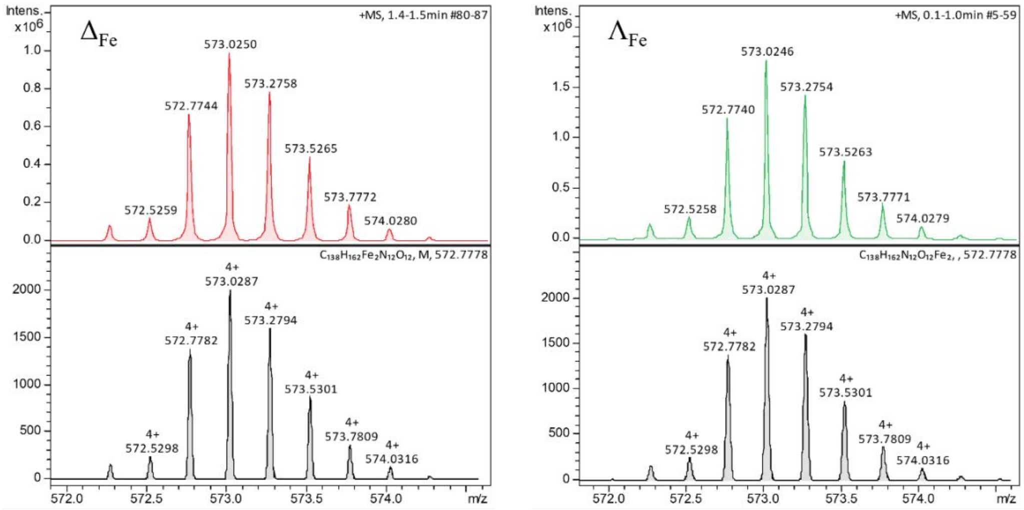

Figure S16 High resolution ESI mass spectra of Δ- and Λ-$[Fe_2L^{5d}_3]Cl_4·9H_2O$ (**9d**).

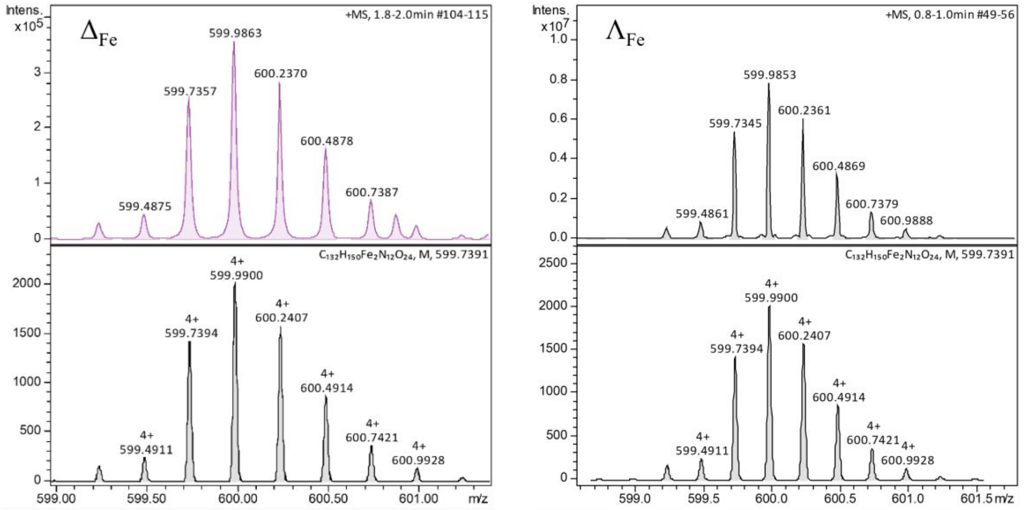

Figure S17 High resolution ESI mass spectra of Δ- and Λ-$[Fe_2L^{5e}{}_3]Cl_4{\cdot}12H_2O$ (**9e**).

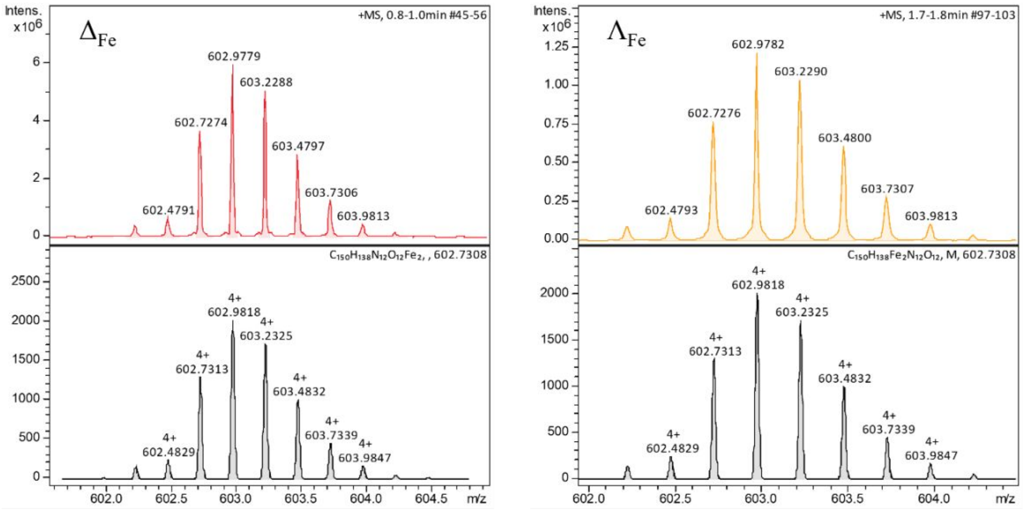

Figure S18 High resolution ESI mass spectra of Δ- and Λ-$[Fe_2L^{5f}{}_3]Cl_4{\cdot}8H_2O$ (**9f**).

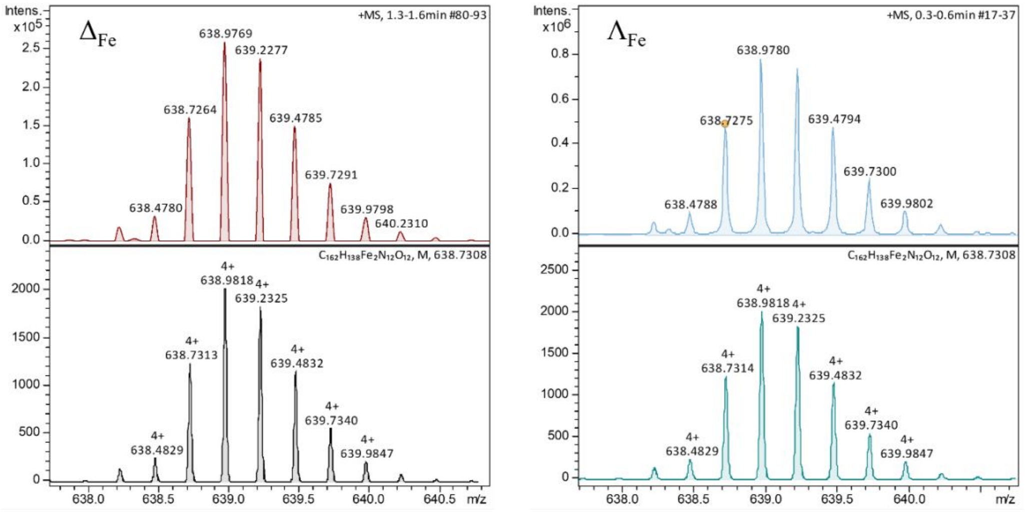

Figure S19 High resolution ESI mass spectra of Δ- and Λ-$[Fe_2L^{5g}{}_3]Cl_4{\cdot}6H_2O$ (**9g**).

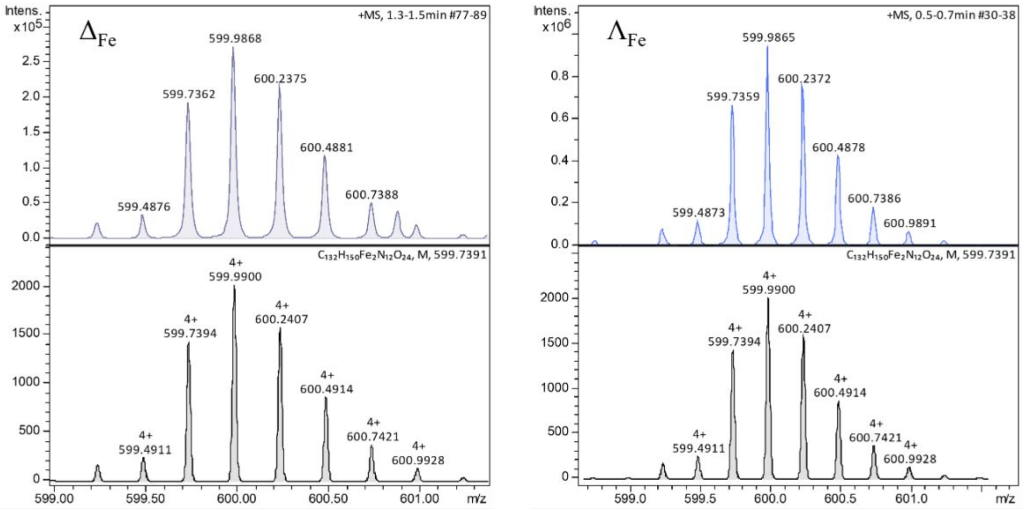

Figure S20 High resolution ESI mass spectra of Δ- and Λ-$[Fe_2L^{6e}{}_3]Cl_4\cdot 11H_2O$ (**10e**).

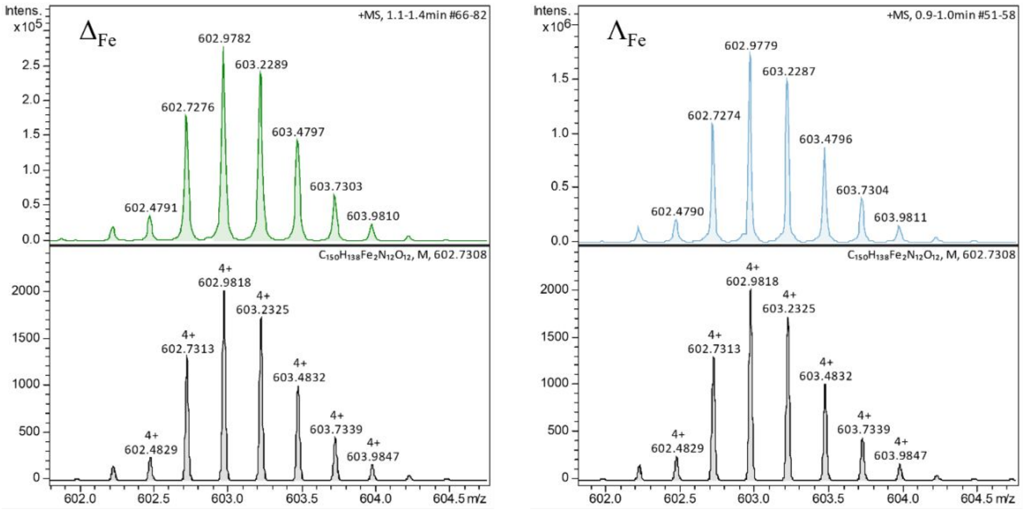

Figure S21 High resolution ESI mass spectra of Δ- and Λ-$[Fe_2L^{6f}{}_3]Cl_4\cdot 7H_2O$ (**10f**).

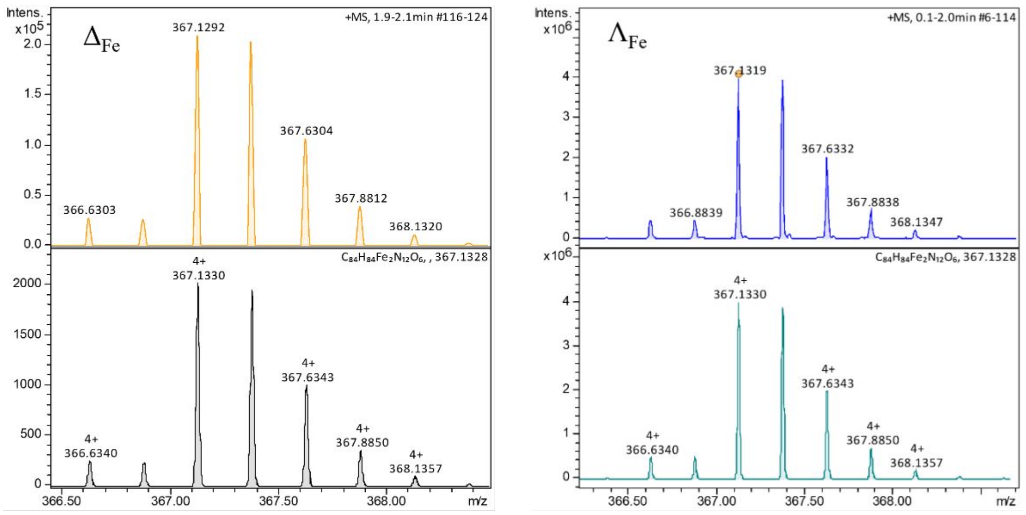

Figure S22 High resolution ESI mass spectra of Δ- and Λ-$[Fe_2L^{7c}{}_3]Cl_4\cdot 7H_2O$ (**11c**).

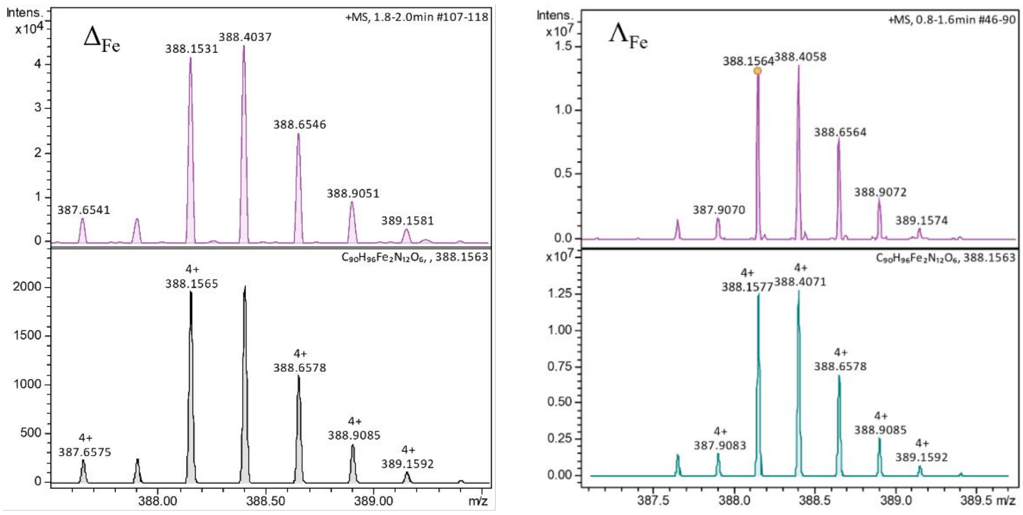

Figure S23 High resolution ESI mass spectra of Δ- and Λ-$[Fe_2L^{7d}{}_3]Cl_4\cdot 5H_2O$ (**11d**).

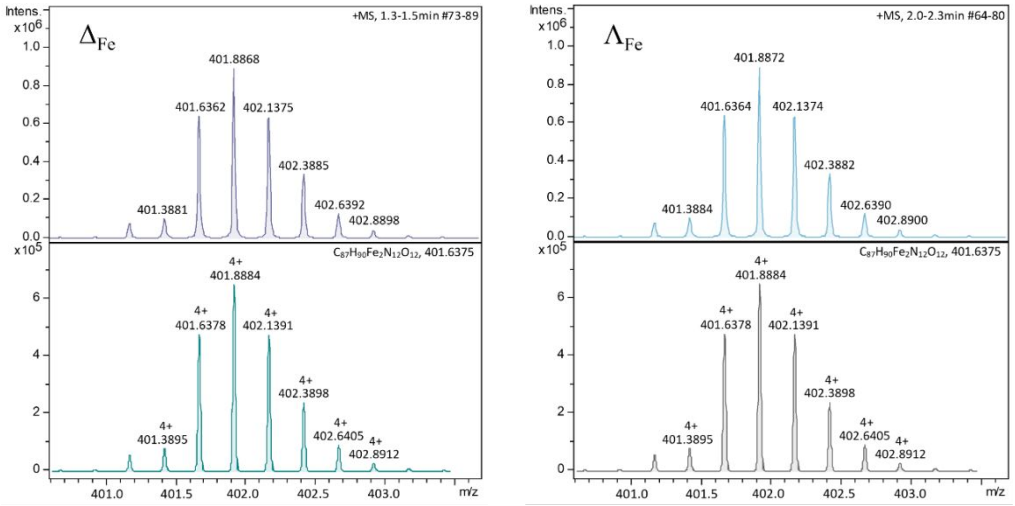

Figure S24 High resolution ESI mass spectra of Δ- and Λ-$[Fe_2L^{7e}{}_3]Cl_4\cdot 8H_2O$ (**11e**).

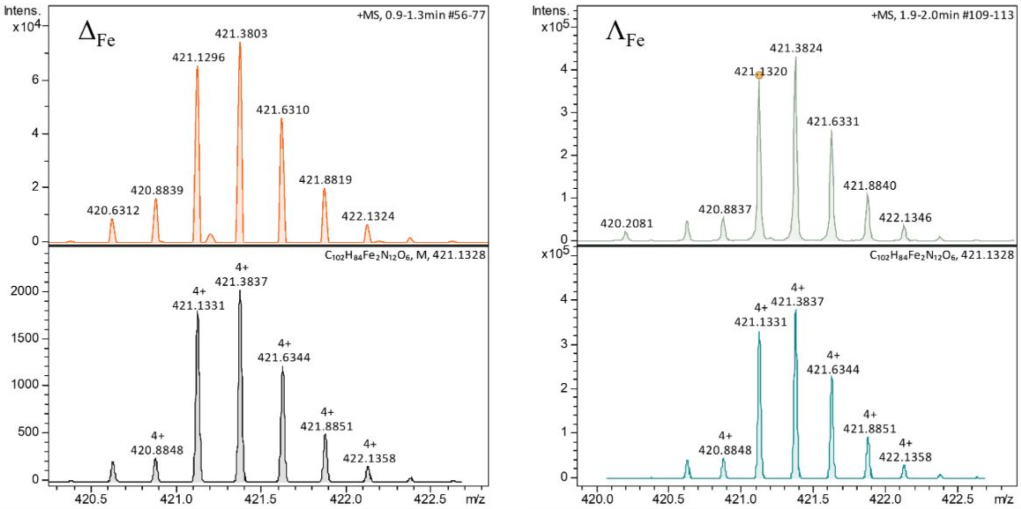

Figure S25 High resolution ESI mass spectra of Δ- and Λ-$[Fe_2L^{7g}{}_3]Cl_4\cdot 10H_2O$ (**11g**).

4. Stability Studies

4.1 Aqueous and biological media stability

Flexicate and triplex compounds were prepared to a concentration of 0.03 mM in PBS, water/HCl acid buffer (pH 1.5) or CAMHB. Solutions were sealed in 1 cm path-length plastic macrocuvettes and wrapped with parafilm to reduce evaporation. Initial UV-Vis photoabsorption spectra were recorded immediately after solution preparation, following which the cuvettes were incubated at 310 K and absorbances at appropriate wavelengths and time intervals were recorded. Intermittent spectra were taken after 1, 4, 7, 14 and 28 days of incubation for PBS and CAMHB compounds, and absorbances at a defined wavelengths were recorded every 5 min for 12 h for compounds in acid buffer. UV-Vis spectra of PBS, water/HCl buffer and CAMHB were also recorded in conjunction with the compound solutions and used as baseline measurements when appropriate.

4.2 Deferoxamine (DFO) stability

Aqueous DFO was added to aqueous solutions of 10 µM Λ-**9a** and Λ-**11a** to a DFO concentration of 20 µM and 100 µM, and the UV-Vis spectra were recorded for 5 d. Equimolar solutions (1mM) of DFO:$FeCl_2$ and DFO:$FeCl_3$ were prepared as controls.

<10% Degradation was observed for Λ-**9a** and Λ-**11a** across the 5 d monitoring period in all cases (approximately in line with compound degradation in PBS), with no sign of the characteristic 430 nm peak of Fe-DFO complex as observed in the $FeCl_2$ and $FeCl_3$ controls.

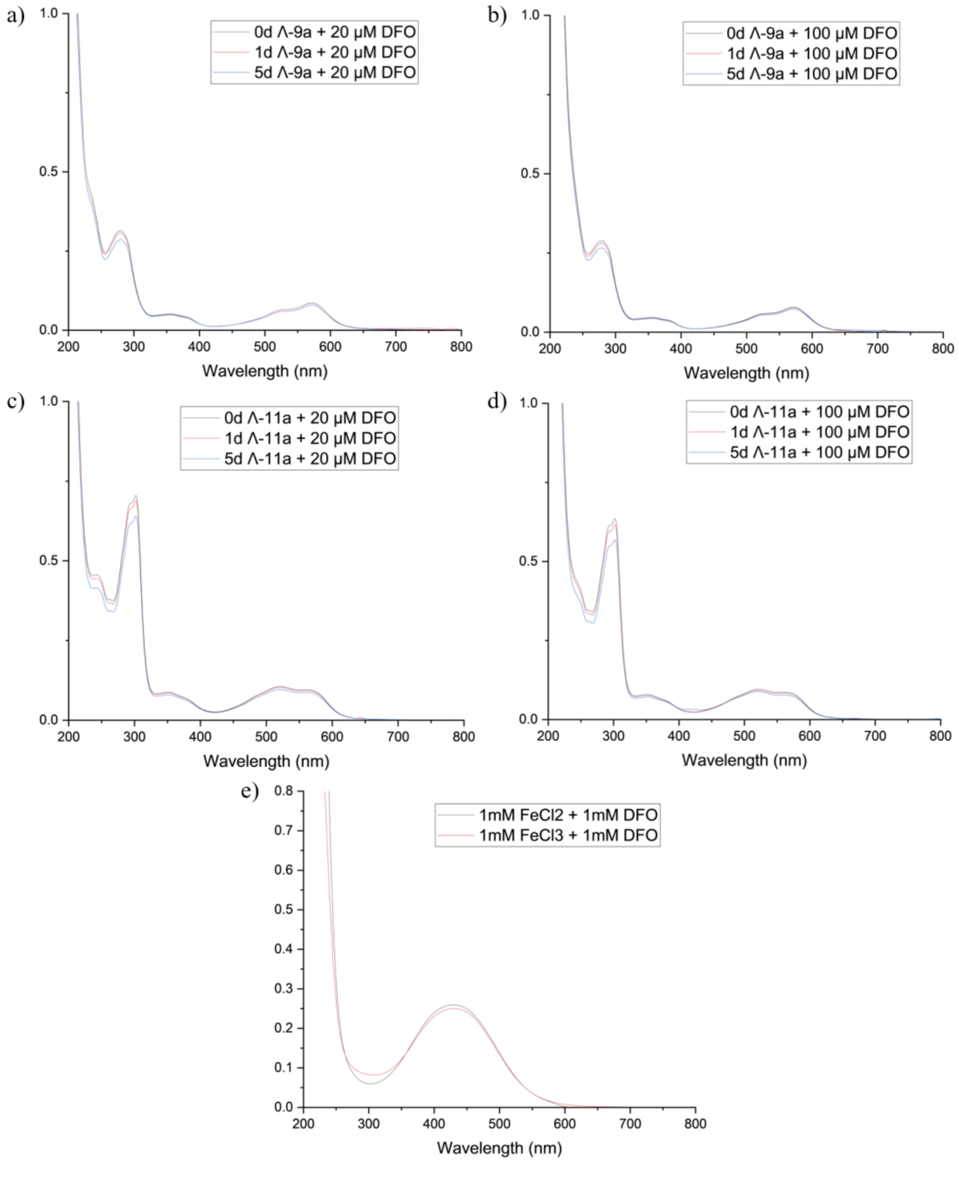

Figure S26 UV-Vis spectra recorded at 0, 1, and 5 d for 10 µM (a+b) Λ-**9a** and (c+d) Λ-**11a** in the presence of (a+c) 20 µM and (b+d) 100 µM DFO. As controls, spectra of equimolar DFO:$FeCl_2$ and DFO:$FeCl_3$ (e) were also measured.

5. Model vesicle studies

5.1 Preparation of model vesicles

Lipid films were formulated by dissolving the chosen lipids (20 mg total, see below) in chloroform:methanol (2:1, v:v, 1.5 ml), and removing the solvent using a rotary evaporator (bath at 20 °C, 1 h) to deposit a thin film on the wall of a round-bottomed flask. The films were hydrated with sodium phosphate buffer (6.7 ml, 25 mM, pH 7.4), to give stock solutions (3 mg ml^{-1} lipid). To form unilamellar vesicles, the aqueous lipid suspensions were subjected to four freeze/thaw/sonication cycles: sonication of the sample (4 × 30 s); freezing the sample at -20 °C; thawing the sample at room temperature. The following phospholipids which were used for each membrane-mimetic system: *E. coli* – POPE: 13.40 mg (67.0 wt. %), POPG: 4.64 mg (23.2 wt. %), CL: 1.96 mg (9.8 wt. %); *S. aureus* – POPG: 11.60 mg (58.0 wt. %), CL: 8.40 mg (42.0 wt. %).

5.2 Zeta Potential Measurement Graphs

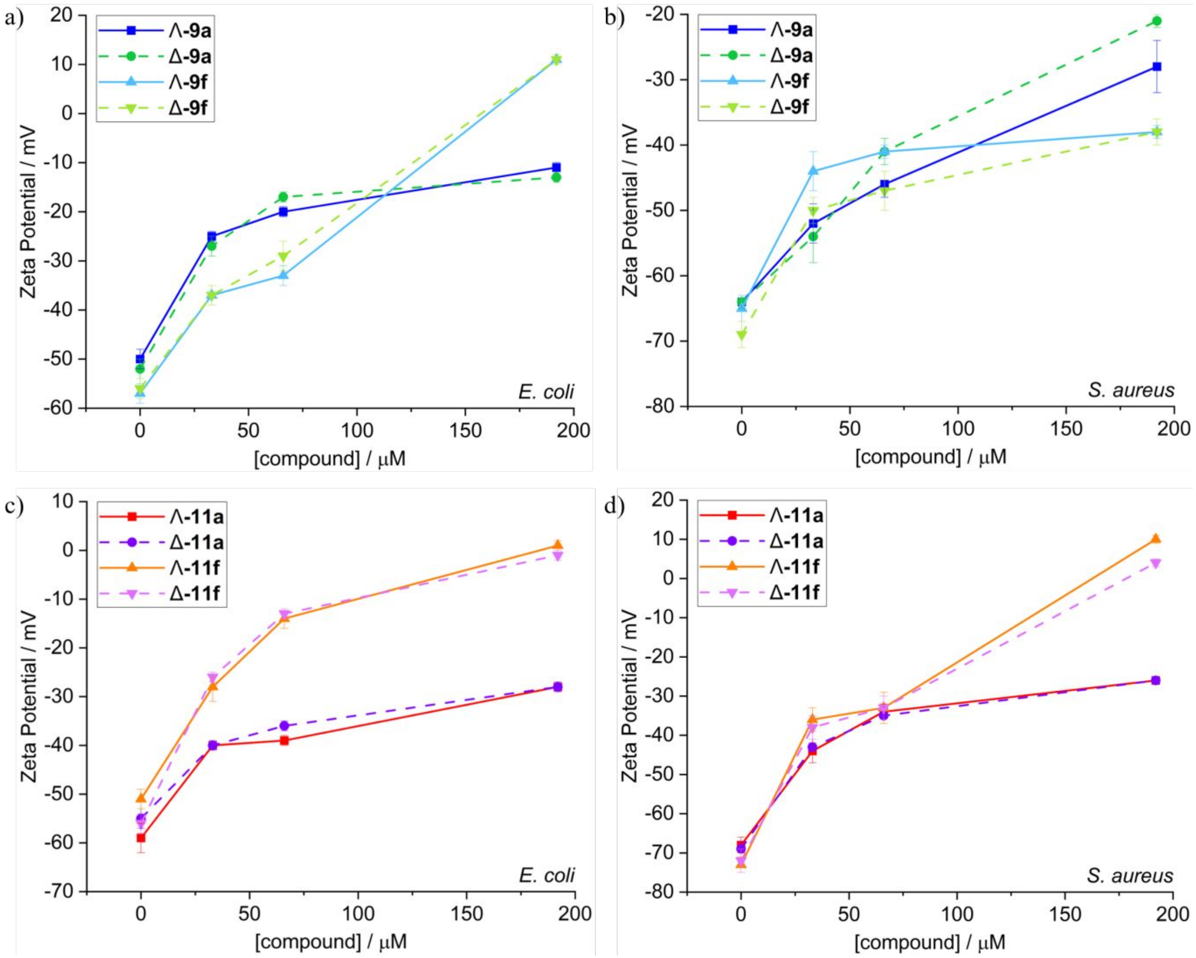

Figure S27 Zeta potential measurements for addition of (a+b) flexicates and (c+d) triplexes to membrane-mimetic unilamellar vesicles models based on (a+c) *E. coli* and (b+d) *S. aureus* outer leaflet of inner membranes. Lipids at 0.5 mg ml^{-1} (~0.6 mM) in sodium phosphate buffer (25 mM, pH 7.4) at 25 °C. Mean of five independent measurements ± 1 standard deviation.

6. Microbiology methods

6.1 General considerations

All procedures were performed using sterile techniques. Preceding experimental work, surfaces were washed thoroughly with 80% ethanol. Where necessary, equipment was either purchased sterile or thoroughly sterilised, and reagents autoclaved prior to use. Cation-adjusted Müller-Hinton broth (CAMHB) and Roswell Park Memorial Institute (RPMI) 1640 media were purchased from Sigma-Aldrich, with CAMHB catalogued as Mueller Hinton Broth 2. Sterile growth media, agar plates, buffers, PBS, and double-distilled water were prepared by the media preparation service of the University of Warwick's School of Life Sciences, unless stated otherwise. Unless stated, incubation periods were 24 h and without agitation using a Memmert INB200 incubator oven or an Eppendorf New Brunswick S41i CO_2 incubator shaker. Optical densities at 600 nm (OD_{600}) of bacterial cultures in broth were measured using Jenway 6300 benchtop spectrophotometers. Centrifugations were performed using an Heraeus Sepatech Biofuge 13 3637 benchtop centrifuge. For all assays, appropriate positive and negative controls were incorporated, with at least two replicates performed for each measurement. Stock solutions of complexes were prepared in pure water where possible, with less water-soluble compounds **9c,d,g** and **10c,d,g** prepared by dissolution in minimal DMSO prior to water addition, such that before dilution it contained 10% v/v DMSO, with the final DMSO concentration in the assay calculated to be approximately 0.8% v/v.

6.2 Bacterial minimum inhibitory concentration (MIC) determination

A standard broth microdilution method was employed, in agreement with the CLSI guidelines.[12,13]

A 3.2 mg ml^{-1} stock solution of each compound was prepared in water or DMSO/water mix (1:10), corresponding to a 12.5-fold concentration of the highest concentration tested, 256 µg ml^{-1}. In a sterile 96-well plate, 32 µl of each 3.2 mg ml^{-1} stock was added to 168 µl of antibiotic-free CAMHB to return 512 µg ml^{-1} solutions. These solutions were subject to twofold serial dilution in CAMHB. Overnight cultures of each bacterial strain in CAMHB were diluted in the same medium to a cell concentration of 1×10^6 CFU ml^{-1}, before 100 µl addition of this culture to each compound well, allowing a 0.008-256 µg ml^{-1} compound concentration range to be tested. Plates were incubated at 37 °C for 20 h without shaking, and the lowest concentration deemed to inhibit >99% of bacterial growth for each compound was judged to be the MIC. Positive (culture only, no antimicrobial) and negative controls (CAMHB only) were used to ensure suitable bacterial growth and no contamination of media, respectively. Appropriate clinical antimicrobials (ampicillin, tetracycline, ticarcillin, etc.) were used as quality controls and compared to literature values to validate MIC values.[12,14-16] Results were repeated in triplicate.

6.3 Bacterial minimum bactericidal concentration (MBC) determination

Determination of MBCs was carried out for strain/compound pairings where an MIC ≤128 µg ml^{-1} was determined, immediately following the MIC assay. For each 'culture' with compound concentration in the range 128 µg ml^{-1} to the MIC (inclusive), 10 µl of the bacteria/compound mix was recovered from the microtitre plate for analysis. These were added to a sterile antimicrobial-free LB/agar plate. Upon overnight incubation (37 °C), plates were inspected, and the MBC was determined to be the lowest concentration of compound at which this dilution/culturing assay showed no visible signs of bacterial growth.

6.4 Haemolysis assays

Fresh equine blood was centrifuged (1000×g, 10 min) and the supernatant was removed. Harvested erythrocytes were washed three times with PBS and then resuspended to a 5% erythrocyte concentration in PBS. $[Fe_2L_3]Cl_4$ compounds were dissolved in PBS to form 3.2 mg ml^{-1} stock solutions. These stock solutions were used to prepare 1-1024 µg ml^{-1} serial dilution ranges in 96-well round bottom plates using PBS. The suspended erythrocytes (100 µl) were added to the $[Fe_2L_3]Cl_4$ solution wells (100 µl) and incubated without agitation (310 K,

1 h). The haemolytic concentration for each compound was determined for each compound by visual lysis inspection of wells was performed after the incubation period, in which the lowest concentration deemed to cause >10% cell lysis for each compound was judged to be the haemolytic concentration. Controls included a PBS and 1% Triton X-100 as 0 and 100% haemolysis, respectively. Each measurement was performed in triplicate.

6.5 Chemosensitivity (MTT assay)

ARPE-19 (human retinal pigment epithelial) cells were incubated in 96-well plates at a cell concentration of 0.5×10^4 cells ml^{-1}. The cells were used when between 50 and 80% confluent in the stock flasks. Complete cell media containing DMEM, supplemented with 10% foetal calf serum and L-glutamine (2 mM), was used to prepare the desired cell concentration and reference wells. Plates containing cells were incubated for 24 h at 37 °C in 5% CO_2 atmosphere, prior to drug exposure. Cell media (200 µl) was added to the reference cells and differing concentrations of drug solution (200 µl) were added to the remaining wells. The plates were incubated for a further 96 h at 37 °C in 5% CO_2 atmosphere. 3-(4,5-Dimethylthiazol-1-yl)-2,5-diphenyltetrazolium bromide (MTT) solution (0.5 mg ml^{-1}, 20 µl per well) was added to each well and incubated for 4 h at 37 °C in 5% CO_2 atmosphere. Upon completion all solutions were removed from the wells and DMSO (150 µl) was added to each well to dissolve the purple formazan crystals. A ThermoScientific Multiskan EX microplate photometer was used to measure the absorbance at 540 nm. Lanes containing 100% cell media and untreated cells were used as a blank and 100% cell survival, respectively. Cell survival was determined as the absorbance of treated cells minus the blank cell media, divided by the absorbance of the untreated control; this value was expressed as a percentage. The IC_{50} values were determined from a plot of percentage cell survival against drug concentration (µM). Assays were conducted in triplicate and the mean IC_{50} ± standard deviation was determined.

6.6 Antimicrobial stability of compounds at different storage durations and temperatures

512 µg ml^{-1} solutions of each compound in CAMHB were prepared in 1.5 ml Eppendorf tubes. These tubes were stored at three different temperatures (255 K, 293 K, 310 K) and the solutions were tested on days 1, 4, 7, 14 and 28 in MIC assays (as described in Section 6.2.3). To ensure only one freeze thaw cycle/no variance in temperature occurred for the 255 K and 310 K samples, the original 512 µg ml^{-1} samples were separated into 5 Eppendorf tubes, one for each testing day.

6.7 Synergy assays

A protocol for a standard checkerboard bacterial synergy assay was followed.[17] Compounds were prepared in water at 4× the highest desired concentration to test. 50 µl of CAMHB was added into each well of a sterile 96-well plate. 100 µl of the first compound of interest was added and serially diluted along the plate ordinate, followed by addition of 50 µl of the second compound and serial dilution along the abscissa. Overnight CAMHB cultures of *E. coli* (strain ATCC 25922) for metallohelix/metallohelix assays or *S. aureus* (strain USA300) for metallohelix/cefoxitin assays were diluted in the same medium to a cell concentration of 1×10^6 CFU ml^{-1}, before 100 µl addition of this culture to each compound well. The final checkerboard thus contains combinations of the two antibiotics, with the highest concentration of each antibiotic at opposite well corners, and an MIC row and column for each of the two compounds individually. Plates were incubated at 37 °C for 20 h without shaking, and the plates were inspected for bacterial growth inhibition. Fractional inhibitory concentration indices (FICI) were determined post-incubation by the following: $FICI = FIC_A + FIC_B$, with FIC_A = (MIC of compound A in combination/MIC of compound A) and FIC_B = (MIC of compound B in combination/MIC of compound B). Responses were defined as follows: synergistic when the FICI was ≤0.5; indifference when the FICI was >0.5 and ≤4; and antagonistic when the FICI was >4.

6.8 FICI haemolysis assays

Fresh equine blood was centrifuged (1000×g, 10 min) and the supernatant was removed. Harvested erythrocytes were washed three times with PBS and then resuspended to a 5% erythrocyte concentration in PBS. Mixtures of two $[Fe_2L_3]Cl_4$ compounds were prepared by dissolving in PBS to form stock solutions with each compound at a 32-fold greater concentration than in the $FICI_{min}$ well. These stock solutions were used to prepare serial dilutions in 96-well round bottom plates at concentrations ranging from $2\times FICI_{min}$ to $16\times FICI_{min}$ using PBS. The suspended erythrocytes (100 µl) were added to the $[Fe_2L_3]Cl_4$ mixture wells (100 µl) to form concentration ranges equivalent to $FICI_{min}$ to $8\times FICI_{min}$, and the plates were incubated without agitation (310 K, 1 h). All wells were subject to individual visual lysis inspection after the incubation period, and those judged to cause >10% cell lysis were deemed to be to be haemolytic. Controls included a PBS and 1% Triton X-100 as 0 and 100% haemolysis, respectively. Each measurement was performed in triplicate.

6.9 ICP-MS

Overnight cultures of *S. aureus* USA300 and *E. coli* TOP10 were grown in CAMHB (310 K) to exponential phase ($OD_{600} \approx 0.5$). Once at exponential phase, each compound was added to culture (1 ml) to afford the desired compound concentration (8 µg ml^{-1}, MIC or 0.5×MIC) and samples were incubated with shaking (277 K or 310 K, 30 min). For samples incubated at 277 K, cultures were pre-chilled before dosage (277 K, 15 min). Samples were then pelleted by centrifugation (8000×g, 5 min), supernatant removed, and a twofold repeat of resuspension and pelleting was performed. The *S. aureus* pellets were treated with 4% paraformaldehyde and incubated with shaking (277 K, 15 min) before pelleting and removal of the supernatant. Following resuspension of all pellets in PBS, samples were subject to a final centrifugation, removal of supernatant, and the resultant pellets were frozen (253 K) until digestion.

68% v/v nitric acid (300 µL) was used to digest bacteria pellets before the suspensions were heated overnight at 348 K to ensure complete digestion. Each sample was diluted 20-fold using 18.2 MΩ.cm Milli-Q water to attain concentrations of 3.4% v/v nitric acid (total dissolved solids <0.2% w/v). ^{57}Fe calibration solutions were prepared in the range 0.1-1000 ppb using 10 ppm (10 µg ml^{-1}) ^{57}Fe plasma standard solution (Thermo Fisher Scientific) using 3.4% v/v nitric acid. ^{57}Fe accumulation was recorded using an Agilent 7900 ICP-MS spectrometer running in He gas mode.

6.10 CuAAC Fluorescence Confocal Microscopy

An overnight culture of *E. coli* TOP10 or *S. aureus* USA300 was diluted in CAMHB and grown to mid-exponential ($OD_{600} \approx 0.50$) before dosage with either water-solubilised compound to the MIC (or 256 µg ml^{-1} if the MIC value for the compound was >256 µg ml^{-1}) or methanol (negative control) and incubated with agitation (310 K, 30 min). During incubation, the Click-iT® cell reaction mix was prepared from the Click-iT® Cell Reaction Buffer Kit according to the manufacturer's instructions (880 µl Click-iT® cell reaction buffer, 20 µl $CuSO_4$ solution, 100 µl Click-iT® cell buffer additive, 5 µl AF-488 azide). 10 min before the end of the incubation period, 5 µg ml^{-1} of FM 4-64 was added to stain the cell membrane, and the cells (1 ml for each treatment) were collected by centrifugation and fixed with 4% paraformaldehyde (277 K, 15 min). The cells were subsequently washed with PBS, the supernatant was removed, and the pellet was permeabilised with 0.5% Triton X-100 in PBS by incubation with agitation (298 K, 30 min) to enhance AF-488 translocation across cellular membranes. Cells were washed in PBS and in 2% bovine serum albumin (BSA), then resuspended in prepared click reaction mix (180 µl, contains 5 µg ml^{-1} AF-488 azide). Solutions were incubated in the dark (298 K, 30 min), washed with 2% BSA in PBS and stained with 1 µg ml^{-1} DAPI for 1 min. Finally, the cells were washed with PBS, resuspended in PBS, and mounted on slides for microscopy. The slides were prepared using agarose pads prepared with Thermo Scientific™ Gene Frame Seals, to which ~3 µl of sample was added and allowed to dry, followed by addition of 4 µl of SlowFade™ Gold Antifade reagent (Thermo Scientific) to protect fading of the fluorescent dyes during microscopy use. Two independent experiments were performed using each metallohelix. Images were obtained using an LSM510 confocal microscope with Leica X software and analysed with ImageJ and Fiji

software. Images were converted to 16-bit grayscale to facilitate visual interpretation. Image spatial resolution = 60 nm/pixel.

Fluorescence microscopy of L-**9b** was necessary in order to compare results with the previous study[6], and for comparative analysis with the other enantiomer D-**9b**. The benzyl alkyne flexicate L-**9g** recorded MIC values of >256 μg ml^{-1} against *S. aureus* and *E. coli*, and therefore was selected to investigate if the "switch-off" in antimicrobial activity correlated to intracellular accumulation. The inactive parent triplex compounds **11a** (MIC values ≥256 μg ml^{-1}) were of particular interest to explore the relationship between low triplex potency and accumulation, however, unlike the flexicate system, the propargyl triplex compounds **11b** had considerably greater activities against both bacteria (MIC values ≤8 μg ml^{-1}) than the parents. Consequently, the L-**11b** compound was used to assess accumulation of active triplexes and reiterates the unexpectedly large impact that minor structural changes can have upon activity. The benzyl alkyne triplex compounds **11g** were both relatively inactive against *S. aureus* bacteria (MICs of 128 μg ml^{-1}) but exhibited a 64-fold activity difference between enantiomers in *E. coli* growth inhibition assays (with L more active than the corresponding D complex). This made them ideal candidates to assess enantiomeric accumulation variance in the triplex system.

7. Microbiology Results

7.1 Bacterial minimum inhibitory concentration (MIC) determination

Table S1 *In vitro* MIC values of flexicates, triplexes and controls against *S. aureus* strains ATCC 29213 and USA300, and *E. coli* strains ATCC 25922 and TOP10.

Compound	Z =	MIC *S. aureus* / μg ml^{-1}		MIC *E. coli* / μg ml^{-1}	
		ATCC29213	USA300	ATCC25922	TOP10
Λ-**9a**	H	16	16	4	2
Δ-**9a**	H	32	16	8	4
Λ-**9b**	$OCH_2C{\equiv}CH$	8	8	8	8
Δ-**9b**	$OCH_2C{\equiv}CH$	4	4	8	8
Λ-**9c**	O-*n*-C_3H_7	256	128	>256	>256
Δ-**9c**	O-*n*-C_3H_7	>256	256	>256	>256
Λ-**9d**	O-*n*-C_5H_{11}	128	128	>256	>256
Δ-**9d**	O-*n*-C_5H_{11}	128	128	>256	>256
Λ-**9e**	$(OCH_2CH_2)_2OH$	256	256	256	256
Δ-**9e**	$(OCH_2CH_2)_2OH$	>256	>256	>256	256
Λ-**9f**	OBn	16	16	64	16
Δ-**9f**	OBn	32	32	64	32
Λ-**9g**	OBn-C≡CH	>256	>256	>256	>256
Δ-**9g**	OBn-C≡CH	>256	>256	>256	>256
Λ-**10a**	H	16	8	8	8
Δ-**10a**	H	16	8	8	4
Λ-**10e**	$(OCH_2CH_2)_2OH$	>256	>256	>256	256
Δ-**10e**	$(OCH_2CH_2)_2OH$	64	64	128	128
Λ-**10f**	OBn	>256	128	>256	>256
Δ-**10f**	OBn	64	64	>256	>256
Λ-**11a**	H	>256	>256	>256	>256
Δ-**11a**	H	>256	>256	>256	>256
Λ-**11b**	$OCH_2C{\equiv}CH$	256	256	16	8
Δ-**11b**	$OCH_2C{\equiv}CH$	256	128	64	64
Λ-**11c**	O-*n*-C_3H_7	64	64	8	8
Δ-**11c**	O-*n*-C_3H_7	128	128	>256	256
Λ-**11d**	O-*n*-C_5H_{11}	32	64	2	4
Δ-**11d**	O-*n*-C_5H_{11}	32	64	32	32
Λ-**11e**	$(OCH_2CH_2)_2OH$	256	128	>256	256
Δ-**11e**	$(OCH_2CH_2)_2OH$	256	256	>256	256
Λ-**11f**	OBn	8	8	2	2
Δ-**11f**	OBn	8	8	8	8
Λ-**11g**	OBn-C≡CH	128	128	1	0.5
Δ-**11g**	OBn-C≡CH	128	128	64	32
Controls	Ampicillin	-	-	4	2
	Tetracycline	0.5	-	1	1
	Ticarcillin	4	2	8	4

7.2 Bacterial minimum bactericidal concentration (MBC) determination

Table S2 *In vitro* MBC and corresponding MBC/MIC values of flexicates and triplexes against *S. aureus* ATCC 29213 and *E. coli* ATCC 25922 bacterial strains.

Compound	Z =	*S. aureus* ATCC 29213		*E. coli* ATCC 25922	
		MBC / μg ml^{-1}	MBC/MIC	MBC / μg ml^{-1}	MBC/MIC
Λ-**9a**	H	32	2	8	2
Δ-**9a**	H	64	2	32	4
Λ-**9b**	$OCH_2C{\equiv}CH$	16	2	16	2
Δ-**9b**	$OCH_2C{\equiv}CH$	4	1	16	2
Λ-**9d**	O-*n*-C_5H_{11}	256	2	-	-
Δ-**9d**	O-*n*-C_5H_{11}	128	1	-	-
Λ-**9f**	OBn	32	2	128	2
Δ-**9f**	OBn	64	2	128	2
Λ-**10a**	H	32	2	16	2
Δ-**10a**	H	32	2	16	2
Λ-**11b**	$OCH_2C{\equiv}CH$	>256	-	64	4
Δ-**11b**	$OCH_2C{\equiv}CH$	>256	-	>256	-
Λ-**11c**	O-*n*-C_3H_7	128	2	16	2
Δ-**11c**	O-*n*-C_3H_7	>256	-	-	-
Λ-**11d**	O-*n*-C_5H_{11}	64	2	8	4
Δ-**11d**	O-*n*-C_5H_{11}	128	4	128	4
Λ-**11f**	OBn	16	2	8	4
Δ-**11f**	OBn	32	4	32	4
Λ-**11g**	OBn-C≡CH	>256	-	4	4
Δ-**11g**	OBn-C≡CH	>256	-	256	4

7.3 Haemolysis assays

Table S3 *In vitro* haemolytic concentration values of flexicates and triplexes.

Compound	Z =	Haemolytic concentration / μg ml^{-1}
Λ-**9a'**	H	512
Δ-**9a'**	H	512
Λ-**9b'**	$OCH_2C{\equiv}CH$	>512
Δ-**9b'**	$OCH_2C{\equiv}CH$	>512
Λ-**11b'**	$OCH_2C{\equiv}CH$	>512
Δ-**11b'**	$OCH_2C{\equiv}CH$	>512
Λ-**11f'**	OBn	>512
Δ-**11f'**	OBn	256
Λ-**11g'**	OBn-C≡CH	>512
Δ-**11g'**	OBn-C≡CH	>512

7.4 Antimicrobial stability of compounds at different storage durations and temperatures

Table S4 Periodic MIC values of flexicates and triplexes against bacterial strain *E. coli* ATCC 25922 when stored in CAMHB at either 255 K, 293 K or 310 K for incubation periods (IP) of 0, 1, 4, 7, 14 or 28 d.

Compound	Pre-inoculation incubation temp.	*E. coli* ATCC 25922 MIC / μg ml^{-1}					
		0-day IP	1-day IP	4-day IP	7-day IP	14-day IP	28-day IP
Δ-**9a**	255 K	8	8	8	8	4	8
	293 K	8	8	4	8	8	16
	310 K	8	8	8	8	16	16
Λ-**9a**	255 K	4	4	4	4	4	4
	293 K	4	4	4	4	8	8
	310 K	4	4	4	8	8	8
Δ-**11f**	255 K	8	8	4	8	8	8
	293 K	8	8	8	8	8	16
	310 K	8	8	8	16	16	32
Λ-**11f**	255 K	2	2	2	2	2	2
	293 K	2	2	2	2	4	4
	310 K	2	2	2	2	4	4
Δ-**11g**	255 K	64	64	64	64	64	64
	293 K	64	64	32	64	64	64
	310 K	64	64	64	128	128	256
Λ-**11g**	255 K	1	1	1	1	1	1
	293 K	1	1	1	2	1	2
	310 K	1	1	1	1	2	4

7.5 Synergy assays

Λ-**9a**/Δ-**11f** antagonism

Surprisingly, 30% of Λ-**9a**/Δ-**11f** wells were antagonistic. Of the five main types of drug antagonism,[18] the only feasible options are pharmacokinetic and chemical antagonism - both Λ-**9a** and Δ-**11f** are individually active against *E. coli* ATCC 25922 (MICs of 4 μg ml^{-1} and 8 μg ml^{-1}, respectively) thus competitive, non-competitive and physiological are not viable (as two active agents would not produce an overall reduction in potency less than the individual potencies for these three pathways). Ligand exchange between compounds is the only reasonable pathway for chemical antagonism and as such, a mixture of the two compounds was subject to NMR spectroscopy analysis. The combined spectra appeared strictly additive when compared to their individual spectra after incubation in water at (20 h, 37 °C).

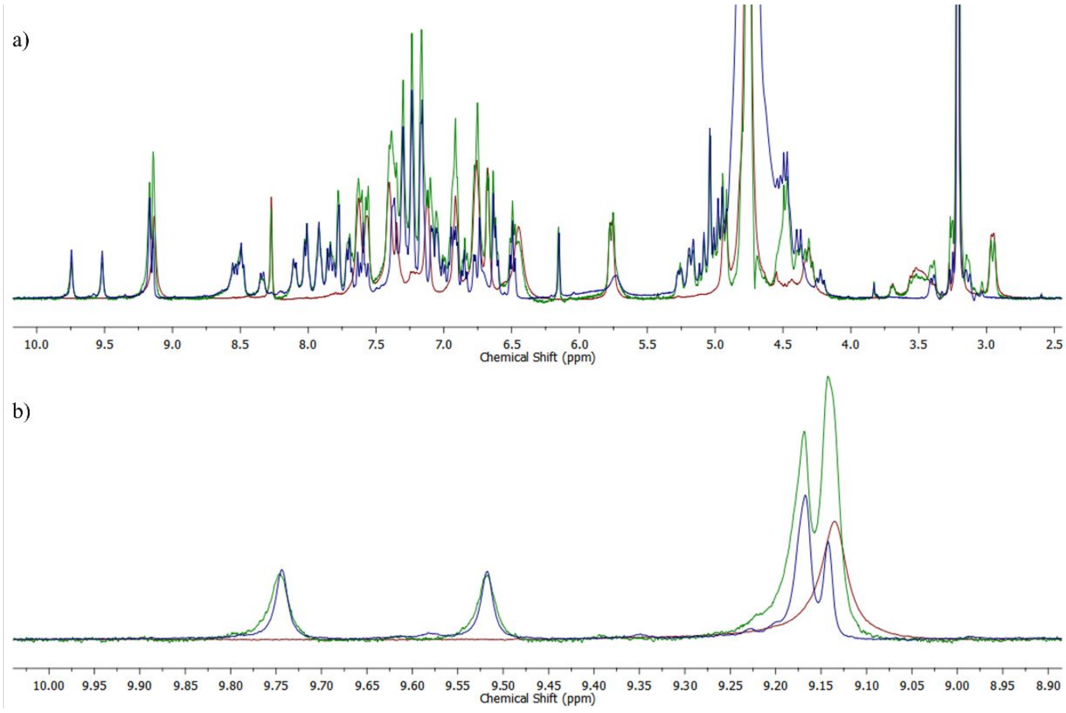

Figure S28 Overlaid post-incubation spectra of Δ-**11f** (blue), Λ-**9a** (red) and Δ-**11f**/Λ-**9a** mixture (green): (a) ^{1}H-NMR spectrum (400 MHz, MeOD, 293 K); (b) enhanced view of ^{1}H-NMR spectrum showing the 9-10 ppm region.

7.6 FICI haemolysis assays

Table S5 *In vitro* haemolysis screening results of metallohelix combinations, with concentrations between $FICI_{min}$ and $8\times FICI_{min}$, with regards to corresponding synergy assays. A positive cell lysis result was recorded if a substantial (>10%) quantity of red blood cells was deemed to have lysed by visual inspection.

Compound combination	Concentration A / µg ml^{-1}	Concentration B / µg ml^{-1}	FICI	Cell lysis
A: Δ-**9b**<br>B: Λ-**9b**	0.5	1	0.50 ($FICI_{min}$)	No
	1	2	1.00 ($2\times FICI_{min}$)	No
	2	4	2.00 ($4\times FICI_{min}$)	No
	4	8	4.00 ($8\times FICI_{min}$)	No
A: Δ-**11b**<br>B: Λ-**11b**	32	0.5	0.31 ($FICI_{min}$)	No
	64	1	0.63 ($2\times FICI_{min}$)	No
	128	2	1.25 ($4\times FICI_{min}$)	No
	256	4	2.50 ($8\times FICI_{min}$)	Yes
A: Δ-**9a**<br>B: Λ-**11g**	0.25	1	0.25 ($FICI_{min}$)	No
	0.5	2	0.50 ($2\times FICI_{min}$)	No
	1	4	1.00 ($4\times FICI_{min}$)	No
	2	8	2.00 ($8\times FICI_{min}$)	No

For two out of three combinations, no red blood cell lysis was observed at any concentrations measured, even at $8\times FIC_{min}$. Pleasingly, this indicates that whilst the combinations have increased potencies against the bacterial strain, no acute increases in erythrocytic cell toxicities occurred. However, the combination of Δ-**11b**/Λ-**11b** did elicit cell lysis, albeit at concentrations 8-fold greater than the $FICI_{min}$. Overall, the favourable synergistic response obtained from combination of these metallohelices outweighs any increase in erythrocyte cytotoxicity.

7.7 ICP-MS

Table S6 Iron (^{57}Fe) cellular accumulation (ng ^{57}Fe per 10^8 cells) in *S. aureus* USA300 bacteria when dosed with select flexicates and triplexes at MIC and 0.5×MIC.

^{57}Fe Compound	*S. aureus* USA300 MIC / µg ml^{-1}	^{57}Fe conc. / ng ^{57}Fe (10^8 cells)$^{-1}$	
		MIC dosage	0.5×MIC dosage
Λ-**9a'**	16	23.1 ± 0.2	11.9 ± 0.3
Δ-**9a'**	16	19.0 ± 0.3	9.55 ± 0.23
Λ-**9b'**	8	38.0 ± 1.0	19.6 ± 0.2
Δ-**9b'**	4	32.8 ± 3.2	16.7 ± 0.1
Λ-**9f'**	16	25.1 ± 0.5	13.8 ± 1.0
Δ-**9f'**	32	22.4 ± 1.8	13.5 ± 1.2
Λ-**9g'**	>256[a]	20.7 ± 2.4	10.7 ± 0.4
Δ-**9g'**	>256[a]	20.1 ± 0.1	12.3 ± 1.0
Λ-**11a'**	>256[a]	4.20 ± 0.50	2.52 ± 0.07
Δ-**11a'**	>256[a]	3.81 ± 0.25	2.05 ± 0.13
Λ-**11b'**	128	15.1 ± 1.0	8.18 ± 0.15
Δ-**11b'**	256	2.58 ± 0.25	1.40 ± 0.09
Λ-**11f'**	8	19.8 ± 0.3	10.8 ± 0.3
Δ-**11f'**	8	5.50 ± 0.21	2.77 ± 0.09
Λ-**11g'**	128	24.8 ± 0.4	13.0 ± 0.3
Δ-**11g'**	128	4.08 ± 0.26	2.26 ± 0.10
Controls	-	0.05 ± 0.02	0.07 ± 0.01

[a] For compounds with MIC values >256 µg ml^{-1}, the MIC dosage was 256 µg ml^{-1} and the 0.5×MIC was 128 µg ml^{-1}.

Table S7 Iron (^{57}Fe) cellular accumulation (ng ^{57}Fe per 10^8 cells) in *E. coli* TOP10 bacteria when dosed with select flexicates and triplexes at 0.5×MIC.

^{57}Fe Compound	*E. coli* TOP10 MIC / μg ml^{-1}	^{57}Fe conc. / ng ^{57}Fe (10^8 cells)$^{-1}$	
		MIC dosage	0.5×MIC dosage
Λ-**9a'**	2	36.0 ± 2.2	18.3 ± 0.2
Δ-**9a'**	4	37.2 ± 2.2	18.8 ± 0.1
Λ-**9b'**	8	50.8 ± 3.5	25.6 ± 0.2
Δ-**9b'**	8	44.0 ± 11.4	27.6 ± 0.3
Λ-**9f'**	64	37.8 ± 2.3	19.2 ± 0.45
Δ-**9f'**	64	42.0 ± 1.0	22.3 ± 1.42
Λ-**9g'**	>256[a]	9.67 ± 1.6	6.82 ± 0.53
Δ-**9g'**	>256[a]	11.1 ± 0.88	9.03 ± 0.39
Λ-**11a'**	>256[a]	7.31 ± 0.14	3.93 ± 0.15
Δ-**11a'**	>256[a]	3.38 ± 0.64	1.85 ± 0.18
Λ-**11b'**	8	23.0 ± 0.9	12.1 ± 0.4
Δ-**11b'**	64	1.75 ± 0.27	0.96 ± 0.12
Λ-**11f'**	2	29.7 ± 0.8	15.7 ± 0.4
Δ-**11f'**	8	4.26 ± 0.43	2.39 ± 0.33
Λ-**11g'**	1	24.7 ± 1.0	13.4 ± 0.5
Δ-**11g'**	64	3.91 ± 0.31	2.33 ± 0.13
Controls	-	0.37 ± 0.08	0.28 ± 0.05

[a] For compounds with MIC values >256 μg ml^{-1}, the MIC dosage was 256 μg ml^{-1} and the 0.5×MIC was 128 μg ml^{-1}.

7.8 CuAAC Fluorescence Confocal Microscopy

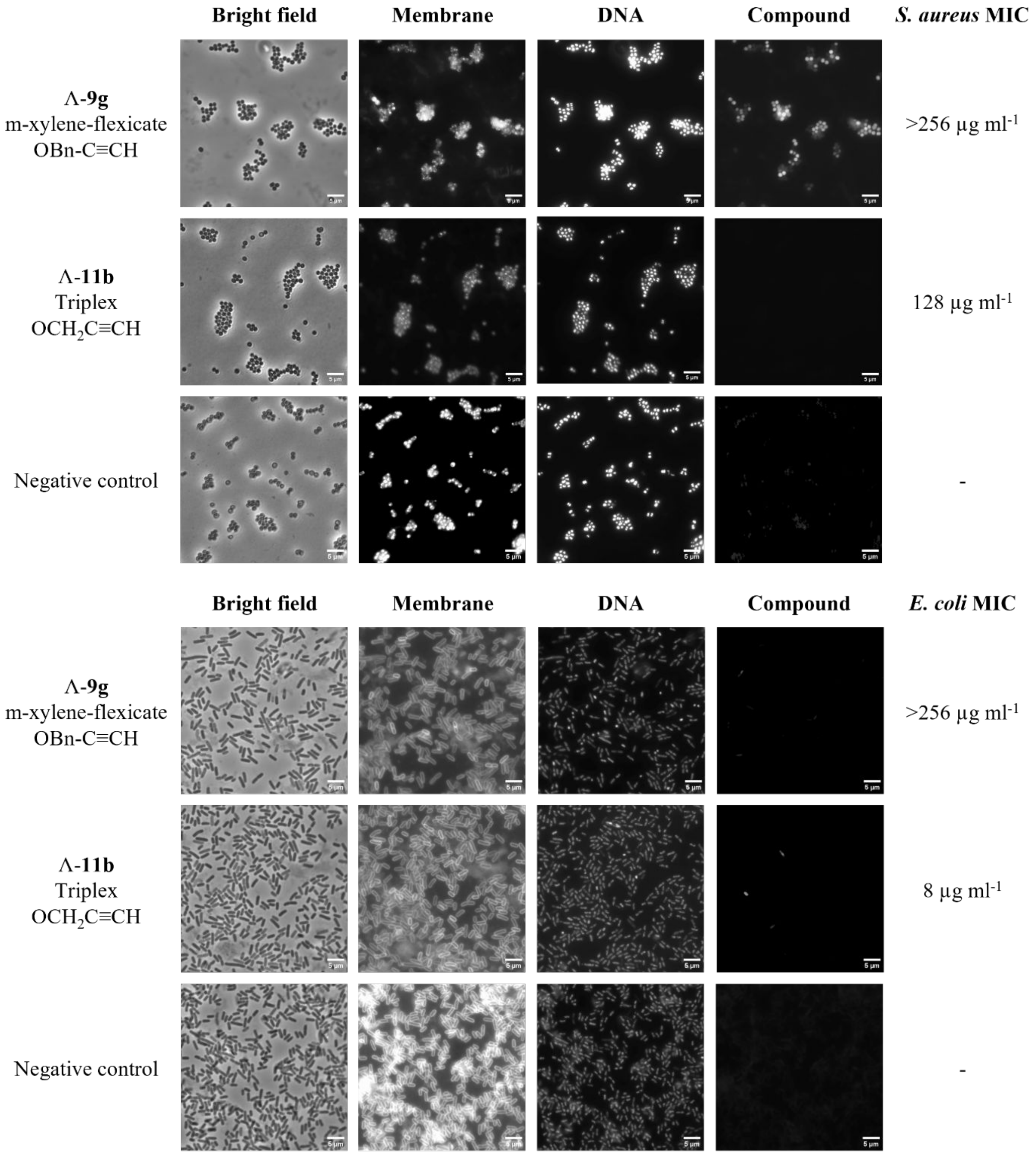

Figure S29 Confocal microscopy images of *S. aureus* USA300 and *E. coli* TOP10 bacteria treated with Λ-**9g** and Λ-**11b** at MIC, and appropriate negative control. Shown are the bright field, membrane stain (FM4-64), DNA stain (DAPI) and compound stain (AF-488) images acquired using an LSM510 confocal microscope and Leica X software. Scale bars = 5 µm.

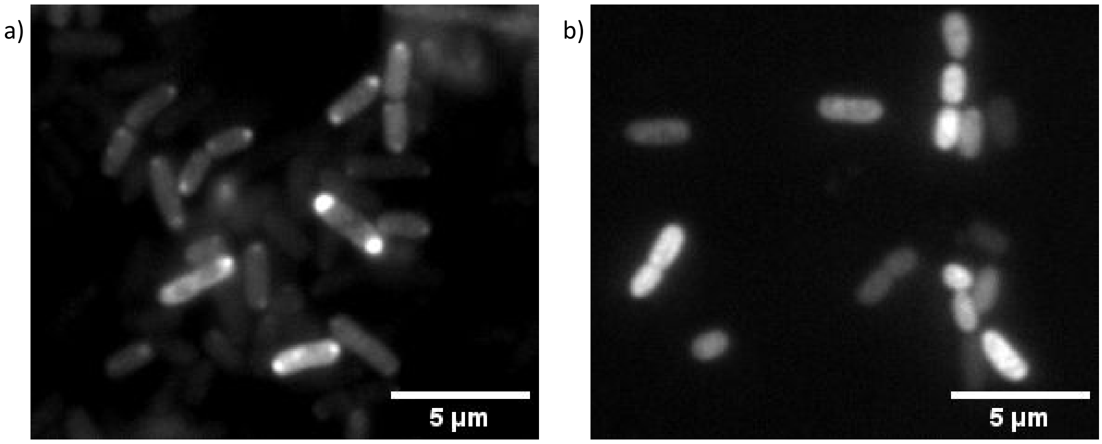

Figure S30 Enhanced confocal microscopy compound stain (AF-488) images of *E. coli* TOP10 bacteria treated with (a) Λ-**9b** and (b) Δ-**9b** at MIC. Images acquired using an LSM510 confocal microscope and Leica X software. Scale bars = 5 µm.

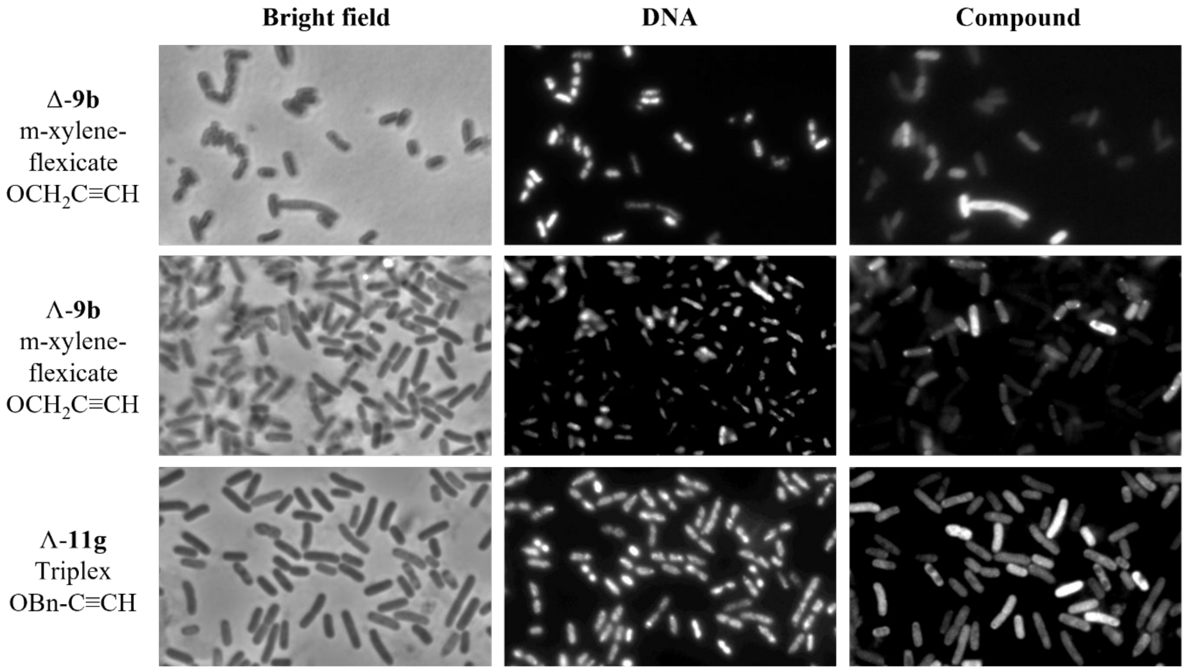

Figure S31. Enhanced confocal microscopy bright field, DNA (DAPI) and compound stain (AF-488) images of *E. coli* TOP10 bacteria treated with **9b** and Λ-**11g** at MIC. Images acquired using an LSM510 confocal microscope and Leica X software.

8. References

(1) Howson, S. E.; Allan, L. E.; Chmel, N. P.; Clarkson, G. J.; Deeth, R. J.; Faulkner, A. D.; Simpson, D. H.; Scott, P. Origins of stereoselectivity in optically pure phenylethaniminopyridine tris-chelates M(NN')3(n+) (M = Mn, Fe, Co, Ni and Zn). *Dalton Trans.* **2011**, *40* (40), 10416-10433.

(2) Seredyuk, M.; Gaspar, A. B.; Ksenofontov, V.; Galyametdinov, Y.; Kusz, J.; Gütlich, P. Does the solid-liquid crystal phase transition provoke the spin-state change in spin-crossover metallomesogens? *J. Am. Chem. Soc.* **2008**, *130*, 1431-1439.

(3) Song, H.; Rogers, N. J.; Allison, S. J.; Brabec, V.; Bridgewater, H.; Kostrhunova, H.; Markova, L.; Phillips, R. M.; Pinder, E. C.; Shepherd, S. L.; et al. Discovery of selective, antimetastatic and anti-cancer stem cell metallohelices via post-assembly modification. *Chem. Sci.* **2019**, *10* (37), 8547-8557.

(4) Das, P.; Ghosh, A.; Kesharwani, M. K.; Ramu, V.; Ganguly, B.; Das, A. ZnII-2,2′:6′,2″-Terpyridine-Based Complex as Fluorescent Chemosensor for PPi, AMP and ADP. *Eur. J. Inorg. Chem.* **2011**, *2011* (20), 3050-3058.

(5) Ballardini, R.; Balzani, V.; Clemente-Leon, M.; Credi, A.; Gandolfi, M. T.; Ishow, E.; Perkins, J.; Stoddart, J. F.; Tseng, H. R.; Wenger, S. Photoinduced electron transfer in a triad that can be assembled/disassembled by two different external inputs. Toward molecular-level electrical extension cables. *J. Am. Chem. Soc.* **2002**, *124* (43), 12786-12795.

(6) Simpson, D. H.; Hapeshi, A.; Rogers, N. J.; Brabec, V.; Clarkson, G. J.; Fox, D. J.; Hrabina, O.; Kay, G. L.; King, A. K.; Malina, J.; et al. Metallohelices that kill Gram-negative pathogens using intracellular antimicrobial peptide pathways. *Chem. Sci.* **2019**, *10* (42), 9708-9720.

(7) Howson, S. E.; Bolhuis, A.; Brabec, V.; Clarkson, G. J.; Malina, J.; Rodger, A.; Scott, P. Optically pure, water-stable metallo-helical 'flexicate' assemblies with antibiotic activity. *Nat. Chem.* **2011**, *4* (1), 31-36.

(8) Faulkner, A. D.; Kaner, R. A.; Abdallah, Q. M.; Clarkson, G.; Fox, D. J.; Gurnani, P.; Howson, S. E.; Phillips, R. M.; Roper, D. I.; Simpson, D. H.; et al. Asymmetric triplex metallohelices with high and selective activity against cancer cells. *Nat. Chem.* **2014**, *6* (9), 797-803.

(9) Koley, D.; Krishna, Y.; Srinivas, K.; Khan, A. A.; Kant, R. Organocatalytic asymmetric Mannich cyclization of hydroxylactams with acetals: total syntheses of (-)-epilupinine, (-)-tashiromine, and (-)-trachelanthamidine. *Angew. Chem. Int. Ed.* **2014**, *53* (48), 13196-13200.

(10) Liu, L.; Long, Q.; Aoki, T.; Zhang, G.; Kaneko, T.; Teraguchi, M.; Zhang, C.; Wang, Y. A Helical Polyphenylacetylene Having Amino Alcohol Moieties Without Chiral Side Groups as a Chiral Ligand for the Asymmetric Addition of Diethylzinc to Benzaldehyde. *Chirality* **2015**, *27* (8), 454-458.

(11) Kaner, R. A.; Allison, S. J.; Faulkner, A. D.; Phillips, R. M.; Roper, D. I.; Shepherd, S. L.; Simpson, D. H.; Waterfield, N. R.; Scott, P. Anticancer metallohelices: nanomolar potency and high selectivity. *Chem. Sci.* **2016**, *7* (2), 951-958.

(12) Clinical and Laboratory Standards Institute (CLSI). *M100: Performance Standards for Antimicrobial Susceptibility Testing*; Clinical and Laboratory Standards Institute, USA, 2024.

(13) Clinical and Laboratory Standards Institute (CLSI). *Methods for Dilution Antimicrobial Susceptibility Tests for Bacteria That Grow Aerobically*; Clinical and Laboratory Standards Institute, USA, 2024.

(14) Rasheed, J. K.; Anderson, G. J.; Yigit, H.; Queenan, A. M.; Domenech-Sanchez, A.; Swenson, J. M.; Biddle, J. W.; Ferraro, M. J.; Jacoby, G. A.; Tenover, F. C. Characterization of the extended-spectrum beta-lactamase reference strain, Klebsiella pneumoniae K6 (ATCC 700603), which produces the novel enzyme SHV-18. *Antimicrob. Agents Chemother.* **2000**, *44* (9), 2382-2388.

(15) Tsukayama, D. T.; Wicklund, B.; Gruniger, R. P. Enterococcus faecium resistant to ampicillin and gentamicin. *Int. J. Antimicrob. Agents* **1992**, *1* (5-6), 239-243.

(16) Zhang, M.; Hong, W.; Abutaleb, N. S.; Li, J.; Dong, P. T.; Zong, C.; Wang, P.; Seleem, M. N.; Cheng, J. X. Rapid Determination of Antimicrobial Susceptibility by Stimulated Raman Scattering Imaging of D2O Metabolic Incorporation in a Single Bacterium. *Adv. Sci.* **2020**, *7* (19), 2001452.

(17) Orhan, G.; Bayram, A.; Zer, Y.; Balci, I. Synergy tests by E test and checkerboard methods of antimicrobial combinations against Brucella melitensis. *J. Clin. Microbiol.* **2005**, *43* (1), 140-143.

(18) Ritter, J.; Flower, R.; Henderson, G.; Loke, Y. K.; MacEwan, D.; Rang, H. How drugs act : General principles. In *Rang & Dale's Pharmacology*, 9th ed.; Elsevier, 2018; pp 6-22.
